# Enzyme-like Acyl Transfer Catalysis in a Bifunctional Organic Cage

**DOI:** 10.1021/jacs.4c03560

**Published:** 2024-06-24

**Authors:** Keith G. Andrews, Tomasz K. Piskorz, Peter N. Horton, Simon J. Coles

**Affiliations:** † Department of Chemistry, University of Oxford, Chemistry Research Laboratory, Oxford OX1 3TA, U.K.; ‡ Department of Chemistry, Durham University, Lower Mount Joy, South Rd, Durham DH1 3LE, U.K.; § UK National Crystallography Service, School of Chemistry, Faculty of Engineering and Physical Sciences, University of Southampton, Southampton, SO17 1BJ, U.K.

## Abstract

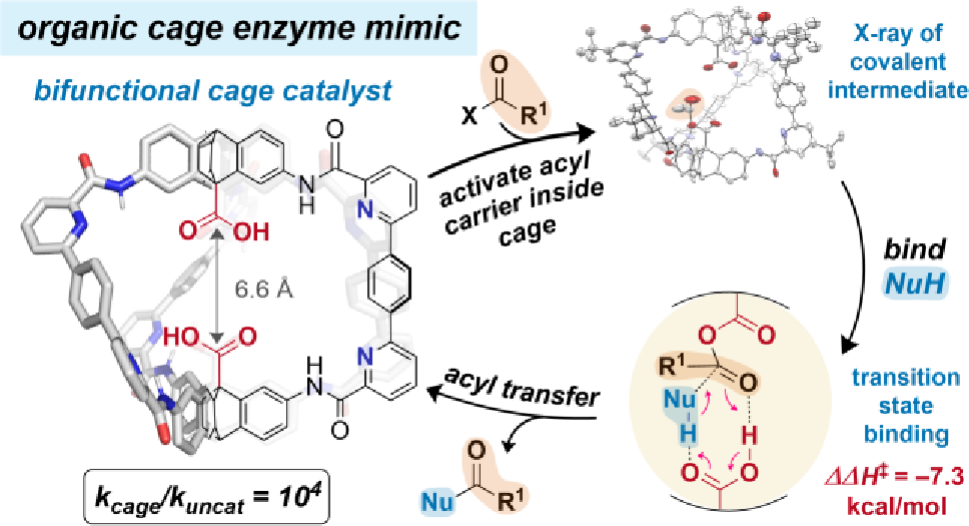

Amide-based organic cage cavities are, in principle, ideal enzyme active site mimics. Yet, cage-promoted organocatalysis has remained elusive, in large part due to synthetic accessibility of robust and functional scaffolds. Herein, we report the acyl transfer catalysis properties of robust, hexaamide cages in organic solvent. Cage structural variation reveals that esterification catalysis with an acyl anhydride acyl carrier occurs only in bifunctional cages featuring internal pyridine motifs and two crucial antipodal carboxylic acid groups. ^1^H NMR data and X-ray crystallography show that the acyl carrier is rapidly activated inside the cavity as a covalent mixed-anhydride intermediate with an internal hydrogen bond. Michaelis–Menten (saturation) kinetics suggest weak binding (*K*_M_ = 0.16 M) of the alcohol pronucleophile close to the internal anhydride. Finally, activation and delivery of the alcohol to the internal anhydride by the second carboxylic acid group forms ester product and releases the cage catalyst. Eyring analysis indicates a strong enthalpic stabilization of the transition state (5.5 kcal/mol) corresponding to a rate acceleration of 10^4^ over background acylation, and an ordered, associative rate-determining attack by the alcohol, supported by DFT calculations. We conclude that internal bifunctional organocatalysis specific to the cage structural design is responsible for the enhancement over the background reaction. These results pave the way for organic-phase enzyme mimicry in self-assembled cavities with the potential for cavity elaboration to enact selective acylations.

## Introduction

**Figure 1:**
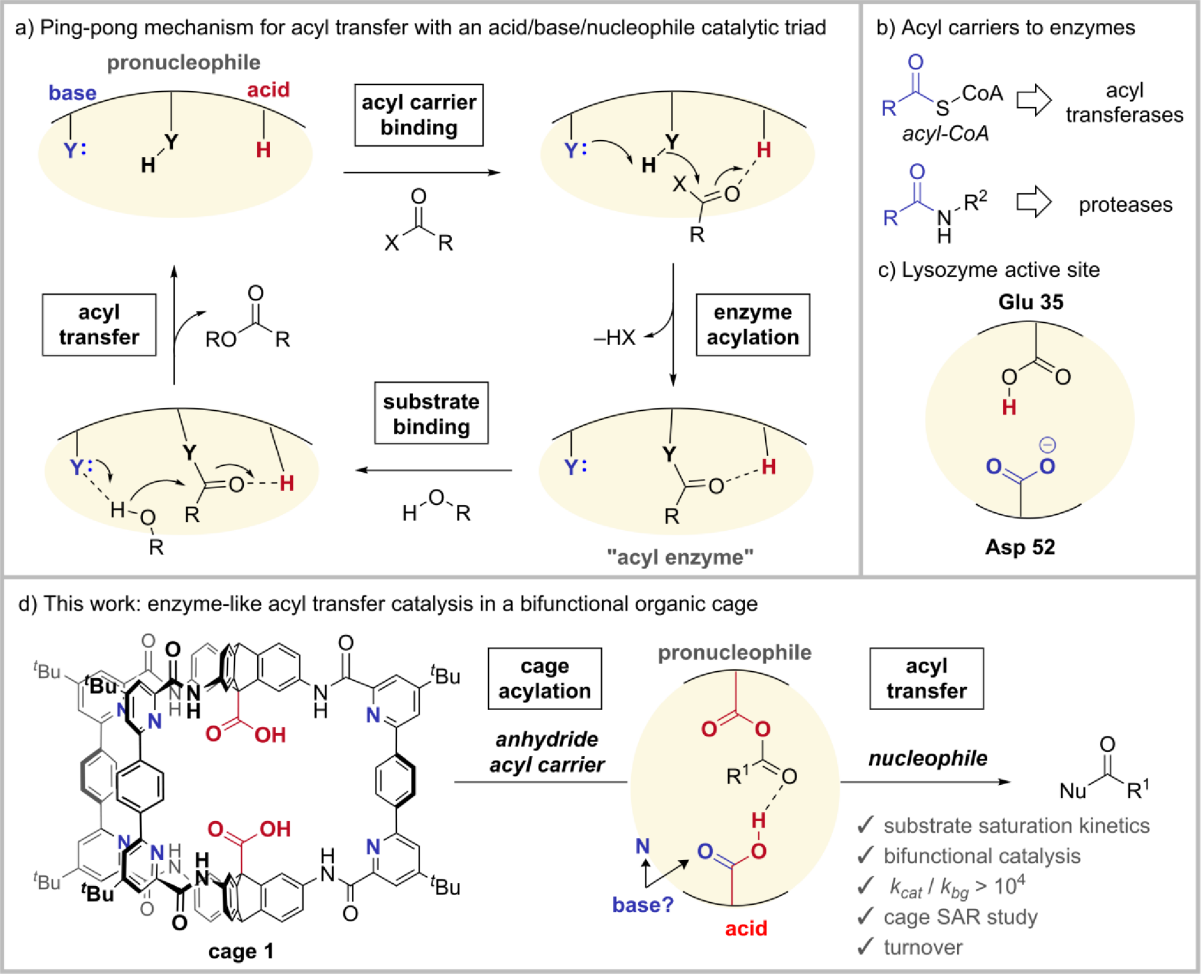
(a) Acyl transfer catalysis in enzymes. (b) Examples of acyl carriers. (c) Bifunctional nature of the lysozyme active site. (d) This work: cage **1** catalyzes acyl transfer reactions from acyl anhydride acyl carriers to alcohol nucleophiles.

Enzyme active sites can be approximated as a cavity in which functionality is organized to accelerate chemical reactions by transition state stabilization.^1−4^ Chemists have explored synthetic cavities as enzyme mimics for decades^4−11^ because such 3D spaces are promising sites to develop efficiency, reactivity, or selectivity not available to small molecule catalysts. Notable catalytic cavity research has explored functionalized cyclodextrin macrocycles,^2,12,13^ other oligomeric macrocycles,^14,15^ dendrimers,^16^ and rigid clefts,^17^ although turnover is not always achieved.^18^ More recently, metal–organic cages^19−26^ and organic capsules^27−35^ have afforded impressive catalytic transformations in noncovalent assemblies. Extrapolating or embedding ligands to approximate cavities around active metals is also pursued.^36,37^ Yet, despite the fact enzyme catalysis largely proceeds via organocatalysis by organic systems, examples of covalent–organic cages^38^ facilitating catalysis are limited to systems that encapsulate nanoparticles^39−42^ or metals,^43,44^ cages with arrays of nonspecific hydrogen bond donors^45^/acceptors,^46^ and hemicryptophane^15^-confined superbases.^47−49^ Unambiguous, cavity-based enzyme-like organocatalysis featuring recognizable bifunctional catalysis modes, cofactors, and covalent and noncovalent activation (e.g., Figure 1a,b) remains unreported.^49−51^

There are several reasons cage organocatalysts are rare. Early work in enzyme mimicry tended to rely on arduous multistep synthesis to install functional groups near binding cavities to enhance rates of reaction by increasing effective molarities.^12,52−54^ In contrast, dynamically self-assembled cages (covalent) and capsules (noncovalent) are easier to access but are either restricted to mild catalysis conditions that do not cause them to disassemble or must undergo a postsynthetic locking procedure^55^ to render them stable, a process scarcely available for noncovalent assemblies.^56^ The cavities must also contain suitable endohedral functionalization^51,57^ to direct substrates or otherwise be restricted to unspecific hydrophobic confinement or proximity-based catalysis^58^ or incremental effects that result from enhanced fragment performance.^48,59^ In our efforts to design stable, soluble organic cages with internal functionality, we recently reported^60^ the synthesis of robust amide-linked organic cages featuring a pair of endohedral antipodal carboxylic acids that resemble aspartyl proteases and glycoside hydrolases (like lysozyme).^61^ This work, in which we oxidatively trap imine assemblies as amide cages *in situ*, extended cage postfunctionalization methodologies developed by Mastalerz,^55,62,63^ which are gaining popularity for accessing functional organic cages.^64^

We now report a bifunctional robust organic cage (Figure 1d) that realizes well-characterized enzyme-like acyl-transfer catalysis^65−71^ contingent on precisely oriented functional groups and an acyl carrier in a fashion reminiscent of the ping-pong mechanism observed in some proteases and transferases (Figure 1a).^1,72,73^ We present NMR analysis and kinetic, crystallographic, and modeling data demonstrating the enzyme-like nature of the process,^53^ including substrate-saturation kinetics, enthalpic stabilization of the transition state, and the role of covalent nucleophilic catalysis. These results demonstrate the wealth of enzymatic principles that can be replicated in functionalized cavities and in organic solvents and the enormous benefits of stable and well-defined organic systems for studying supramolecular catalysis.

## Results and Discussion

### Motivation

The glycoside hydrolase enzyme, lysozyme (Figure 1c), requires a mixed carboxylate/carboxylic acid pair to achieve activity.^74^ More generally, enzymes enlist proximal and confined^75^ basic functionality to promote dynamic formation of carboxylate species that can drive reactivity too slow to occur in the protonated form^53^ or provide electrostatic transition state stabilization.^76^ We therefore sought to synthesize bifunctional cage **1**, which features six internal pyridine groups, held apart from the two carboxylic acid groups, as a possible source of catalytic activity. Crucially, the structurally confined and separable nature of this opposing functionality prevents acid–base neutralization interactions that would predominate with flexible or unconfined functionality.^51^

### Synthesis of Cage 1

**Figure 2:**
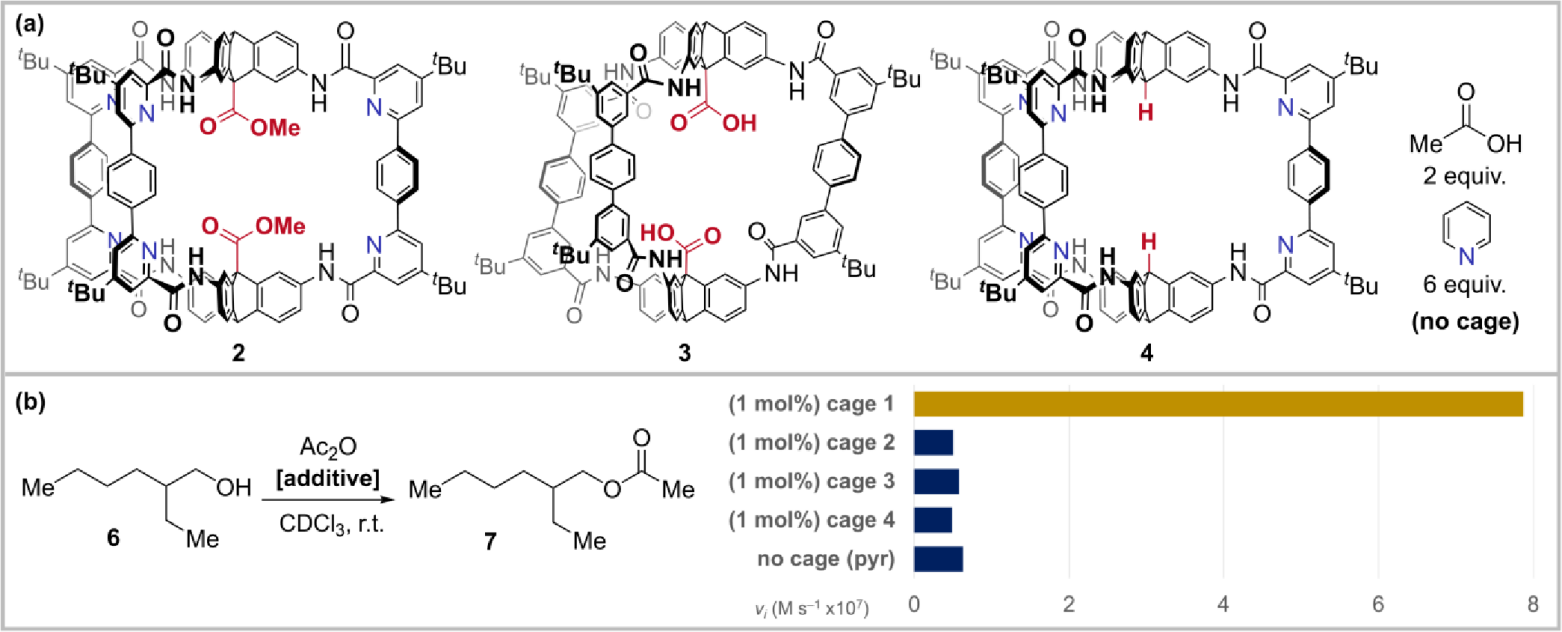
(a) Control group cages with protected acid groups (**2**), acid groups but no pyridines (**3**), pyridines but no acid groups (**4**), and free pyridine and acetic acid (**nocage**). Cage **3** has different amide orientations due to the absence of pyridyl–amide interactions. (b) Proof-of-concept: initial rates (*v*_i_) of acyl transfer from acetic anhydride to alcohol **6** in the presence of different additives show only cage **1** is active.

Cage **1** was accessed by dimethyl ester deprotection of hexapyridine dimethyl ester cage **2** (Figures 2a and S1–S2),^77^ accessed using our previously developed one-pot imine self-assembly/Pinnick oxidation strategy (58–70% **1**, 2 steps).^60^ Two further control cages were synthesized analogously (Figures 2a and S3–S4): diacid cage **3**, which lacks pyridine groups (52%, 2 steps),^60^ and novel cage **4**, which lacks carboxylic acid groups (59%, 1 step). The amide-linked cages are bench-stable solids with good solubility in chloroform; the pyridine cages have low solubility in THF compared to previously reported non-pyridine cage **3**.^60^

### Cage **1** Performs Acyl Transfer Catalysis

Cognizant of the similarities between cage **1** and “multifunctional” enzyme active sites,^78^ we sought to identify possible cage-based catalytic manifolds. Proteases like chymotrypsin^79,80^ cleave peptide bonds via a ping-pong mechanism^81^ in which the enzyme becomes acylated before transferring the acyl group to the nucleophile (water) in a second step (Figure 1a). In contrast, acyl CoA co-enzymes^82^ are acyl carriers and consumed reagents (Figure 1b) that provide reactive acyl groups to active sites, although rarely via a ping-pong mechanism.^73^ Enzyme mimic candidates, such as hexaamide cage **1**, have a clear niche^53^ in that they can tolerate unnatural acyl carrier/coenzyme mimics with poor aqueous stability. We therefore sought to use reactive carboxylic anhydrides in organic solvent to probe acyl transfer catalysis using cages **1**–**4**.

In initial experiments, esterification reactions between alcohol **6** and acetic anhydride were monitored by ^1^H NMR and time/conversion data recorded with and without cage additives (Figure 2b). Alcohol **6** was selected as a readily available, nonvolatile, and easy-to-dry primary alcohol. In the absence of additives, acetic anhydride undergoes slow esterification with **6** in CDCl_3_ at 25 °C. In contrast, when cage **1** (1 mol %) is included as an additive, esterification is accelerated (Figures 2b and S7). Ester-protected cage (**2**) and cages lacking internal pyridine groups (**3**) or acid motifs (**4**) show no rate acceleration compared to the background reaction under otherwise identical conditions. “No-cage” reactions with free pyridine, acetic acid, or both in analogous amounts also showed negligible acceleration (Figure 2b and Table S1). We therefore set out to understand the origin of the acyl-transfer rate acceleration observed with cage **1**.

### Acyl Transfer Rate Enhancements with Cage **1** Are Substrate Dependent

To investigate substrate requirements for catalysis, the anhydride was varied with R^2^ = Me, Et, ^*i*^Pr, and ^*t*^Bu. Likewise, the alcohol was varied with R^1^ = isoamyl, 2-ethylhexyl, isopropyl, ^*t*^butyl, and phenyl. Second-order initial rate constants were extracted from full ^1^H NMR kinetic data profiles (Figures S8–S9) and enhancements relative to background calculated after subtraction of background contributions (Table S1).

All anhydrides and alcohols investigated showed cage **1**-promoted esterification catalysis (Tables 1 and S2). Significant structure–activity variation is observed for both anhydride and alcohol in both background and cage-catalyzed reactions. For the background reaction, the alcohol identity dominates variation in the rate constant, consistent with sterically penalized alcohol organization/proton transfer in the transition state (*vide infra*, Figure 7b), whereas the rate constants vary only over a single order of magnitude with changing anhydride identity.

**Table 1:**
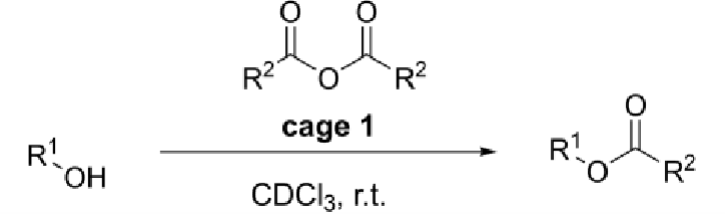
Substrate and Acyl Anhydride Dependent Rate Enhancements for Esterification Catalysis by Cage **1**^a^

	(R^2^CO)_2_O	R^1^OH	*k*_bg_	*k*_cage_	*k*_cage_/*k*_bg_
a	Me	2-Et-hexyl	9.4 × 10^–6^	1.4 × 10^–1^	15400
b	Et	2-Et-hexyl	1.0 × 10^–5^	4.6 × 10^–2^	4470
c	^*i*^Pr	2-Et-hexyl	9.8 × 10^–6^	6.3 × 10^–2^	6440
d	^*t*^Bu	2-Et-hexyl	2.9 × 10^–6^	3.3 × 10^–4^	117
e	Me	isoamyl	1.3 × 10^–5^	1.6 × 10^–1^	12400
f	Me	^*i*^Pr	2.5 × 10^–6^	3.8 × 10^–2^	15200
g	Me	^*t*^Bu	3.7 × 10^–8^	2.9 × 10^–4^	7760
h	Me	Ph	2.2 × 10^–4^	4.3 × 10^–1^	1930

a Rate constants, *k* (M^–1^ s^–1^), are extracted from second-order equations as follows: background rate: *k*_bg_ = d[ester]/d*t*/([R^1^OH][(R^2^C=O)_2_O]); *k*_cage_ = d[ester]/d*t*/([alcohol][cage]); cage 1.69 mM (25 mol % with respect to alcohol), alcohol 6.75 mM, Ac_2_O 135–159 mM, CDCl_3_, 298 K.

In contrast, in the cage-catalyzed reaction, the anhydride identity more strongly affects the rate enhancement, suggesting a key ordering of this component in the cavity in the rate-determining step. The steric nature of the alcohol contributes to the rate similarly in the cage-promoted reaction as for the background. Isopropanol and isopropyl anhydride apparently benefit from marginally favorable alignments inside the cage compared to the background reactions, resulting in inflated rate constant enhancement ratios, *k*_cage_/*k*_bg_.

### Reaction of Cage **1** with Acyl Anhydrides

**Figure 3:**
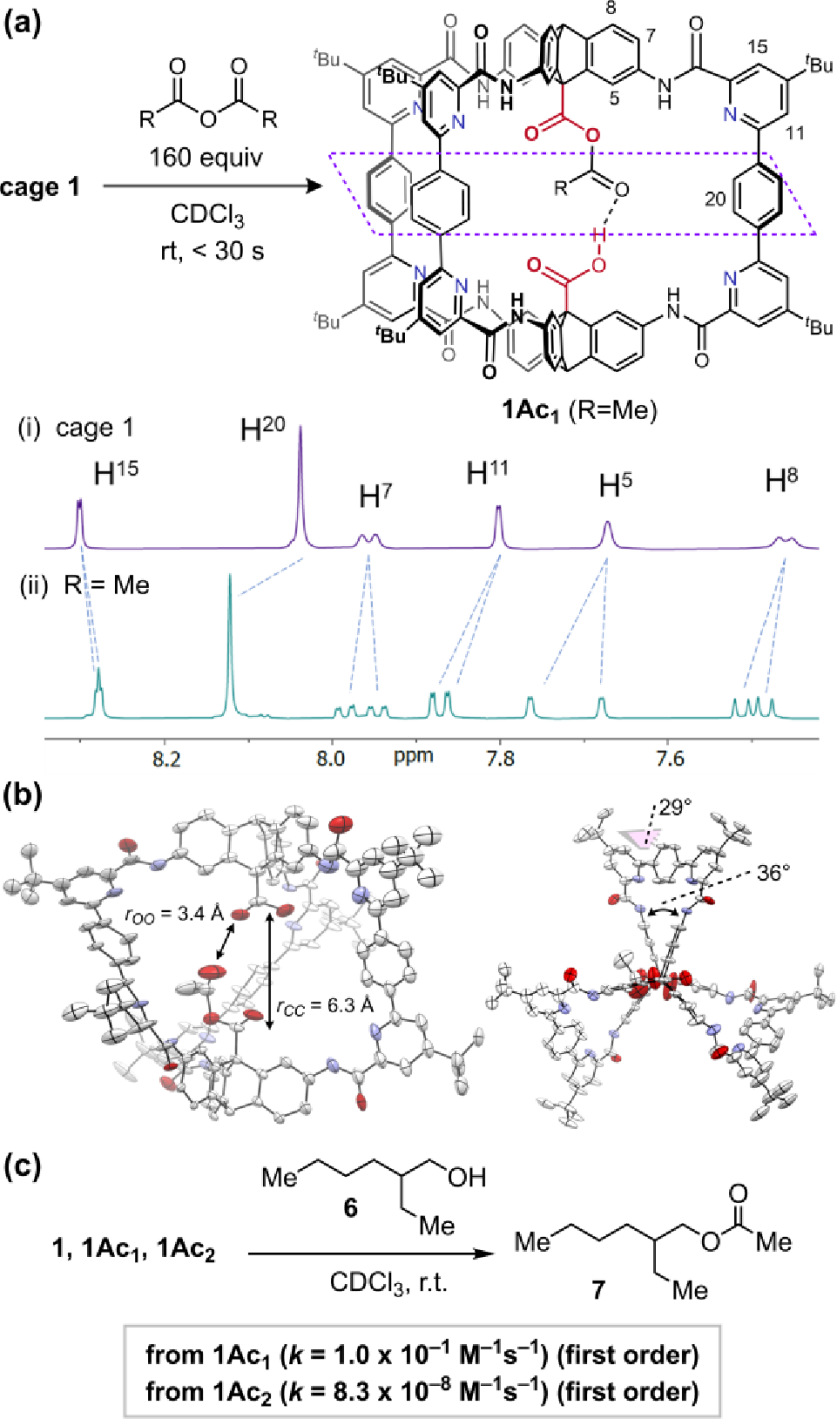
(a) Various carboxylic anhydrides react with **1** to form monoacylated cage anhydrides. (b) Crystal structure of monoacetylated cage **1Ac**_**1**_; side view and top view. (c) **1Ac**_**1**_ is the active acylating agent when esterification is monitored in the absence of Ac_2_O.

We next set out to discover how the cage interacts with the acyl anhydride. Addition of 160 equiv of acetic anhydride to a solution of cage **1** (1.69 mM) in CDCl_3_ resulted in full conversion (<30 s) by ^1^H NMR analysis to a single cage species with each initial proton environment split into two signals, indicating desymmetrization about the equatorial plane by monoacylation at the acid group (**1Ac**_**1**_) (Figure 3a).

Propionic, isobutyric, and pivalic anhydrides reacted analogously (Figure S10) over the same time scale (second-order rate constants >0.25 M^–1^ s^–1^) with distinct signals for the internal anhydride R groups. Over a longer period of time, the bisacylated cage species **1Ac**_**2**_ (in which both cage carboxylic acids are acylated) also forms. To gain further insight into the proposed monoacylated cage **1Ac**_**1**_, we prepared and analyzed a crystal structure.

### Crystal Structure of Cage **1Ac**_**1**_

A crystal structure was obtained of the monoacetylated cage **1Ac**_**1**_ by vapor diffusion of *n*-pentane into a solution of dichloromethane/acetic anhydride (Figures 3b and S11). Cage **1Ac**_**1**_ crystallized in the triclinic space group *P*1 with two cage molecules in the unit cell. Computed models convincingly indicate the presence of an internal hydrogen bond between the acyl group and the remaining acid group (*vide infra*). Although in the crystal structure of **1Ac**_**1**_ the acyl group carbonyl projects toward the remaining acid group in one of the cage units (*r*_O–O_ = 3.4 Å), the significant disorder in the cavities hindered direct analysis of the suspected hydrogen bond. Instead, a structural comparison of **1Ac**_**1**_ with cage **2** is informative. Like cage **2**,^77^ in the crystal structure of cage **1Ac**_**1**_, all amide carbonyl units have externally projected oxygen atoms (in contrast to cage **3**)^60,83^ as a result of pyridine/amide interactions (the pyridine lone pair interacts more favorably with the amide NH group than the amide carbonyl).^77,84^ Despite this similarity (Figure S11), compared to cage **2**, cage **1Ac**_**1**_ shows an increased average biaryl dihedral angle in the terphenyl units (**1Ac**_**1**_: 29°; **2**: 25°), which is required for axial twisting^77^ (**1Ac**_**1**_: 36°; **2**: 34° about the triptycene axis, Figure 3b), which results in a slight contraction of acid–acid carbon–carbon distance *r*_CC_ in **1Ac**_**1**_ (**1Ac**_**1**_: 6.3 Å; **2**: 6.6 Å).^77^ The chemical shift deshielding for spectator protons H^20^ and H^11^ in **1Ac**_**1**_ in the solution-phase NMR data (Figure 3a) is also consistent with biaryl twisting (and therefore axial twisting and cage contraction). These data indicate a cage height contraction in **1Ac**_**1**_ compared to **2**, consistent with the proposed hydrogen bond in **1Ac**_**1**_.

### Cage **1Ac_1_** Is the Active Acyl Transfer Species

The reactivity of acylated cages was studied in the absence of exogenous acylating agents. A mixture of cage **1**, monoacylated cage **1Ac**_**1**_, and bisacylated cage **1Ac**_**2**_ was prepared by mixing an excess of Ac_2_O with cage **1** in CDCl_3_ and then evaporating all liquids, including AcOH and excess Ac_2_O, under high vacuum. Now, when alcohol **6** (23 mM, >30 equiv relative to **1Ac**_**1**_) was added to a CDCl_3_ solution of the cages (1.7 mM total), esterification could be directly monitored by ^1^H NMR comparing consumption of the two cage species (Figures 3c and S12–S13). Acylation is mediated primarily from **1Ac**_**1**_ (first order in **1Ac**_**1**_, *k* = 1.0 × 10^–1^ M^–1^ s^–1^, in agreement with normal catalysis conditions, Figure S14). Acylation from **1Ac**_**2**_ is also first order but is significantly slower (*k* = 8.3 × 10^–8^ M^–1^ s^–1^, Figure S15). This data shows that **1Ac**_**2**_ is significantly less reactive than Ac_2_O (i.e., the background reaction, *k* = 10^–5^ M^–1^ s^–1^). The enhanced stability of **1Ac**_**2**_ made the enhanced reactivity of **1Ac**_**1**_ even more intriguing, and we next examined the formation of this species in greater detail.

### Acyl Transfer Reactions Both to and from Cage **1** Are Accelerated

Propionic (propanoic) acid reacts slowly with acetic anhydride (CDCl_3_, 298 K), eventually reaching an equilibrium mixture of the three expected anhydride species. Equilibrium is reached significantly faster in the presence of cage **1** (50 min) than without (>7 h) (Figure S16–S19). Two forms of rate enhancement are therefore operative in cage **1**: (i) enhanced acylation of the cage to form an “acyl enzyme” equivalent, **1Ac**_**1**_, and (ii) enhanced acyl transfer from **1Ac**_**1**_ to nucleophilic substrates (such as propionic acid or alcohol **6**). Inspired by Figure 1a, we next probed the possibility of substrate binding in the cage cavity.

### Saturation Kinetics Indicate an Alcohol–Cage Complex

**Figure 4:**
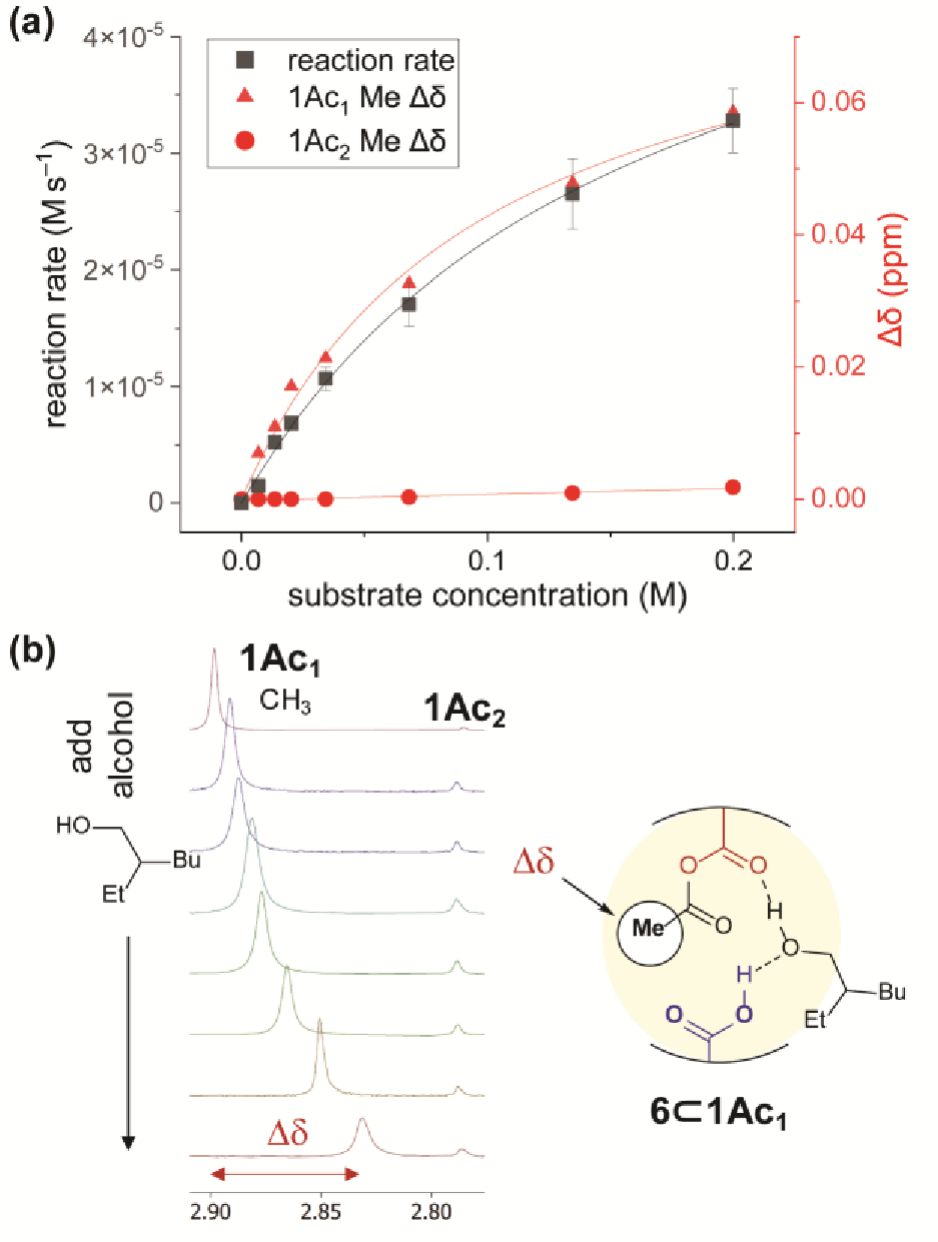
(a) Plotting initial esterification rates in the presence of cage **1** (1.67 mM) against varying substrate (alcohol **6**) concentration shows Michaelis–Menten saturation kinetics. (b) In the same experiments, the **1Ac**_**1**_ acetyl group Me (CH_3_) ^1^H NMR shift is displaced dependent on alcohol concentration (**1Ac**_**2**_ is relatively unaffected), suggesting alcohol binding close to the anhydride.

Another feature of enzyme catalysis is formation of an enzyme–substrate complex. The substrate is first bound (with affinity often interpreted using the Michaelis constant, *K*_M_) before the enzyme–substrate complex undergoes a pseudo-first-order reaction to form product.^53^ Esterification reactions between different concentrations of alcohol **6** and an excess of acetic anhydride in the presence of cage **1** at constant concentration were measured by ^1^H NMR, and the background contributions were accounted for (Table S3). The initial rates of ester formation show a strong deviation from first-order alcohol dependency when plotted as a function of substrate (alcohol) concentration (Figure 4a).

The data could be fitted to a Michaelis–Menten saturation kinetics model, consistent with the formation of an enzyme-like precomplex of cage and alcohol (*K*_M_ = 1.6 × 10^–1^ M; *V*_max_ = 5.9 × 10^–5^ M s^–1^; *k*_cat_ = 3.5 × 10^–2^ s^–1^; Figure S20). This data implies weak binding (∼1 kcal/mol) of the alcohol to form an “enzyme–substrate” complex before catalysis.

A second observation corroborates the hypothesis of alcohol binding. When increasing concentrations of alcohol are added to a mixture of **1Ac**_**1**_ and **1Ac**_**2**_, the ^1^H NMR signal of the internal anhydride methyl (CH_3_) group of **1Ac**_**1**_ undergoes a large, concentration-dependent shift, Δδ (Figure 4b). Significantly, this dependency correlates exactly with the Michaelis–Menten rate profile when overlaid and normalized (Figure 4, red triangles). Importantly, the bisacyl species **1Ac**_**2**_ showed no such response (red circles), and control experiments rule out shift dependency on acetic acid concentration (Figure S21). Taken together with molecular modeling results (*vide infra*, Figure S35), we interpret this data to imply weak binding of a single alcohol close to the anhydride of **1Ac**_**1**_ prior to esterification, potentially disrupting the original cage intramolecular hydrogen bond. To probe the requirement for the second carboxylic acid, an additional cage was synthesized.

### Low-Symmetry Amide Cages Can Be Separated by Size-Exclusion Chromatography

**Figure 5:**
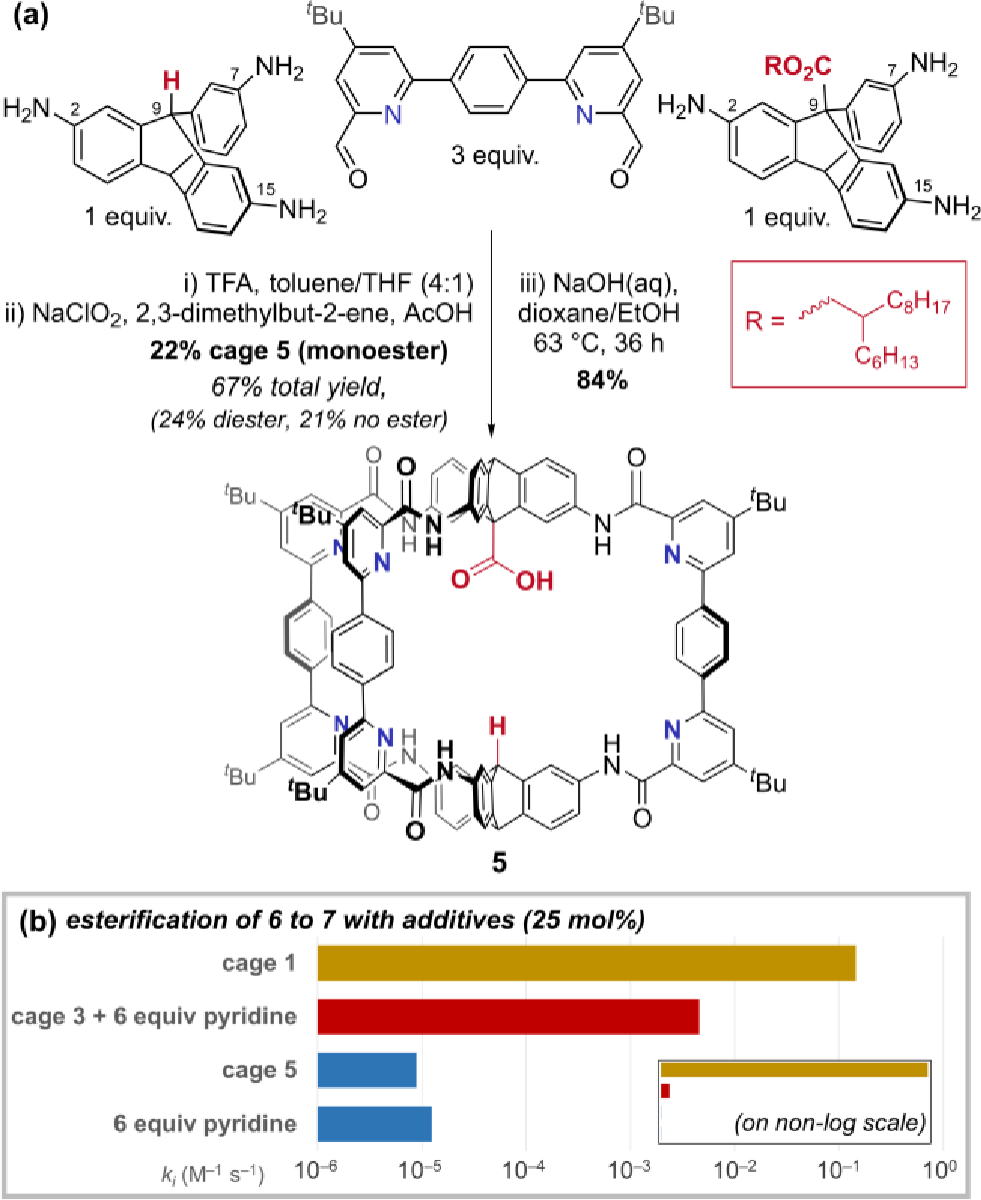
(a) Access to equatorially unsymmetric monoacid cage **5** by statistical synthesis and size-exclusion chromatography using a large, cleavable ester. (b) Cage **5** is inactive as an esterification catalyst. Cage **3** becomes a modest catalyst if pyridine (6 equiv with respect to cage) is present (second-order rate constants shown).

Symmetry reduction of self-assembled organic cages remains rare and valuable.^77^ The control cage molecule **5** (Figure 5a), which is desymmetrized about the equatorial plane and has only one carboxylic acid, was synthesized by statistical reaction with two different triptycene precursors.

Unlike the intermediate imine cages, the oxidized amide cages are stable to separation by standard recycling gel-permeation chromatography. The choice of a large alkyl ester group made the three statistically formed products separable by size (Figures S5–S6), and the desired mixed cage was isolated in 22% yield after recycling GPC (gel permeation chromatography). Unlike cage **2**, hydrolysis of the monoalkyl ester cage to give monoacid cage **5** required heating, presumably due to the increased steric and hydrophobic nature of the cavity. Cage **5** was then examined for any catalytic activity.

### Systems to Test the Critical Role of Bifunctionality

Two cage systems were analyzed to probe the requirement for bifunctionality: first, monoacid cage **5** (1.67 mM), which tests the requirement for a second internal acid group, and, second, non-pyridine-containing cage **3** (1.67 mM) with and without 6 equiv of free pyridine per cage (10 mM), which probes the role of the pyridine groups in cage **1**.

### Role of Cage Bifunctionality in Esterification from Acetylated Cage

Two critical observations are made. First, monoacid cage **5** does not catalyze acyl transfer to alcohol **6** (6.68 mM), even in its acylated state (Figures 5b and S29). Second is the observation that when exogenous pyridine (10 mM, 6 equiv with respect to cage) is added to catalysis conditions with inactive (Figure 2b) non-pyridine cage **3**, catalysis is activated, with a modest rate constant enhancement (*k*_cage_/*k*_bg_ = 5 × 10^2^; contrast for cage **1**, *k*_cage_/*k*_bg_ = 1.5 × 10^4^) (Figures 5b and S29). Other additives provide valuable data, too: in cage **1** promoted esterifications, addition of acetic acid inhibits catalysis somewhat (Figure S22), addition of ester product promotes a marginal rate enhancement (Figure S22), and the addition of pyridine causes a small rate enhancement (Figure S24). Together, these observations demonstrate that the acyl transfer catalysis reaction is contingent on the second carboxylic acid group but requires basic functionality, too; i.e., a bifunctional system is required.

### Role of the Bifunctionality in Cage Acetylation

**Figure 6:**
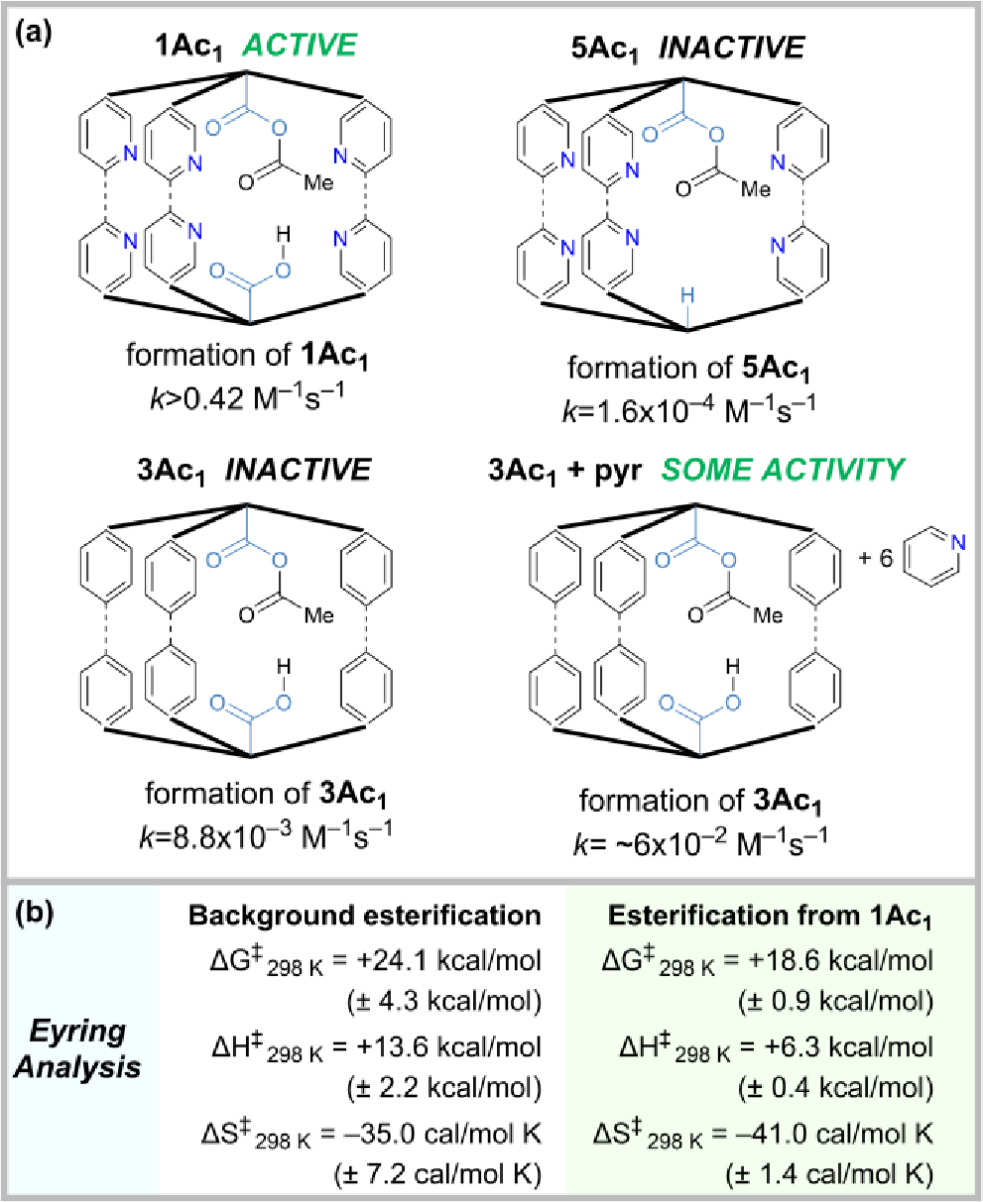
(a) Critical role of bifunctionality in promoting catalysis with cage **1**. The combination of diacid motif and pyridine is necessary to achieve rate acceleration of both acyl cofactor activation and esterification catalysis. Second-order rate constants are shown for formation of monoacylated cages with Ac_2_O. (b) Eyring analysis of the esterification reaction.

Monoacid hexapyridine cage **5** reacts sluggishly with acetic anhydride to form **5Ac**_**1**_ (*k*_cage1_ > 10^3^ × *k*_cage5_, Figure S26), demonstrating the requirement of a second cage carboxylic acid group in promoting activation of the anhydride for reaction with the cage. Also supporting this conclusion is the observation that non-pyridine diacid cage **3** does react somewhat quickly with acetic anhydride (*k*_cage1_ ≈ 40 × *k*_cage3_). The formation of **3Ac**_**1**_ can be marginally accelerated by the addition of 6 equiv of free pyridine (*k*_cage3+pyr_ ≈ 7 × *k*_cage3_) (Figures 6a and S27–S28). As for the esterification step, this data implies an essential role for the second acid group and a supportive role for base in promoting cage acylation from the anhydride. A summary of acyl anhydride activation and esterification catalysis with the key cage systems is shown in Figure 6a.

### Eyring Analysis

Both the **1Ac**_**1**_-catalyzed (1.69 mM) and background (pyridine, 10 mM) esterification reactions with acetic anhydride (159 mM) and alcohol **6** (6.7 mM) in CDCl_3_ were performed at five different temperatures (293, 298, 303, 308, and 313 K) *in situ* in a pre-equilibrated NMR probe and initial rates measured. The second-order rate constants *k*_cage_ and *k*_bg_ were plotted according to the Eyring equation to obtain activation barrier data for the esterification part of the reaction (Figures 6b and S30–S32, Table S4). The background reaction has an activation free energy barrier Δ*G*^‡^_bg_(298 K) = +24.1 kcal/mol, with Δ*H*^‡^_bg_ = +13.6 kcal/mol and Δ*S*^‡^_bg_ = −35.0 cal/(mol K). The catalyzed reaction has Δ*G*^‡^_cage_(298 K) = +18.6 kcal/mol, with Δ*H*^‡^_cage_ = +6.3 kcal/mol and Δ*S*^‡^_cage_ = −41.0 cal/(mol K). A negative entropy of activation typically indicates an associative rate-determining step, which is known for esterification reactions to be nucleophilic attack on the anhydride carbonyl by the alcohol. For the cage-catalyzed reaction, there may be a slight additional entropic organizational cost compared to the background. The cage-promoted rate acceleration is therefore entirely provided by enthalpic stabilization of the transition state (i.e., transition state binding).^53^ These observations are consistent with both (i) initial alcohol binding in the cavity and (ii) stabilization of a highly ordered transition state which, by Hammond’s postulate, resembles the tetrahedral intermediate.

### Computational Modeling

**Figure 7:**
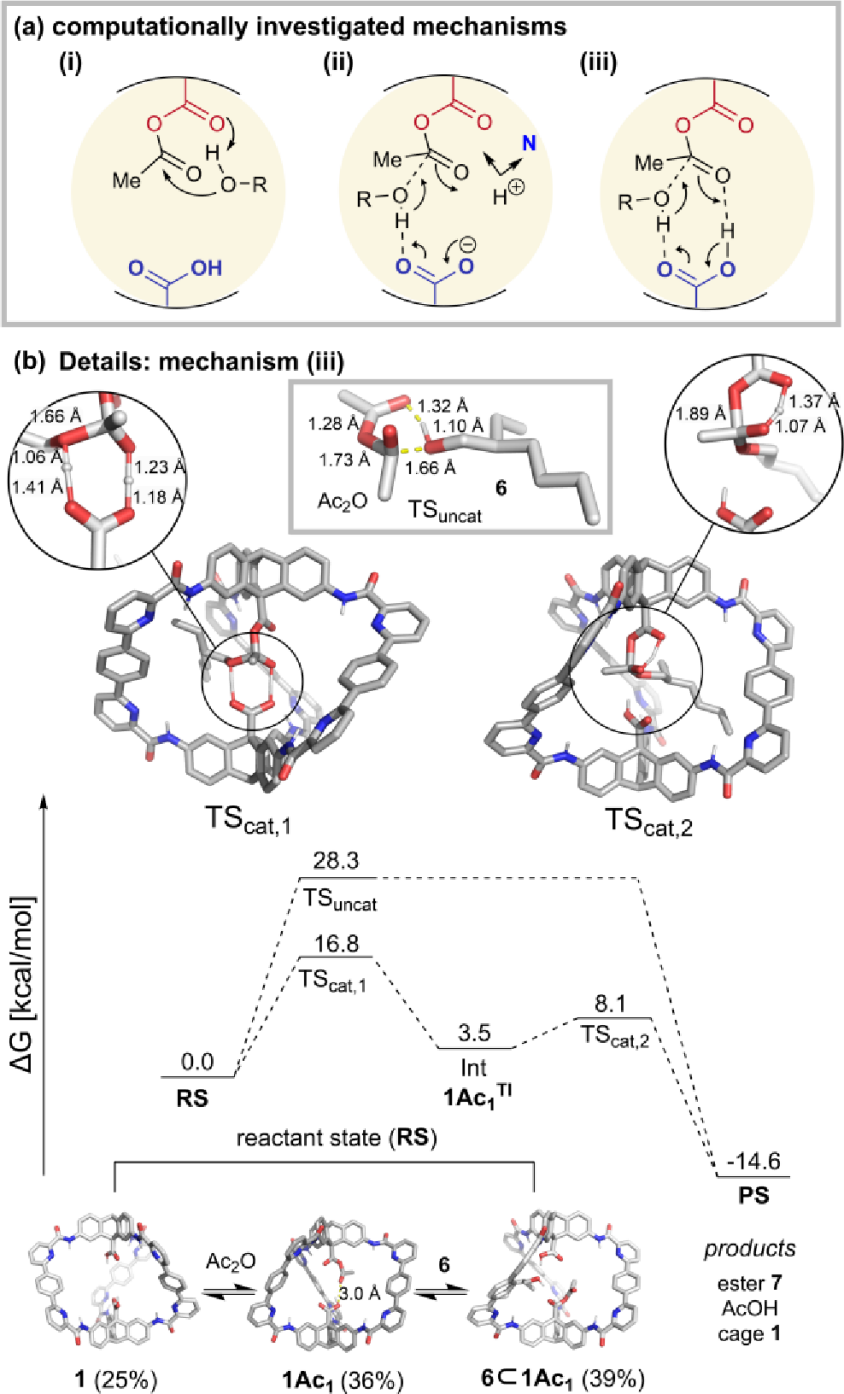
(a) Three plausible mechanisms investigated for cage catalysis. (b) Free energy profile of uncatalyzed and catalyzed (mechanism (iii)) reactions at the CPCM(CHCl_3_)-M06-2X/def2-TZVP//CPCM(CHCl_3_)-PBE0-D3BJ/def2-SVP level of theory.

Our experimental kinetic data show acyl transfer from **1Ac**_**1**_ to the alcohol to be rate-limiting, and we investigated three mechanistic pathways for this step using density functional theory (DFT) calculations (Figure 7a):

(i) The same transition state as the background reaction (Figure S36) occurs in the cage, which provides a stabilizing field compared to bulk solvent (Figure 7a-i).
(ii) A cage carboxylate, formed by proton transfer to a cage pyridine or anhydride, accelerates deprotonation of the alcohol as it attacks the acyl group (Figure 7a-ii).^85−87^
(iii) The cage carboxylic acid group promotes simultaneous deprotonation of the alcohol nucleophile and protic activation of the reacting acyl group in a cyclic transition state (Figure 7a-iii).

DFT calculations show **1**, **1Ac**_**1**_, and **6⊂1Ac**_**1**_ are within 1 kcal/mol energetically, consistent with experiment, which indicates exchange between these species. For mechanism (i), DFT calculations found a similar activation barrier (Δ*G*^‡^_cage_ = 25.1 kcal/mol) to the uncatalyzed reaction (Δ*G*^‡^_bg_ = 28.3 kcal/mol) (Figure S37). In the case of mechanism (ii), the formation of a zwitterionic cage by proton transfer to a cage pyridine is computed to require 27.9 kcal/mol, and an additional 14.8 kcal/mol is needed to reach the transition state. Although our searches for alternative stabilized zwitterion formulations were unsuccessful (Figure S38), we cannot fully rule out pathways utilizing carboxylate character. We note this because the observations of acid inhibition and base acceleration are consistent with a carboxylate promoted mechanism. For mechanism (iii), an activation barrier ∼11.5 kcal/mol lower than the background reaction is calculated (Figure S39). The transition state free energy has a low enthalpic contribution due to short strong hydrogen bonds, which prevent charge buildup. The close agreement between the computational (Δ*G*^‡^_cage_ = 16.8 kcal/mol) and experimental (Δ*G*^‡^_cage_ = 18.6 kcal/mol) activation barrier values at 298 K suggests reaction mechanism (iii) is highly plausible (Figure 7b).

### Overall Proposed Mechanism of Acyl Transfer Catalysis by Cage **1**

**Figure 8:**
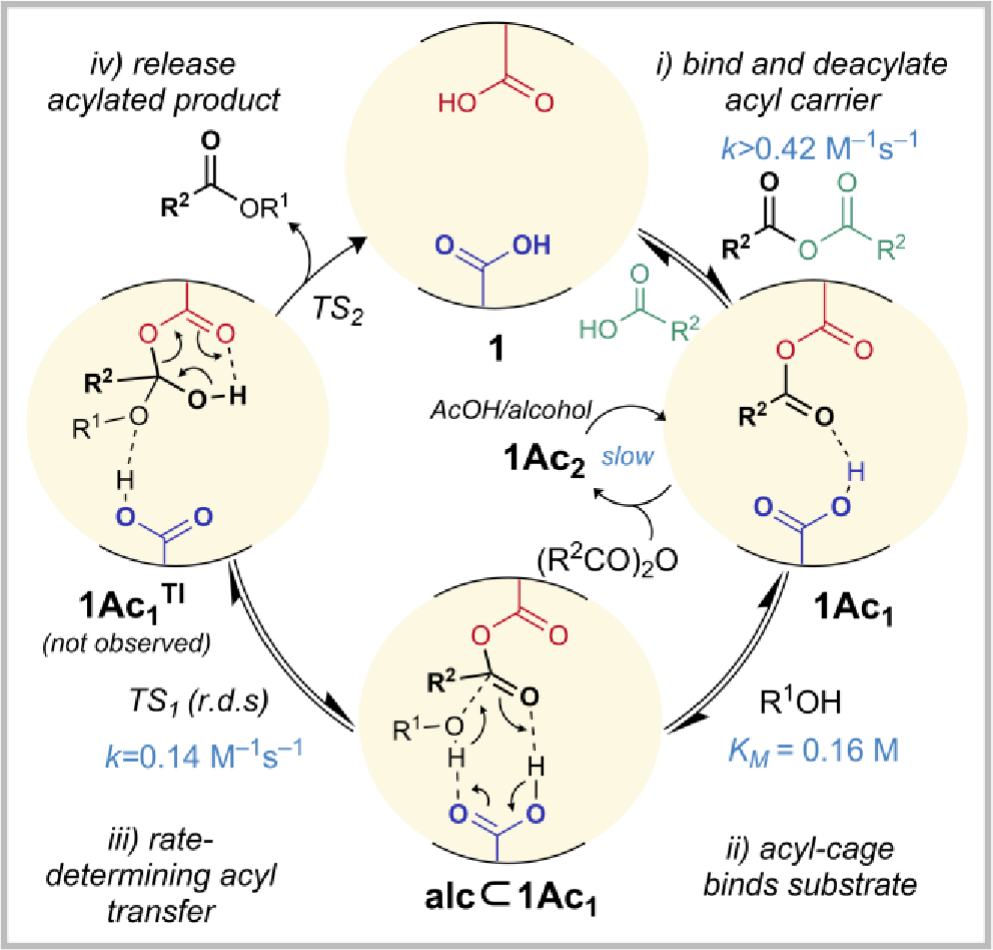
Proposed mechanism of the cage **1**-catalyzed esterification of alcohols with an acyl anhydride acyl carrier (kinetic data shown for Ac_2_O and alcohol **6**).

The proposed overall mechanism for catalysis is shown in Figure 8. On the basis of differential acylation rates of cages (**1** > **3** ≫ **5**) to form **1Ac**_**1**_, **3Ac**_**1**_, and **5Ac**_**1**_, (Figures S26–S28), we propose that initial monoacylation of cage **1** to form **1Ac**_**1**_ is accelerated by the second carboxylic acid group, which likely orients or activates the anhydride reagent by hydrogen bonding. Cavity-based activation of a metastable acyl carrier is proposed to occur in acyl transferases.^88^

Models show an intramolecular hydrogen bond between the remaining carboxylic acid group and the acetyl carbonyl of the anhydride in **1Ac**_**1**_ (Figures 3a and S34), supported by the crystal structure geometry (Figure 3b). Calculations indicate a delicate balance between a longer weaker hydrogen bond and a stronger hydrogen bond that incurs a cage compression strain penalty (Figure S34).

Next, **1Ac**_**1**_ binds the alcohol substrate weakly (*K*_M_ = 0.16 M) to form **alc⊂1Ac**_**1**_ as demonstrated by saturation kinetics and ^1^H NMR titration (Figure 4). Modeling indicates several hydrogen bond interaction modes for the alcohol between the anhydride and acid are possible (Figure S35). Kinetic experiments in the absence of Ac_2_O/AcOH confirm that **1Ac**_**1**_ (rather than bisacylated species **1Ac**_**2**_) is the active acyl transfer species (Figure 3c). **1Ac**_**2**_ forms relatively slowly and can slowly productively reform **1Ac**_**1**_ by the reaction with alcohol or free carboxylic acid (Figure 3c). Eyring analysis confirmed enthalpic stabilization of the cage-catalyzed esterification reaction compared to background, and is consistent with an associative rate-determining step, and perhaps increased entropic preorganization in the cage (Figure 6b). On the basis of differential esterification rates from the activated cages (**1Ac**_**1**_ ≫ **3Ac**_**1**_ = **5Ac**_**1**_ = inactive), we assert that catalysis in cage **1** is contingent on both carboxylic acids. Thus, we propose that in the rate-determining step, the bound alcohol attacks the internal cage anhydride, likely aided by both basic activation of the alcohol nucleophile and protic activation of the anhydride electrophile. Our computational modeling favors a concerted two-proton relay mechanism matching the experimental transition barrier Δ*G*^‡^ in which the carboxylic group acts as a base and an acid, but a proton shuttle mechanism from a species with more zwitterionic character is also plausible, as hypothesized in ribosome acyl transfer mechanisms.^89^ We have not observed competing cage esterification by attack on the hindered anhydride carbonyl group. Finally, ester and catalyst are released; the weak and protic binding mode of the alcohol in the cage means the ester does not inhibit further catalysis (Figure S22).

### Role of Pyridine

HCl inhibits both catalytic steps. Acetic acid, which accumulates over the reaction, inhibits esterification catalysis (Figure S22). Accordingly, free pyridine accelerates both the cage acylation (Figure S28) and esterification steps (Figure S24). This data is consistent with rate acceleration by basic carboxylic functionality; free pyridine likely serves as a buffer to negate acid inhibition of the internal carboxylic groups. In contrast to our original motivation, the cage **1** internal pyridine group basicity is likely masked compared to free pyridine due to interaction with the amide NH donors (but may be enhanced by amide group rotation,^90^Figure S42). Instead, a structural role for pyridine likely dominates: without the pyridyl control over the amide orientation,^77,84^ the ground state of cage **3** has at least 2–3 carbonyl units projected inward,^60,77^ leading to an increased acid–acid distance (**1**: *r*_cc_ = 6.6 Å; **3**: *r*_cc_ = 8.8 Å). Cage **3** therefore incurs a conformational strain cost to access the analogous transition state used by cage **1** (Figure S41). We therefore propose it is the precisely preorganized dicarboxylic acid motif that performs the nucleophilic catalysis, substrate binding, and (bifunctional) protic and basic activation steps that underpin the enzyme-like organocatalysis in cage **1**.

## Conclusions

We have introduced structurally promoted organocatalysis inside a self-assembled organic cage enzyme mimic. Bifunctional hexaamide organic cage **1** promotes acyl transfer reactions from acyl anhydride acyl carriers to alcohol nucleophiles with second-order rate constant enhancements *k*_cage_/*k*_bg_ in CDCl_3_ up to 10^4^ at 298 K. Control experiments with cages **2**–**5** demonstrate catalysis is contingent on the presence of two antipodally arranged carboxylic acid groups and local pyridine units. Catalysis proceeds by formation of a covalent acyl-cage mixed anhydride intermediate featuring an internal intramolecular hydrogen bond, established by modeling, crystallography, and NMR analysis. Substrate saturation kinetics and NMR analysis show weak binding (∼1 kcal/mol) of the alcohol in the cavity interior before nucleophilic attack onto the activated acyl group. Unlike most cavity-promoted reactions, catalysis is contingent on a reaction mode distinct from the background mechanism because the second carboxylic acid organizes the transition state in the cavity. This clear internal catalysis mode, along with the large enthalpic transition state stabilization (despite weak substrate binding), indicates tremendous potential for elaboration of the cage framework to allow highly selective acyl transfer in future iterations. The general mechanism suggests applicability to other condensation reactions, like phosphorylation. The strong sensitivity to acid/base suggests p*K*_a_ tuning may result in large catalytic gains and invites study for insight into electric field effects in cavities and enzymes.

## Supporting Information Available

Synthetic, kinetic and computational procedures and data, spectra, and additional graphs and figures (PDF)
Archive with coordinates and inputs for computational modeling (ZIP)
