# Supplementary material for: Enzyme-like Acyl Transfer Catalysis in a Bifunctional Organic Cage
Source: J Am Chem Soc. 2024 Jun 24;146(26):17887–97. doi: 10.1021/jacs.4c03560 (PMC11228979; doi:10.1021/jacs.4c03560)
Supplement: Supplementary file 1 — ja4c03560_si_001.pdf [file ja4c03560_si_001.pdf]

Supporting information for:

Enzyme-like acyl transfer catalysis in a bifunctional organic cage

Keith G. Andrews,* Tomasz K. Piskorz, Peter N. Horton, Simon J. Coles

Contents

General Methods 5

Synthetic Methods 6

Synthesis of bisaldehyde edge pieces 6

Synthesis of functionalized triptycenes 8

Figure S1. Synthesis of triptycenes **s5** and **s6**. 8

Figure S2. Synthesis of cages **1** and **2**. 9

Figure S3. Synthesis of diacid non-pyridine cage **3** 10

Figure S4. Synthesis of non-acid hexapyridine cage **4** 10

Figure S5. Synthesis of monoacid hexapyridine cage **5** 11

Figure S6. Recycling GPC trace showing separation of the three cage sizes (cage **5**). 13

Mechanistic Experiments 15

Demonstration of acyl transfer catalysis by cage **1** 15

Figure S7. Cage **1** promotes catalysis relative to background and control cages. 15

Estimation of background contributions using measured rate constants 16

Table S1. Background rate constants: 16

Substrate scope for acyl transfer catalysis with cage **1**. 18

Table S2. Relative initial rates and effective second order rate constants for background and cage-promoted catalysis at 298 K. 18

Figure S8. SAR initial rates data for different alcohols and anhydrides 19

Figure S9. Full catalysis profiles. 20

Figure S10. Reaction of cage **1** with different anhydride cofactors 21

Crystal methods 23

Figure S11. Overlaid images of crystal structures **2** and **1Ac$_1$**. 23

Conversion of **1Ac$_1$** and **1Ac$_2$** to ester in isolation 24

Figure S12. 1Ac$_1$ is the active acylating agent. 24

Figure S13. Consumption of **1Ac$_1$** and **1Ac$_2$**. 25

Figure S14. Ester formation is first order in **1Ac$_1$** 25

Figure S15. First order consumption of **1Ac$_2$** 26

Acyl exchange reactions of cage **1** 27

Figure S16. Anhydride/acid scrambling rates with/without 1 mol% cage **1**. 27

Figure S17. Cage speciation kinetics during the anhydride exchange reaction 28

Figure S18. ^{1}H-NMR stack showing equilibrium of acetic anhydride with propionic acid in the presence of cage **1**. 29

Figure S19. ^{1}H-NMR stack showing reaction of acetic anhydride with propionic acid in the absence of cage (background). 30

Michaelis-Menten analysis of cage **1** with acetic anhydride and 2-ethylhexanol 31

Table S3. Initial esterification rate dependency on substrate (alcohol) concentration at constant cage concentration. 31

Figure S20. Michaelis-Menten fit of data in **Table S3**. 32
Notes on the Michaelis-Menten analysis....32
Figure S21. Alcohol binding in the cavity is not strongly affected by AcOH concentration. 33
The effect of additives on esterification catalysis with cages 34
Figure S22. Addition of 50 mol% AcOH to cage **1** promoted esterifications inhibits the rate of catalysis. ..34
Figure S23. No transesterification of ester product is observed in the presence of cage **1**....35
Figure S24. Pyridine enhances the catalytic properties of cage **1** in esterification reactions 36
Figure S25. Addition of 50 mol% pyridine to inactive cage **3** leads to an active catalyst system, which is less susceptible to acid-inhibition. 37
Reaction of cages **1,3,5** with acetic anhydride 38
Figure S26. Cage **5** + Ac_2O. 38
Figure S27. Cage **3** + Ac_2O. 39
Figure S28. Cage **3** + Ac_2O + pyridine (6 equiv wrt cage). 39
Direct comparison of esterification catalysis rate using cage **1** or [cage **3** + 6 equiv pyr] 40
Figure S29. Comparison of initial rates of esterification catalyzed by cage **1**, cage **5**, and cage **3**+pyr under standardized conditions. 40
Eyring Analysis.... 41
Table S4. Second order rate constants for the background esterification reaction at different temperatures. 41
Figure S30. Eyring plot for the background catalysis reaction at 293, 298, 303, 308 and 313 K. 41
Table S5. Second order rate constants for the cage-catalyzed esterification reaction at 293, 298, 303, 308, 313 K. 42
Figure S31. Eyring plot for the cage-catalyzed esterification reaction at 293, 298, 303, 308 and 313 K. ..43
Figure S32. Eyring analysis summary. 43
Estimation of transition state thermodynamic parameters from Eyring analysis 44
Computational Modelling....45
Molecular dynamics....45
Density functional theory (DFT) 45
Conformational Study....45
MD calculations 45
QM calculations....45
Figure S33. Landscape of available conformational space for cage **1**. 46
Table S6. Single point energies of the optimized cage **1** conformers. 46
Table S7. Boltzmann weighted (including degeneracy) populations for the conformers of cage **1**....47
Acetylation of the cage 48
Figure S34. Cage acetylation calculations. 48
Table S8. Single point energies and thermal contributions for acylation reaction of cage **1** into **1Ac$_1$** and **1Ac$_2$**....48
Binding of alcohol in cage **1** and cage **1Ac$_1$** 49

Figure S35. Alcohol binding in cage **1** and **1Ac₁**.......49
Table S9. Single point energies and thermal contributions for alcohol binding in cage **1Ac₁**.......50
Uncatalyzed (background) esterification reaction.......51
Figure S36. QM calculations of the uncatalyzed esterification.......51
Table S10. Single point energies and thermal contributions for the uncatalyzed (background) reaction. ..51
Table S11. Validation of level of theory.......51
Catalyzed esterification reaction.......52
Figure S37. Proposed mechanism (i).......52
Figure S38. Proposed mechanism (ii).......53
Figure S39. Proposed mechanism (iii).......54
Table S12. Single point energies and thermal contributions for the three proposed catalyzed mechanisms. 55
Figure S40. Transition states analyzed using D/I framework.......56
Table S13. Single point energies for Distortion-Interaction (D/I) analysis.......56
Figure S41. Calculated structures and energies for the esterification reaction catalyzed by cage **3**.......57
Table S14. Single point energies and thermal contributions for mechanism (iii) for cage **3**. 57
Figure S42. Amide group rotation and pyridine basicity in cage **1**.......58
Supporting Information References.......59

General Methods

Commercially available reagents were used as received unless stated otherwise. 2-Ethylhexanol and isopropanol were stored over activated 3 Å molecular sieves for 48 h before use. $CDCl_3$ was filtered through alumina and stored over activated 3 Å molecular sieves and granular K_2CO_3 in the dark to remove HCl. Failure to remove HCl affected the rate of catalysis. Activation of molecular sieves involved flame-drying a 50 mL flask of sieves under cycles of vacuum, argon, vacuum for 10 minutes, and 30 minutes cooling. Dry solvents (THF (tetrahydrofuran), CH_2Cl_2, benzene, diethyl ether, DMF (dimethylformamide), pentane) for reactions were purified by a MBraun MB-SPS-5 bench-top SPS system under nitrogen (H_2O content < 20 ppm). All other solvents used were HPLC grade and dried over appropriate drying agents when required. Petroleum ether (petrol) had a boiling point range of 40–60 °C. TFA = trifluoroacetic acid. BuLi = *n*-butyl lithium. All solutions used during workups ($NaHCO_3$, brine) were saturated aqueous solutions, unless otherwise specified. Reactions, unless otherwise stated, were carried out in undried glassware under an air atmosphere. Thin layer chromatography (TLC) was carried out on aluminium-backed silica gel plates with 0.2 mm thick silica gel 60 F254 (Merck) and visualized by UV irradiation at either 254 nm or 366 nm. Preparative flash column chromatography was either carried out using flash silica gel 60 (230-400 mesh) obtained from Sigma-Aldrich, or on a Biotage Isolera One with a 200–400 nm UV detector using *sfar* or KP-sil prepacked columns ("flash cartridges"). Size exclusion chromatography (SEC) was carried out using Bio-Beads S-X3, 40-80 µm (Bio Rad). Evaporation of solvents was performed at 20–50 °C and 5–1010 mbar. Reported yields refer to pure compounds dried under high vacuum (< 0.1 mbar). ^1H and ^{13}C nuclear magnetic resonance (NMR) spectra were recorded on Bruker AVIII HD 400, Bruker NEO 600, Bruker AVIII HD 500, Bruker AVIII HD 600 (Prodigy N2 broadband cryoprobe) spectrometers at 400 MHz, 600 MHz, 500 MHz, and 500 MHz (^1H) and 101 MHz, 151 MHz, 126 MHz and 126 MHz (^{13}C), respectively at 298 K unless stated otherwise. NMR chemical shifts were reported in ppm relative to $SiMe_4$ (δ = 0) and were referenced internally with respect to residual solvent protons using the reported values. All chemical shifts are reported in ppm, coupling constants are reported in Hz and ^1H multiplicities are reported in accordance with the following: app= apparent; s = singlet; br = broad; d = doublet; t = triplet; q = quartet; and m = multiplet. ^1H assignments were made using 2D NMR methods (COSY, NOESY, HSQC, HMBC). Electrospray mass spectrometry was carried out on a Waters Micromass LCT Premier XE spectrometer using 90:10 MeOH:H_2O (+0.1% formic acid) as the mobile phase. High-resolution mass spectrometry (HR-MS) measurements were performed by the mass spectrometry service at the University of Oxford on a Waters GTC classic. MALDI measurements were performed using a Bruker Autoflex Speed MALDI-ToF using DCTB as the matrix (*trans*-2-[3-(4-tert-butylphenyl)-2-methyl-2-propenylidene]malononitrile, CAS=300364-84-5). Acronym wrt = with respect to.

Synthetic Methods

Synthesis of bisaldehyde edge pieces

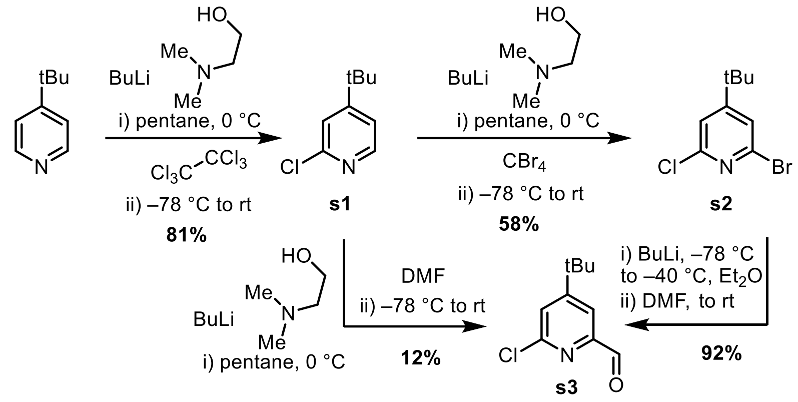

4-(tert-butyl)-2-chloropyridine s1: Using an adapted literature procedure,[1] in a flame-dried flask under argon, dry 2-(dimethylamino)ethan-1-ol (1.43 g, 1.61 mL, 2 eq, 16.0 mmol) was dissolved in pentane (50 mL), cooled to 0 °C, and added dropwise was *n*-butyllithium (1.6 M in hexanes) (2.050 g, 20.00 mL, 1.6 molar, 4 eq, 32.00 mmol) over 5 min. The reaction was stirred at 0 °C for 15 min and then added was a solution of 4-(tert-butyl)pyridine (1.08 g, 1.17 mL, 1 eq, 8.00 mmol) in pentane (10 mL), dropwise over 2 min. After stirring for 1 h at 0 °C, the reaction was cooled to –78 °C and added was perchloroethane (4.734 g, 2.5 eq, 20.00 mmol) as a solution in THF (20 mL) and the reaction stirred for 1 h at –78 °C. The reaction was allowed to warm to 0 °C over 10 min and then quenched with water (30 mL), extracted with diethyl ether (x2) and dried over magnesium sulfate. After filtration, the reaction was concentrated to a residue, the solid insoluble in petrol discarded, and the liquid purified by flash column chromatography (biotage, 25 g, loaded in petrol, 0-5% EtOAc/petrol). The desired product **s1** eluted as an orange oil (1.10 g, 81%) with data consistent with the literature:[2] **^{1}H NMR** (400 MHz, $CDCl_3$) δ 8.31 – 8.26 (m, 1H), 7.31 – 7.28 (m, 1H), 7.20 (dd, J = 5.3, 1.7 Hz, 1H), 1.31 (s, 9H); **^{13}C NMR** (101 MHz, $CDCl_3$) δ 163.7, 151.9, 149.5, 121.4, 119.8, 35.1, 30.5; **MS** ESI(+): 170, 172.

Conversion of pyridine s1 to s3 via s2

2-bromo-4-(tert-butyl)-6-chloropyridine s2: Using an adapted literature procedure,[1] in a flame-dried flask under argon, dry 2-(dimethylamino)ethan-1-ol (2.67 g, 3.01 mL, 2 eq, 29.9 mmol) in pentane (90 mL) was cooled to 0 °C and added dropwise was *n*-butyllithium (1.6 M in hexanes) (3.84 g, 37.4 mL, 1.6 molar, 4 eq, 59.9 mmol) over 5 min. The reaction was stirred at 15 min for 0 °C and then added was a solution of 4-(tert-butyl)-2-chloropyridine (2.54 g, 1 eq, 15.0 mmol) in pentane (36 mL), dropwise over 2 min. After stirring for 0 °C at 1 h, the reaction was cooled to –78 °C and added was carbon tetrabromide (12.4 g, 2.5 eq, 37.4 mmol) as a solution in THF (36 mL) and the reaction stirred for 1 h. The reaction was allowed to warm to 0 °C over 10 min and then quenched with water (60 mL), extracted with diethyl ether (x2) and dried over magnesium sulfate. After filtration, the reaction was concentrated to a residue, the solid insoluble in petrol/dichloromethane (1:1) discarded, and the liquid purified by flash column chromatography (biotage, 25 g, loaded in petrol/dichloromethane, 0-50% EtOAc/petrol). The desired product **s2** eluted as a brown solid (2.16 g, 58%) with data: **^{1}H NMR (400 MHz, $CDCl_3$)** δ 7.38 (d, J = 1.4 Hz, 1H), 7.26 (d, J = 1.4 Hz, 1H), 1.30 (s, 9H); **^{13}C NMR (101 MHz, $CDCl_3$)** δ 166.1, 150.8, 140.9, 124.2, 120.7, 35.5, 30.5; **HR-MS m/z** (ESI+): 247.9847 ($C_9H_{12}BrClN$, MH+ requires 247.9836).

4-(tert-butyl)-6-chloropicolinaldehyde s3: Using an adapted literature procedure,[1] in a flame-dried flask under argon, 2-bromo-4-(tert-butyl)-6-chloropyridine (1.50 g, 1 Eq, 6.04 mmol) was dissolved in diethyl ether (20 mL) and cooled to –78 °C. *n*-Butyllithium (1.6 M in hexanes) (425 mg, 4.15 mL, 1.6 molar, 1.1 eq, 6.64 mmol) was added dropwise (the solution became dark immediately), and the was reaction stirred at –78 °C for 30 min. After this time, the reaction was warmed to –30 °C and added was *N,N*-dimethylformamide (662 mg, 701 µL, 1.5 Eq, 9.05 mmol), maintaining –30 °C. The reaction was warmed to 0 °C over 10 min. The reaction was quenched with saturated aqueous ammonium chloride solution and extracted with diethyl ether, washed with brine x 4, dried over magnesium sulfate, filtered and concentrated under reduced pressure. The residue was purified by flash column chromatography to give 4-(tert-butyl)-6-chloropicolinaldehyde **s3** (1.1 g, 5.6 mmol, 92 %) as a pale yellow oil. **Data matches s3, below.**

Direct reaction of pyridine s1 to form s3

4-(tert-butyl)-6-chloropicolinaldehyde s3: Using an adapted literature procedure,[1] in a flame-dried flask under argon, dry 2-(dimethylamino)ethan-1-ol (946 mg, 1.07 mL, 2 eq, 10.6 mmol) in pentane (30 mL) was cooled to 0 °C and added dropwise was *n*-butyllithium (1.6 M in hexanes) (1.359 g, 13.26 mL, 1.6 molar, 4 eq, 21.22 mmol) over 5 min. The reaction was stirred at 15 min for 0 °C and then added was a solution of 4-(tert-butyl)-2-chloropyridine (0.900 g, 1 eq, 5.31 mmol) in pentane (12 mL), dropwise over 2 min. After stirring for 0 °C at 1 h, the reaction was cooled to –78 °C and added was *N,N*-dimethylformamide (970 mg, 1.03 mL, 2.5 eq, 13.3 mmol) as a solution in THF (12 mL) and the reaction stirred for 1 h. The reaction was allowed to warm to 0 °C over 10 min and then quenched with water (20 mL), extracted with diethyl ether (x2) and dried over magnesium sulfate. After filtration, the solution was concentrated to a residue, and purified by flash column chromatography, (biotage, 25 g, loaded in petrol/dichloromethane, 0-5% EtOAc/petrol). The product was not clean, and was repurified by chromatography (biotage, 25 g, loaded in petrol/dichloromethane, 0-50% CH_2Cl_2/petrol) to give the desired product **s3** as a pale yellow oil (126 mg, 12%): **^{1}H NMR (400 MHz, $CDCl_3$)** δ 9.98 (s, 1H), 7.88 (d, *J* = 1.6 Hz, 1H), 7.52 (d, *J* = 1.6 Hz, 1H), 1.34 (s, 9H); **^{13}C NMR (101 MHz, $CDCl_3$)** δ 192.3, 165.2, 152.9, 152.4, 126.0, 117.7, 35.6, 30.5; **HR-MS m/z** (ESI+): 198.0681 ($C_{10}H_{13}NCl$, MH+ requires 198.0680).

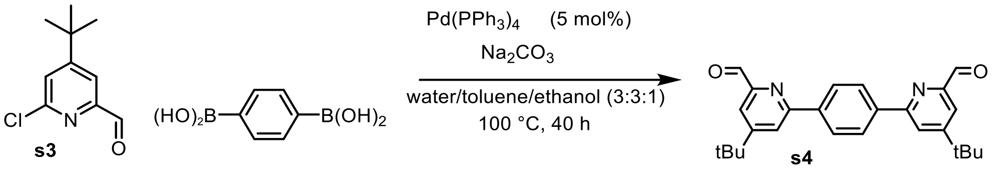

6,6'-(1,4-phenylene)bis(4-(tert-butyl)picolinaldehyde) s4: In a flame-dried flask under argon, 4-(tert-butyl)-6-chloropicolinaldehyde (477.0 mg, 2 Eq, 2.413 mmol), and tetrakis(triphenylphosphine)palladium(0) (86.0 mg, 0.0617 eq, 74.4 µmol) were dissolved in toluene (9 mL) and ethanol (3 mL) and the mixture briefly degassed (three vacuum/argon cycles). Added was a solution of sodium carbonate (447.6 mg, 3.5 eq, 4.223 mmol) in water (9 mL), and the reaction degassed once more. The mixture was then stirred vigorously at 100 °C for 40 h. The reaction mixture was cooled, diluted with ethyl acetate, and washed with sodium hydroxide and brine and dried over magnesium sulfate, filtered and concentrated. The residue was purified by flash column chromatography, eluting with 0-20% EtOAc/petrol (holding at 14%) to give **s4** as a pale yellow solid (236 mg, 49%); **^{1}H NMR (400 MHz, $CDCl_3$)** δ 10.21 (s, 2H), 8.24 (s, 4H), 8.01 (d, *J* = 1.7 Hz, 2H), 7.97 (d, *J* = 1.7 Hz, 2H), 1.44 (s, 18H); **^{13}C NMR (101 MHz, $CDCl_3$)** δ 194.5, 162.6, 157.5, 153.1, 139.8, 127.7, 121.9, 117.6, 35.5, 30.7; **HR-MS m/z** (ESI+): 401.2222 ($C_{26}H_{29}O_2N_2$, MH+ requires 401.2224).

Synthesis of functionalized triptycenes

Triptycenes **s5**[3] and **s6**[4,5] were prepared as previously reported.[6]

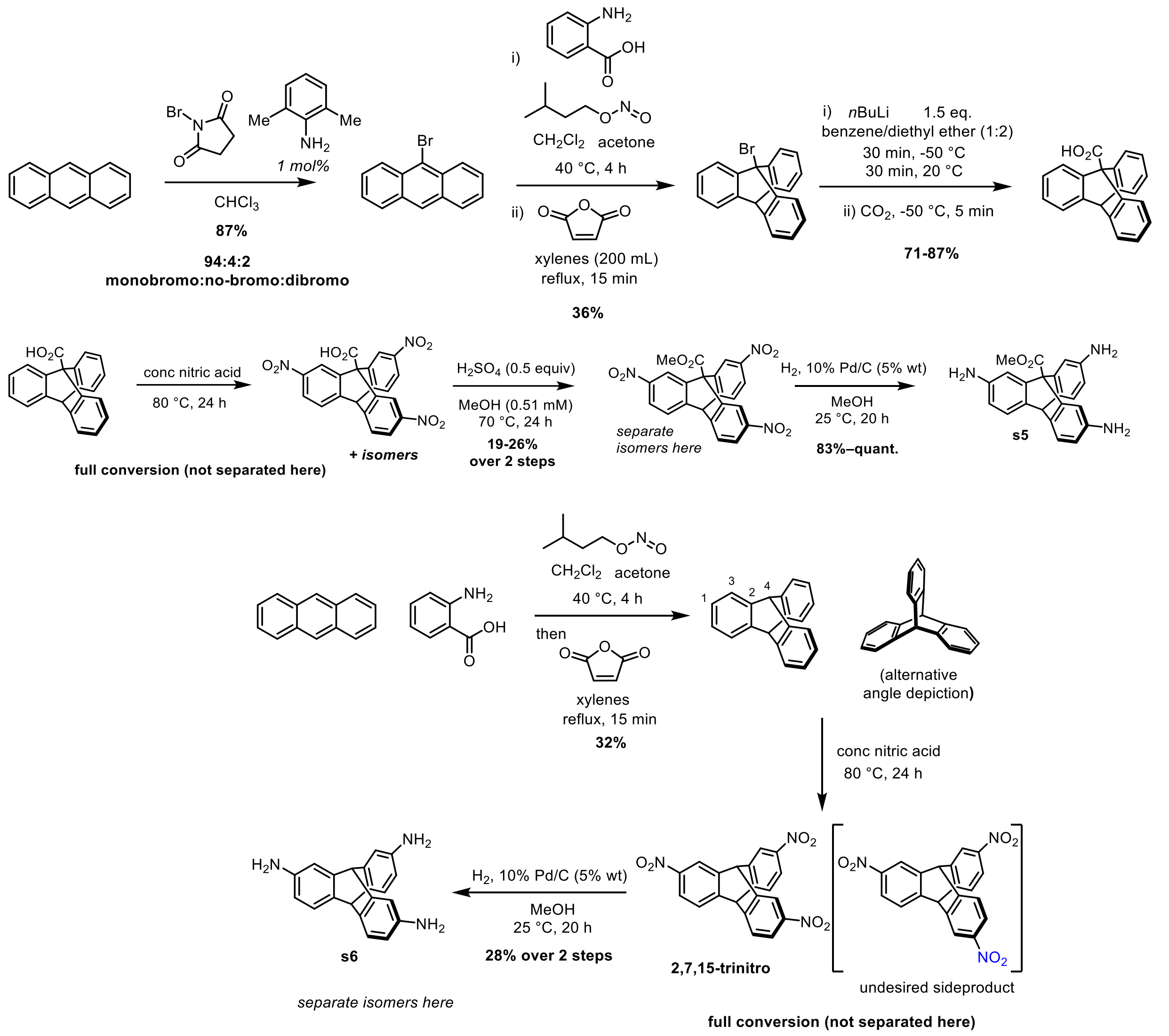

Figure S1. Synthesis of triptycenes **s5** and **s6**.

Figure S2. Synthesis of cages **1** and **2**

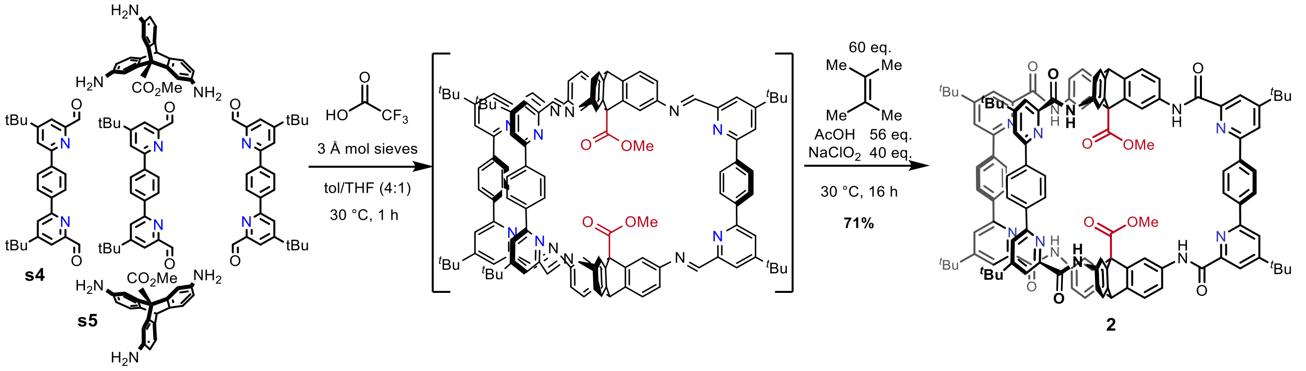

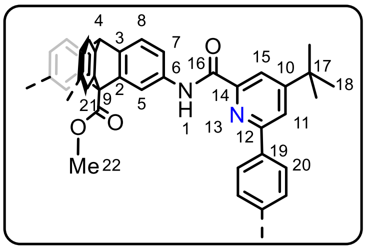

Dimethyl ester hexapyridine hexaamide cage 2: Triptycene **s5** (100 mg, 280 µmol) was dissolved in THF (50 mL) in a flask containing 3 Å molecular sieves (0.15 g). The solution was diluted with toluene (100 mL) containing trifluoroacetic acid (10 µL, 131 µmol). Separately, bisaldehyde **s4** (168 mg, 420 µmol) was dissolved in toluene (100 mL, no trifluoroacetic acid present) and this solution added to the reaction over 60 seconds. After stirring for 1 h at ambient temperature, MALDI-ToF analysis indicated high conversion to the desired hexaimine cage. Added was 2,3-dimethyl-2-butene (998 µL, 8.39 mmol, 60 eq), sodium chlorite (80% w/w, 633 mg, 5.60 mmol, 40 eq) and glacial acetic acid (448 µL, 7.83 mmol, 56 eq). The reaction was stirred vigorously in the dark at 30 °C for 16 h. The reaction was filtered to remove the solids, diluted with ethyl acetate (200 mL), and washed with water and brine, and the resulting organics dried over magnesium sulfate, filtered and concentrated. The resulting solid was triturated from THF to give **2** as a white powder (189 mg, 71%) with data: **^{1}H NMR (600 MHz, $CDCl_3$)** δ 9.90 (s, $6H^{1}$), 8.32 (d, J = 1.7 Hz, $6H^{11}$), 8.06 (s, $12H^{20}$), 7.98 (dd, J = 8.1, 1.9 Hz, $6H^{7}$), 7.84 (d, J = 1.7 Hz, $6H^{15}$), 7.51 (d, J = 8.1 Hz, $6H^{8}$), 7.50 (d, J = 1.9 Hz, $6H^{5}$), 5.47 (s, $2H^{4}$), 4.62 (s, $6H^{22}$), 1.41 (s, $54H^{18}$); **^{13}C NMR (151 MHz, $CDCl_3$)** δ 170.6 (C^{21}), 163.1 (C^{10}), 162.8 (C^{16}), 156.7 (C^{12}), 150.0 (C^{14}), 143.3 ($C^{3/2}$), 142.2 ($C^{3/2}$), 140.5 (C^{6}), 134.3 (C^{19}), 128.0 (C^{20}), 124.3 (C^{8}), 121.4 (C^{15}), 120.0 (C^{7}), 118.9 (C^{11}), 117.0 (C^{5}), 61.8 (C^{9}), 53.2 (C^{4}), 52.7 (C^{22}), 35.5 (C^{17}), 30.7 (C^{18}); **MS *m/z*** (MALDI-ToF-RP) (DCTB matrix) 1903.5 ($C_{122}H_{111}N_{12}O_{10}$, $[M+H]^{+}$ requires 1903.9).

The oxidation step can also be performed with NaH_2PO_4 (56 eq) instead of AcOH (56 eq) as the acid, but the reaction tends to take ~10 days.[6]

Note: $NaClO_2$ came as a white solid, 80% pure wt/wt. 633 mg × 0.80 = 506.4 mg = 5.60 mmol = 40 equiv relative to 0.14 µmol maximum yield of cage.

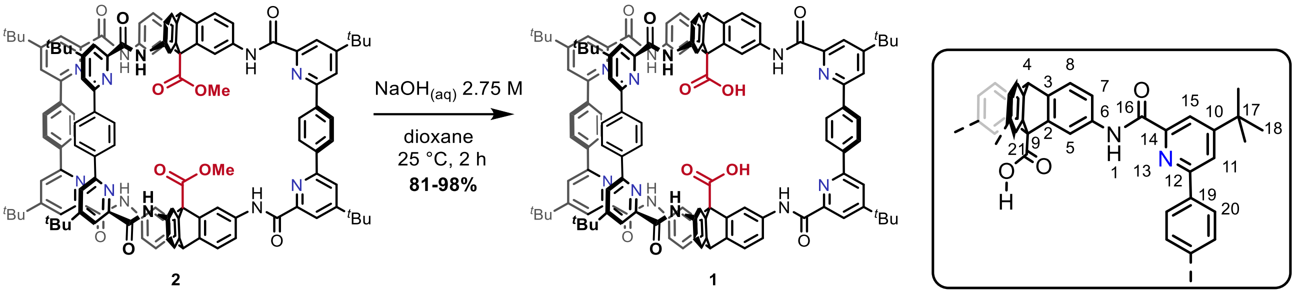

Diacid hexapyridine cage 1: Dimethyl ester cage **2** (150 mg, 78.8 µmol) was suspended in dioxane (10 mL) and added an aqueous sodium hydroxide solution (110 mg, 1.0 mL, 2.75 M, 34.9 eq, 2.75 mmol). The reaction was followed by TLC until complete (~2-10 h) and then cautiously quenched to neutrality with dilute HCl. The mixture was extracted with ethyl acetate, and the organics washed with water and dried over magnesium sulfate, filtered and concentrated under reduced pressure. The resulting solid was triturated in pentane, and the desired cage **1** was collected by filtration and vacuum-dried at 55 °C, 0.3 mbar to give a white powder (120 mg, 81%); **^{1}H NMR (600 MHz, $CDCl_3$)** δ 9.92 (s, 6H^1), 8.30 (d, *J* = 0.9 Hz, 6H^{11}), 8.04 (s, 12H^{20}), 7.95 (dd, *J* = 7.5 Hz, 6H^7), 7.80 (s, 6H^{15}), 7.70 (s, 6H^5), 7.44 (d, *J* = 7.5 Hz, 6H^8), 5.36 (s, 2H^4), 1.39 (s, 54H^{18}); **^{13}C NMR (126 MHz, $CDCl_3$)** 172.7 (C^{21}), 163.2 (C^{10}), 162.9 (C^{16}), 156.6 (C^{12}), 149.9 (C^{14}), 143.1 ($C^{3/2}$), 142.2 ($C^{3/2}$), 140.4 (C^6), 134.2 (C^{19}), 128.0 (C^{20}), 124.2 (C^8), 121.2 (C^{15}), 119.8 (C^7), 118.8 (C^{11}), 117.2 (C^5), 61.8 (C^9), 53.3 (C^4), 35.5 (C^{17}), 30.7 (C^{18}); **MS *m/z*** (MALDI-ToF-RP) (DCTB matrix) 1875.5 ($C_{120}H_{107}N_{12}O_{10}$, $[M+H]^+$ requires 1875.8).

Figure S3. Synthesis of diacid non-pyridine cage **3**

As previously reported.[6]

Figure S4. Synthesis of non-acid hexapyridine cage **4**

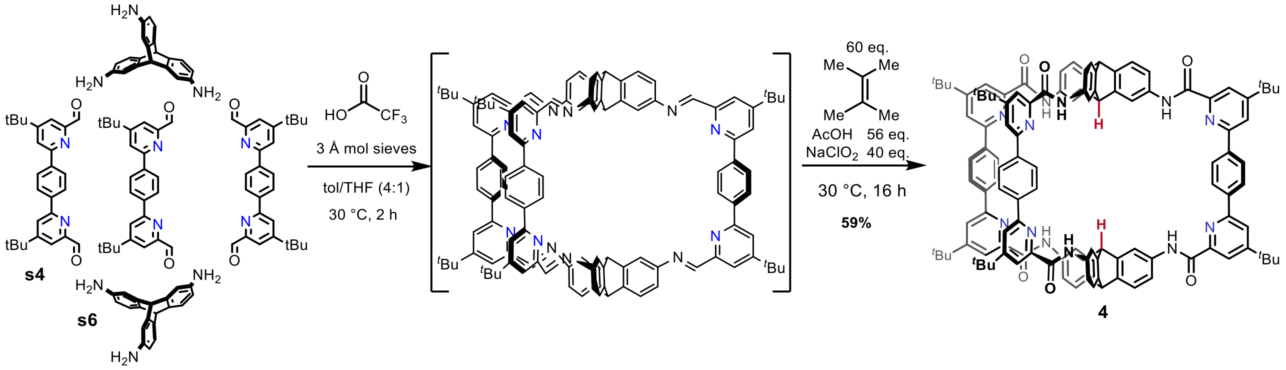

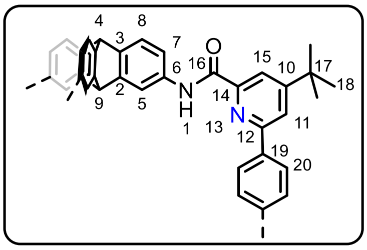

Synthesis of cage **4** was performed as for cage **2**, except with triptycene **s6** (9,10-dihydro-9,10-[1,2]benzenoanthracene-2,7,15-triamine, 15.0 mg, 50.1 µmol) and 6,6'-(1,4-phenylene)bis(4-(tert-butyl)picolinaldehyde) (30.1 mg, 75.2 µmol) in toluene 30 mL and THF 7.5 mL, scaling all other reagents appropriately.

Cage **4** isolated as a white solid after trituration with THF (26.4 mg, 59%) with data: **^{1}H NMR (600 MHz, $CDCl_3$)** δ 9.89 (s, 6H^1), 8.31 (d, *J* = 1.6 Hz, 6H^{11}), 8.05 (s, 12H^{20}), 7.89 (dd, *J* = 8.1, 1.8 Hz, 6H^7), 7.83 (d, *J* = 1.6 Hz, 6H^{15}), 7.49 (d, *J* = 8.1 Hz, 6H^8), 7.19 (d, *J* = 1.8 Hz, 6H^5), 5.51 (s, 2H^4), 5.41 (s, 2H^9), 1.41 (s, 54H^{18}); **^{13}C NMR (151 MHz, $CDCl_3$)** δ 163.2 (C^{10}), 162.7 (C^{16}), 156.7 (C^{12}), 150.0 (C^{14}), 145.3 (C^3), 142.0 (C^2), 140.5 (C^6), 134.2 (C^{19}), 128.02 (C^{20}), 124.4 (C^8), 121.3 (C^{15}), 119.5 (C^7), 118.8 (C^{11}), 116.7 (C^5), 54.1 (C^9), 52.8 (C^4), 35.5 (C^{17}), 30.7 (C^{18}); **MS *m/z*** (MALDI-ToF-RP) (DCTB matrix) 1787.5 ($C_{118}H_{107}N_{12}O_6$, $[M+H]^+$ requires 1787.8).

Figure S5. Synthesis of monoacid hexapyridine cage **5**

Triptycene **s7**

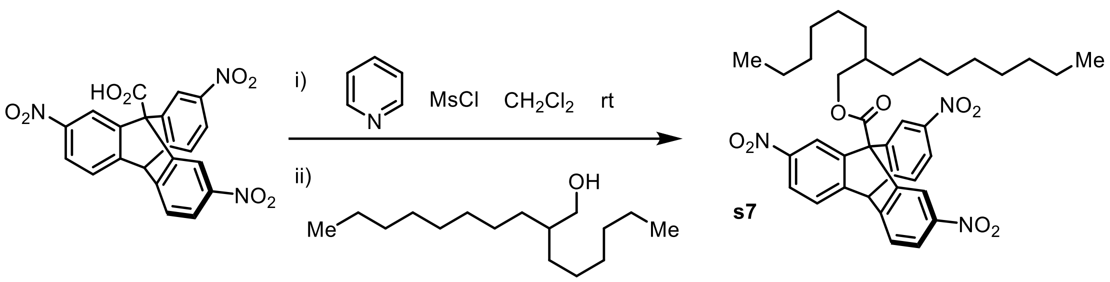

Triptycene s7 (2-hexyldecyl 2,7,15-trinitro-9,10-[1,2]benzenoanthracene-9(10H)-carboxylate): According to an adapted literature procedure,[3,7] 2,7,15-trinitro-9,10-[1,2]benzenoanthracene-9(10H)-carboxylic acid (20.0 mg, 1 eq, 46.2 μmol) was dissolved in dichloromethane (2.5 mL) and added was pyridine (102 mg, 105 μL, 28 eq, 1.29 mmol) followed by methanesulfonyl chloride (26.4 mg, 17.9 μL, 5 eq, 231 μmol). After 1 h stirring at room temperature, added was 2-hexyldecan-1-ol (67.1 mg, 80.3 μL, 6 eq, 277 μmol), and the reaction stirred at 20 °C for 16 h. After this time, the reaction was diluted with ethyl acetate, and washed with saturated aqueous sodium bicarbonate, then brine (containing 5% 3 M HCl v/v), then water. The organics were dried over magnesium sulfate, filtered, and concentrated. The crude material was purified (from alcohol starting material and the dimerized ether of the alcohol) by flash column chromatography (0-100% diethyl ether in petrol, then 100% dichloromethane, which sometimes washed further desired from the silica.) The desired **s7** was isolated as a white solid (24.2 mg, 80%) with data: **^{1}H NMR (400 MHz, $CDCl_3$)** δ 8.68 (d, *J* = 2.1 Hz, 3H), 8.08 (dd, *J* = 8.2, 2.2 Hz, 3H), 7.64 (d, *J* = 8.2 Hz, 3H), 5.77 (s, 1H), 4.79 (d, *J* = 5.1 Hz, 2H), 2.03 – 1.91 (m, 1H), 1.54 – 1.36 (m, 8H), 1.35 – 1.14 (m, 16H), 0.90 – 0.79 (m, 6H); **^{13}C NMR (101 MHz, $CDCl_3$)** δ 167.95, 149.14, 146.38, 143.41, 125.00, 122.94, 120.29, 70.50, 61.61, 53.91, 37.61, 32.00, 31.91, 31.56 (2C), 29.90, 29.66, 29.56, 29.38, 27.02, 26.98, 22.78, 22.72, 14.24, 14.20; **MS m/z** (ESI+): 680.5 ($C_{37}H_{43}N_3O_8Na$, [M+Na]+ requires 680.3).

Triptycene s8

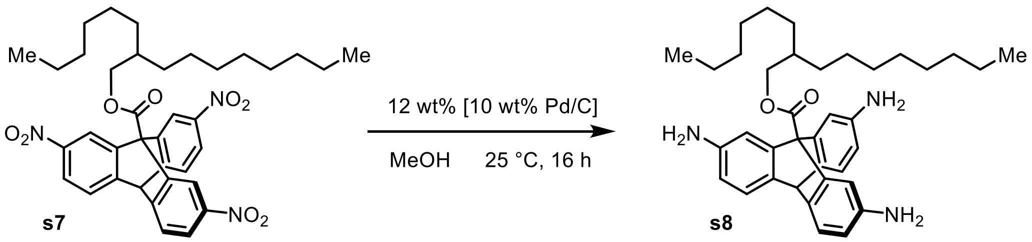

Triptycene s8: Triptycene **s7** (24.2 mg, 1 eq, 36.8 μmol) was suspended in methanol (5 mL) under an argon atmosphere and added was 10% wt Pd/C (3 mg, 12% wt loading wrt triptycene) and the argon atmosphere replaced with hydrogen. The reaction was stirred vigorously under a hydrogen atmosphere (double balloon) for 16 h, and then filtered carefully through Celite under nitrogen, ensuring the palladium did not become dry. (The Celite/Pd was quenched with water). The organic solution was concentrated and purified by trituration in petrol and filtration of the solid to give **s8** as a white solid (20.0 mg, 96%) with data: **^{1}H NMR (400 MHz, $CDCl_3$)** δ 7.06 (d, *J* = 2.2 Hz, 3H), 7.06 (d, *J* = 7.8 Hz, 3H), 6.30 (dd, *J* = 7.8, 2.2 Hz, 3H), 5.01 (s, 1H), 4.54 (d, *J* = 5.2 Hz, 2H), 3.59 – 3.28 (s, 6H),

1.97 – 1.83 (m, 1H), 1.57 – 1.37 (m, 8H), 1.37 – 1.20 (m, 16H), 0.97 – 0.80 (m, 6H); **^{13}C NMR (101 MHz, CDCl$_3$)** δ 170.99, 144.38, 143.33, 137.65, 123.35, 112.27, 111.52, 68.06, 52.03, 37.59, 32.05, 32.02, 31.65 (2C), 30.46, 30.18, 29.84, 29.81, 29.46, 27.09, 27.05, 22.80, 14.25, 14.23; **HR-MS m/z** (ESI+): 568.3897 ($C_{37}H_{50}N_3O_2$, MH+ requires 568.3898).

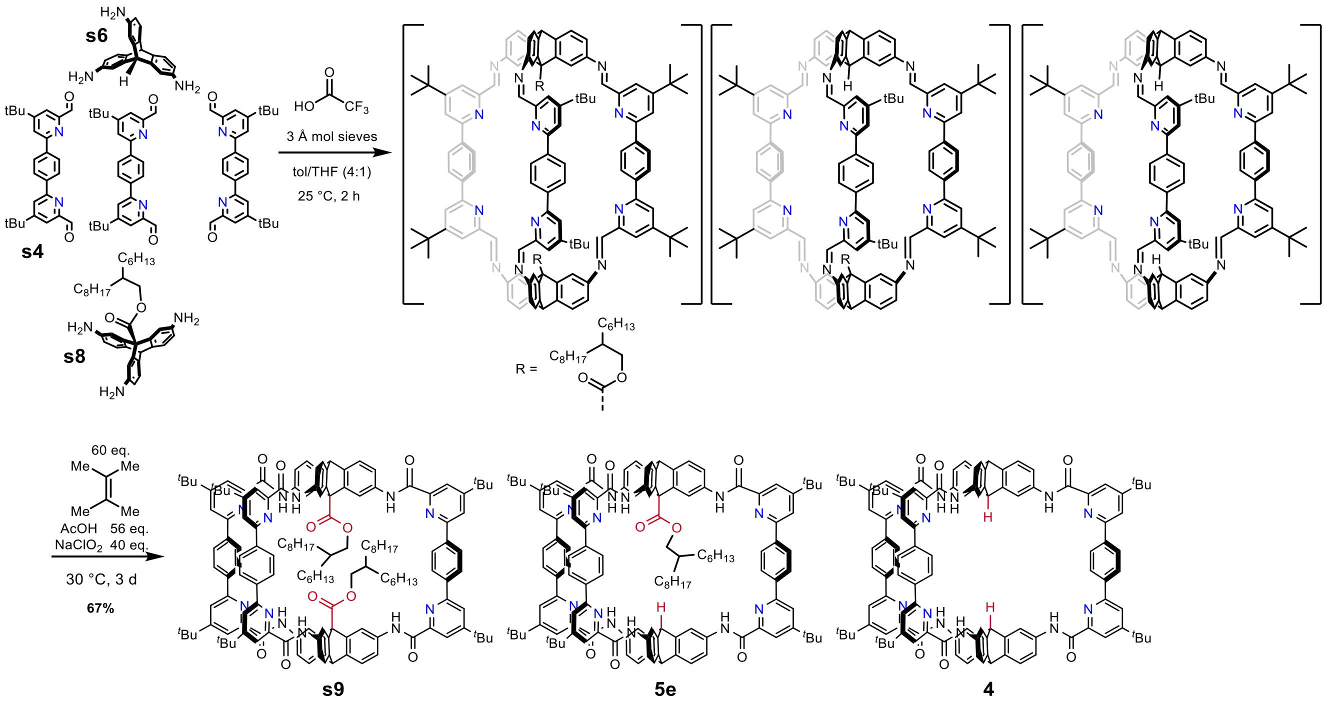

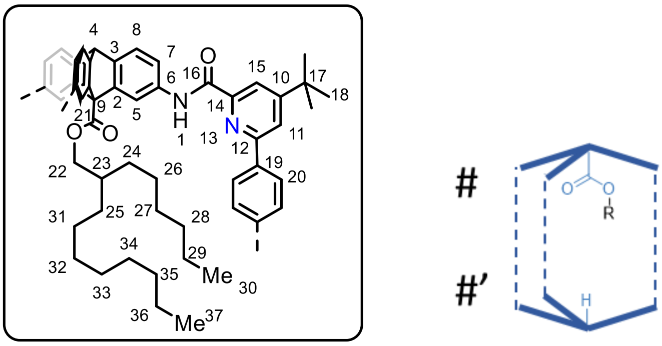

(# and #' = atom numbering)

Cages s9, 5e, 4: Bisaldehyde **s4** (46.6 mg, 0.116 µmol) was dissolved in toluene (24 mL) and added to a dry round-bottom flask containing 3 Å molecular sieves (0.5 g) and further toluene (24 mL) containing 2.5 µL TFA. Added to this stirring solution under argon was a solution of alkyl ester triptycene **s8** (22.2 mg, 0.0391 µmol) and triptycene **s6** (11.7 mg, 0.0391 µmol) in THF (11.7 mL). After 1 h stirring, MALDI showed a stable mixture of cage species, and added was 2,3-dimethylbut-2-ene (280 µL, 60 Eq, 2.35 mmol), acetic acid (56.4 µL, 56 Eq, 2.19 mmol) and sodium chlorite (80% w/w, 176.8 mg, 40 Eq, 1.564 µmol). The reaction was stirred rigorously in the dark at 30 °C for 16 h. The reaction was filtered, diluted in ethyl acetate, and washed with dilute aqueous sodium bicarbonate. The organics were dried over magnesium sulfate, filtered, concentrated, and the residue purified by recycling gel-permeation chromatography (toluene, 1% pyridine) to give three off-white solids corresponding to (in order of elution):

Bisester cage s9: **^{1}H NMR (600 MHz, CDCl$_3$)** δ 9.88 (s, 6H^1), 8.33 (d, J = 1.7 Hz, 6H^{11}), 8.06 (s, 12H^{20}), 7.98 (dd, J = 8.1, 1.8 Hz, 6H^7), 7.86 (d, J = 1.7 Hz, 6H^{15}), 7.55 (br s, 6H^5), 7.50 (d, J = 8.1 Hz, 6H^8), 5.45 (s, 2H^4), 4.96 (d, J = 2.9 Hz, 4H^{22}), 2.15 – 2.07 (m, 2H^{23}), 1.78 – 1.70 (m, 4H$^{24/25}$), 1.66 – 1.55 (m, 4H$^{24/25}$), 1.42 (s, 54H^{18}), 1.44 – 1.33

(m, 12H), 1.13 – 1.02 (m, 8H), 0.82 – 0.73 (m, 12H), 0.72 – 0.60 (m, 12H), 0.49 (t, J = 7.3 Hz, 6H$^{30/37}$), 0.33 (t, J = 7.3 Hz, 6H$^{30/37}$); **^{13}C NMR (151 MHz, CDCl$_3$)** δ 170.3 (C^{21}), 163.1 (C^{10}), 162.8 (C^{16}), 156.5 (C^{12}), 150.0 (C^{14}), 143.7 (C$^{3/2}$), 142.3 (C$^{3/2}$), 140.3 (C^{6}), 134.2 (C^{19}), 127.9 (C^{20}), 124.2 (C^{8}), 121.1 (C^{15}), 119.9 (C^{7}), 118.9 (C^{11}), 117.2 (C^{5}), 69.4 (C^{22}), 61.8 (C^{9}), 53.3 (C^{4}), 35.5 (C^{17}), 32.1 (C$^{24/25}$), 32.0 (C$^{24/25}$), 31.7 (C^{alkyl}), 31.6 (C^{alkyl}), 30.7 (C^{18}), 29.9 (C^{alkyl}), 29.6 (C^{alkyl}), 29.5 (C^{alkyl}), 29.3 (C^{alkyl}), 29.0 (C^{alkyl}), 27.4 (C^{alkyl}), 27.3 (C^{alkyl}), 22.4 (C^{alkyl}), 22.3 (C^{alkyl}), 14.0 (C$^{30/37}$), 13.9 (C$^{30/37}$); **MS *m/z*** (MALDI-ToF-RP) (DCTB matrix) 2345.9 ($C_{152}H_{170}N_{12}O_{10}Na$, [M+Na]$^+$ requires 2346.3).

Monoester cage 5e: **^{1}H NMR (600 MHz, CDCl$_3$)** δ 9.94 (s, 3H$^{1/1'}$), 9.89 (s, 3H$^{1/1'}$), 8.35 (d, J = 1.7 Hz, 3H$^{11/11'}$), 8.34 (d, J = 1.7 Hz, 3H$^{11/11'}$), 8.08 (s, 12H^{20}), 8.00 (dd, J = 8.1, 1.8 Hz, 3H$^{7/7'}$), 7.94 (dd, J = 8.1, 1.9 Hz, 3H$^{7/7'}$), 7.88 (d, J = 1.7 Hz, 3H$^{7/7'}$), 7.87 (d, J = 1.7 Hz, 3H$^{15/15'}$), 7.57 (d, J = 1.6 Hz, 3H$^{15/15'}$), 7.54 (d, J = 8.2 Hz, 3H$^{8/8'}$), 7.52 (d, J = 8.2 Hz, 3H$^{8/8'}$), 7.23 (d, J = 1.9 Hz, 3H$^{15/15'}$), 5.53 (s, 1H$^{4'}$), 5.51 (s, 1H$^{9'}$), 5.50 (s, 1H^{4}), 2.16 – 2.09 (m, 1H^{23}), 1.78 – 1.69 (m, 2H), 1.69 – 1.63 (m, 2H), 1.63 – 1.56 (m, 6H), 1.44 (s, 54H), 1.20 – 1.08 (m, 8H), 0.92 – 0.80 (m, 8H), 0.52 (t, J = 7.3 Hz, 3H$^{30/37}$), 0.39 (t, J = 7.3 Hz, 3H$^{30/37}$); **^{13}C NMR (151 MHz, CDCl$_3$)** δ 171.17 (C^{21}), 163.17 (C$^{10/10'}$), 163.11 (C$^{10/10'}$), 162.79 (C$^{16/16'}$), 162.75 (C$^{16/16'}$), 156.56 (2C$^{12,12'}$), 150.06 (C$^{14/14'}$), 149.93 (C$^{14/14'}$), 145.35 (C$^{3'}$), 143.43 (C^{3}), 142.22 (C$^{2/2'}$), 142.09 (C$^{2/2'}$), 140.37 (C$^{6/6'}$), 140.34 (C$^{6/6'}$), 134.24 (C$^{19/19'}$), 134.22 (C$^{19/19'}$), 127.98 (C$^{20/20'}$), 127.91 (C$^{20/20'}$), 121.25 (2C$^{15,15'}$), 119.92 (C$^{7/7'}$), 119.61 (C$^{7/7'}$), 118.94 (C$^{11/11'}$), 118.84 (C$^{11/11'}$), 117.08 (C$^{5/5'}$), 116.69 (C$^{5/5'}$), 69.17 (C^{22}), 62.05 (C^{9}), 53.94 (C$^{9'}$) 53.19 (C$^{4,4'}$), 52.87 (C$^{4,4'}$), 35.52 (C$^{17/17'}$), 35.51 (C$^{17/17'}$), 32.13 (C$^{24/25}$), 32.11 (C$^{24/25}$), 31.72 (C^{alkyl}), 31.63 (C^{alkyl}), 30.72 (C^{alkyl}), 29.85 (C^{alkyl}), 29.82 (C^{alkyl}), 29.53 (C^{alkyl}), 29.48 (C^{alkyl}), 29.02 (C^{alkyl}), 27.38 (C^{alkyl}), 27.36 (C^{alkyl}), 22.45 (C^{alkyl}), 22.32 (C^{alkyl}), 13.95 (C$^{30/37}$), 13.89 (C$^{30/37}$); **MS *m/z*** (MALDI-ToF-RP) (DCTB matrix) 2055.7 ($C_{134}H_{137}N_{12}O_8$, [M+H]$^+$ requires 2056.1).

Cage 4, with no acid groups, is as reported for pyridine non-acid cage **4** above.

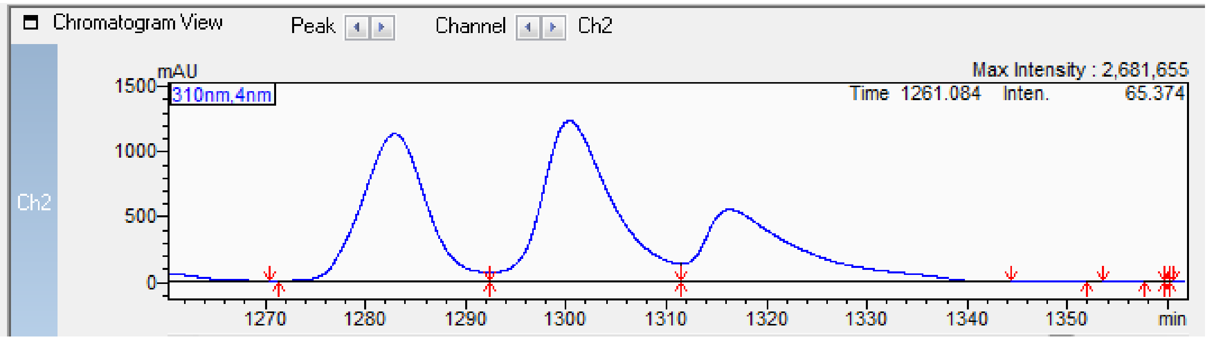

Figure S6. Recycling GPC trace showing separation of the three cage sizes (cage **5**).

Hydrolysis of monoester cage 5e to form cage 5

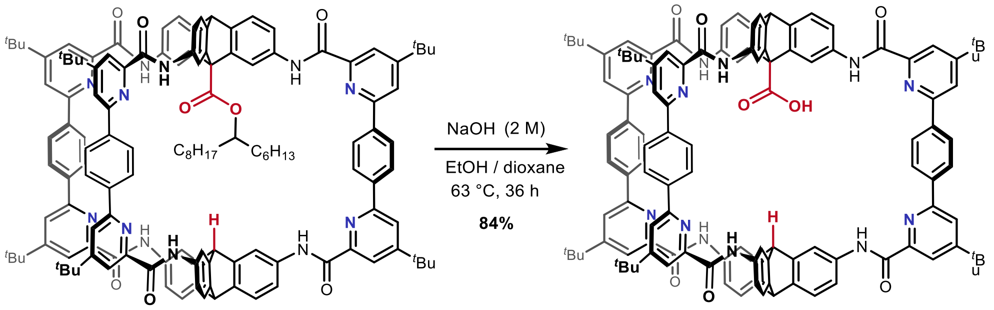

5e **5**

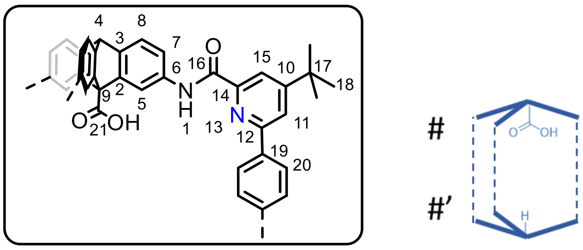

(# and #' = atom numbering)

Monoacid hexapyridine cage 5: Monoalkyl ester cage **5e** (15.7 mg, 7.63 µmol) was suspended in dioxane (2.5 mL) and added was ethanol (2 mL) and an aqueous sodium hydroxide solution (96 mg, 1.0 mL, 2.75 M). The reaction was heated with stirring at 63 °C in an unsealed vial with stirring for 36 h. The reaction was cautiously quenched with dilute HCl until just acidified. The mixture was extracted with ethyl acetate, and the organics washed with water x3 and dried over magnesium sulfate, filtered and concentrated under reduced pressure. The resulting solid was triturated in pentane, and the desired cage **5** was collected by filtration and vacuum dried at 55 °C, 0.3 mbar to give a white solid (11.8 mg, 84%); **^{1}H NMR (600 MHz, $CDCl_3$)** δ 9.94 (s, $3H^{1/1'}$), 9.87 (s, $3H^{1/1'}$), 8.32 (d, *J* = 1.5 Hz, $3H^{11'}$), 8.30 (d, *J* = 1.5 Hz, $3H^{11}$), 8.06 (s, $12H^{20}$), 7.98 (d, *J* = 7.9 Hz, $3H^{7'}$), 7.89 (dd, *J* = 8.1, 1.5 Hz, $3H^{7}$), 7.83 (d, *J* = 1.5 Hz, $3H^{15'}$), 7.82 (d, *J* = 1.5 Hz, $3H^{15}$), 7.69 – 7.64 (m, 3H), 7.50 (d, *J* = 7.6 Hz, 3H), 7.49 (d, *J* = 7.9 Hz, 3H), 7.24 (s, 3H), 5.52 (s, 1H), 5.50 (s, 1H), 5.45 (s, 1H), 1.41 (s, 27H), 1.40 (s, 27H); **^{13}C NMR (151 MHz, $CDCl_3$)** δ 170.6, 163.2, 163.1, 162.8, 162.8, 156.7, 156.7, 150.1, 149.9, 145.4, 142.6, 142.1, 142.0, 140.6, 140.4, 134.4, 134.2, 128.0, 124.4, 124.3, 121.3, 121.3, 120.1, 119.6, 118.9, 118.8, 116.9, 116.8, 61.5, 53.9, 53.2, 52.9, 35.5, 30.7; **MS *m/z*** (MALDI-ToF-RP) (DCTB matrix) 1831.6 ($C_{122}H_{107}N_{12}O_8$, $[M+H]^+$ requires 1831.8).

Mechanistic Experiments

Demonstration of acyl transfer catalysis by cage **1**

Method: Solutions of cage (**1,2,3 or 4**) (0.72 mg, 0.38 µmol, 1 mol%) in $CDCl_3$ (0.550 mL, 0.825 g) were weighed into NMR tubes. Added was acetic anhydride (4.00 µL, 42.3 µmol), mixed by shaking, and a ^{1}H-NMR spectrum recorded immediately. 2-ethyl-hexanol (5.00 mg, 6.00 µL, 38.4 µmol) was then added by microsyringe, subsequent ^{1}H-NMR spectra were recorded (16 scans), and conversion to ester obtained by integration of the AcOC$\underline{H_2}$R protons. Conversion was plotted against time.

The same reaction was also performed with pyridine (2.28 µmol) instead of a cage.

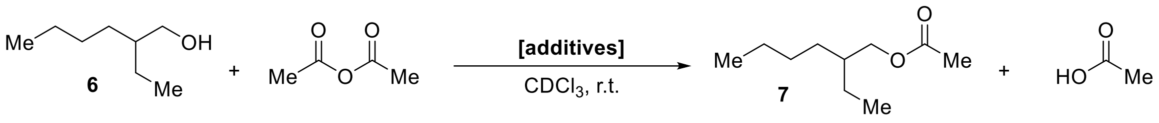

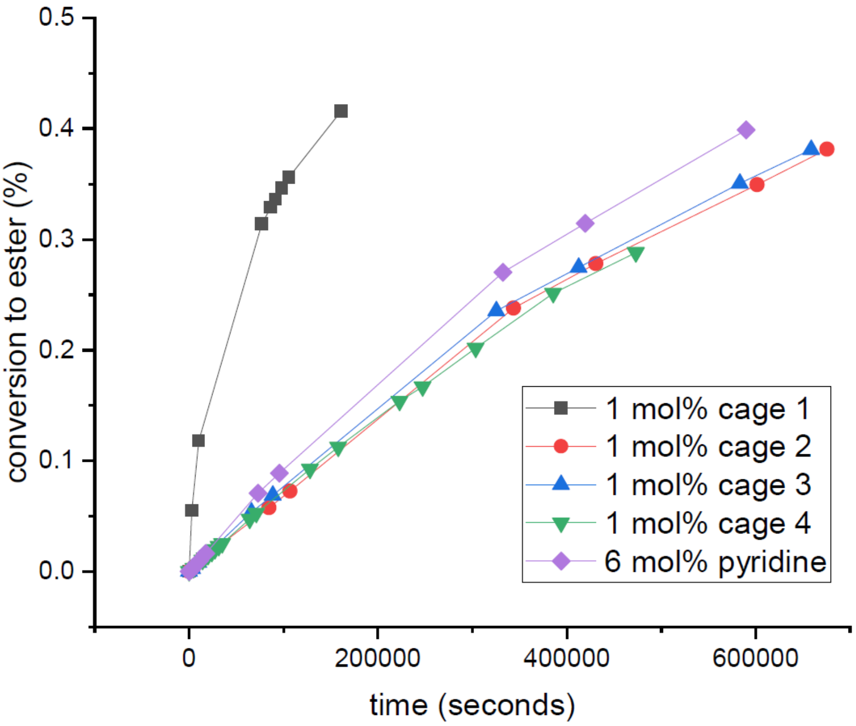

Figure S7. Cage **1** promotes catalysis relative to background and control cages.

Ester **7** has data consistent with the literature:[8] **^{1}H NMR (400 MHz, $CDCl_3$)** δ 4.01 – 3.91 (m, 2H), 2.03 (s, 3H), 1.55 (ddd, *J* = 6.0, 6.0, 6.0 Hz, 1H), 1.40 – 1.21 (m, 8H), 0.91 – 0.83 (m, 6H); **^{13}C NMR (101 MHz, $CDCl_3$)** δ 171.5, 67.1, 38.8, 30.5, 29.0, 23.9, 23.1, 21.1, 14.1, 11.1.

Estimation of background contributions using measured rate constants

For each cage-promoted experiment, a background reaction was completed using the same concentration of pyridine groups (i.e. 6 equivalents wrt cage) to cautiously account for base-catalyzed background reactions.

These reactions were modelled as second order reactions according to:

$$\frac{d[ester]}{dt} = k_{eff}[alcohol][Ac_2O] = -\frac{d[alcohol]}{dt} \qquad \text{(SE1)}$$

where the effective rate constant k_{eff} was assumed to contain all conceivable background pathways, for instance:

$$k_{eff} = k_{bg1} + k_{bg2}[pyridine]^u[AcOH]^v + k_{bg3}[pyridine]^x + k_{bg4}[AcOH]^y$$

In practice, k_{eff} varies only slightly with the concentration of pyridine and acetic acid (see **Table S1**, below). Nonetheless, individual background reactions were recorded for each set of conditions. Therefore, k_{eff} makes a good approximation of the rate constant for the overall background reaction during catalysis. k_{eff} was obtained by measuring the gradient of graphs plotted using the integrated rate equation for a second order reaction:

$$\frac{1}{[A]_t} = kt + \frac{1}{[A]_0} \qquad \text{(SE2)}$$

SE2 was used for reactions where $[alcohol]_0 = [Ac_2O]_0$. For reactions with different concentrations of alcohol and acetic anhydride, SE3 was used:

$$ln\frac{[A]_0[B]}{[A][B]_0} = k([B]_0 - [A]_0)t \qquad \text{(SE3)}$$

where the concentration of alcohol, acetic anhydride, and ester were monitored by ^{1}H-NMR over a reaction. In the above equations, $[A]_t$ is the concentration (M^{-1}) of reagent A at time t (s), $[A]_0$ is the initial concentration (M^{-1}) of reagent A, and k is the second order rate constant. The alcohol concentration was tracked by preference.

To observe the effect of pyridine or acetic acid additives, k_{eff} was measured for the following reactions:

Table S1. Background rate constants: initial rate constants of the esterification reaction of 2-ethyl-hexan-1-ol with acetic anhydride in the presence or absence of pyridine and acetic acid in $CDCl_3$ at 298 K.

ID	additives	[alcohol]	[Ac_2O]	$k_{eff}^{i.r.e.}$ (x10^5 $M^{-1}s^{-1}$)	$k_{eff}^{a.t.p.}$ (x10^5 $M^{-1}s^{-1}$)
**a**	30 mol% pyr, 10 mol% AcOH	0.067	0.316	1.96	1.94
**b**	15 mol% pyr, 5 mol% AcOH	0.067	0.316	1.57	1.52
**c**	15 mol% pyr, 5 mol% AcOH	0.068	0.160	1.10	1.45
**d**	15 mol% pyr, 5 mol% AcOH	0.135	0.159	1.70	1.51
**e**		0.068	0.316	1.09	1.09
**f**	100 mol% AcOH	0.068	0.326	1.25	1.29
**g**	200 mol% AcOH	0.068	0.324	1.43	1.66
**h**	300 mol% AcOH	0.068	0.310	1.93	1.88

The rate constants were calculated using two different approaches, and showed good agreement. $k_{eff}^{i.r.e}$ was calculated using the integrated rate equation (SE2, SE3). $k_{eff}^{a.t.p.}$ (average time points) was calculated using conversion to ester over the reaction according to SE1). [concentrations in M^{-1}]. Data used between 0–40,000 s. $k_{eff}^{a.t.p.}$ was typically used, as it gave the most consistent results.

The measured initial rate constant k_{eff} is calculated under low conversion, so little additional [AcOH] is present. It is clear from entries f,g,h (**Table S1**) that there is a separate acetic acid-catalyzed pathway that affects k_{eff}. k_{eff} increases with acetic acid concentration (i.e. the rate under the conditions studied increases as [AcOH] increases). Therefore, for extended measurements, k_{eff} was measured throughout the background reaction as a function of Ac_2O (and therefore AcOH) and the small variations in k_{eff} could be incorporated into the background calculation

when making corrections. In practice, the change in AcOH in the initial rate period was too small to require this approach.

In cage-catalyzed reactions, the background contribution could be subtracted to leave the cage-catalyzed contribution as follows:

$$\frac{d[ester]}{dt} = -\frac{d[alcohol]}{dt} = k_{bg}[alcohol][Ac_2O] + k_{cage}[cage][alcohol] \qquad \text{(SE4)}$$

$$\frac{d[ester]_{cage}}{dt} = \frac{d[ester]}{dt} - \frac{d[ester]_{bg}}{dt} \qquad \text{(SE5)}$$

To subtract the background contribution of ester for an extended reaction, the background rate constant at conversion *c*, $k_{bg}(c)$, was extracted from linear interpolation of the background kinetic data, and the amount of background ester generated between two time points estimated using equation SE6:

$$\int_{t_1}^{t_2}[ester]_{bg} \approx \frac{[alcohol(t_2)] + [alcohol(t_1)]}{2} . \frac{[Ac_2O(t_2)] + [Ac_2O(t_1)]}{2} . \frac{k_{bg}(c(t_2)) + k_{bg}(c(t_1))}{2} . (t_2 - t_1) \qquad \text{(SE6)}$$

where the amount of ester generated by the background reaction between time points t_2 and t_1 is estimated using the average concentration of alcohol and acetic anhydride between t_2 and t_1, along with the average background rate constant k_{bg} as determined from the concentration of ester at t_2 and t_1.

As stated above, in practice, k_{bg} varies only very slightly over the initial conversions examined for cage-catalyzed reactions. Therefore, SE5 could be used, employing the initial (constant) background rate constants.

This is the approach employed wherever initial data is reported as being “background-corrected”.

k_{cage} is reported as a second order rate constant using equation SE7.

$$k_{cage} = \frac{\frac{d[ester]_{cage}}{dt}}{[cage][alcohol]} \qquad \text{(SE7)}$$

For initial rate measurements, [cage] is approximated as constant and equal to the active acylation species.

Substrate scope for acyl transfer catalysis with cage **1**

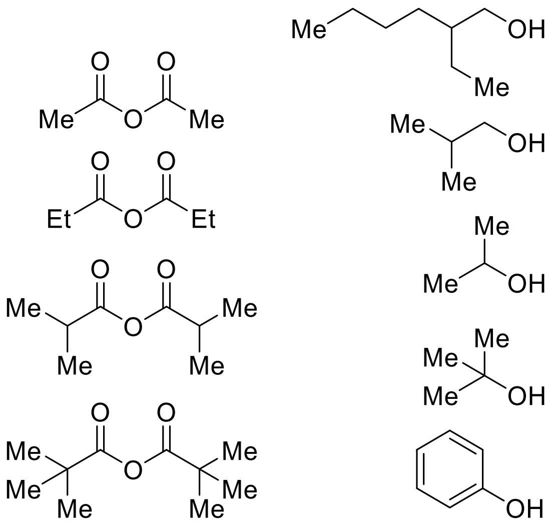

Table S2. Relative initial rates and effective second order rate constants for background and cage-promoted catalysis at 298 K.

	$(R^2CO)_2O$	R^1OH	k_{bg}	+/–		k_{cage}	+/–		$k_{(cage)}/k_{(bg)}$	$v_{i(cage)}/v_{i(bg)}$
a	Me	2-Et-hexyl	9.36	*(0.58)*	x 10^{-6}	1.44	*(0.11)*	x 10^{-1}	15400	170
b	Et	2-Et-hexyl	1.03	*(0.07)*	x 10^{-5}	4.61	*(0.14)*	x 10^{-2}	4470	52.0
c	^{i}Pr	2-Et-hexyl	9.78	*(0.37)*	x 10^{-6}	6.30	*(0.45)*	x 10^{-2}	6440	63.9
d	^{t}Bu	2-Et-hexyl	2.85	*(0.04)*	x 10^{-6}	3.34	*(0.57)*	x 10^{-4}	117	1.50
e	Me	isoamyl	1.26	*(0.01)*	x 10^{-5}	1.57	*(0.26)*	x 10^{-1}	12400	142
f	Me	^{i}Pr	2.52	*(0.05)*	x 10^{-6}	3.84	*(0.3)*	x 10^{-2}	15200	165
g	Me	^{t}Bu	3.71	*(0.04)*	x 10^{-8}	2.88	*(0.09)*	x 10^{-4}	7760	84.5
h	Me	Ph	2.21	*(0.15)*	x 10^{-4}	4.26	*(0.39)*	x 10^{-1}	1930	23.1

The cage-promoted catalytic rate constants $\boldsymbol{k_{(cage)}}$ *($M^{-1}s^{-1}$) are background corrected using the method set out above. The background rate constants* $\boldsymbol{k_{bg}}$ *($M^{-1}s^{-1}$) are the effective background initial rate constants for all background pathways in the presence of pyridine (6 equivs wrt cage). Error calculated as the standard error at 95% confidence intervals from 3 congruent kinetic runs. Error is shown at the same magnitude as the rate constant it is attached to. Shown are second order rate constants:* k_{eff} *= d[P]/dt / ([alc][anhyd]);* k_{cage} *= d[P]/dt / ([alc][cage]); (cage 1.69 mM, alcohol 6.75 mM,* Ac_2O *135-159 mM,* $CDCl_3$*, 298 K).* $\boldsymbol{v_{i(cage)}/v_{i(bg)}}$ *shows the ratio of the initial absolute rates of ester formation for the catalyzed and uncatalyzed reactions at this specific cage/substrate ratio and concentration.*

Note: these conditions were selected to ensure pseudo first order conditions in alcohol. This was achieved by ensuring a large catalyst loading (25 mol% wrt alcohol) and >20-fold excess of cofactor anhydride to ensure catalyst-cofactor activation was not limiting. Pyridine (6 equiv relative to cage) is included in the background to provide a generous maximum “base-catalyzed” contribution by the cage pyridines separate to any cavity-promoted reactions.

Kinetic Esterification Measurement Method: A stock solution of cage was prepared using (*n*+1)(1.80 mg cage **1**, 0.96 µmol) in (*n*+1)(0.550 mL $CDCl_3$, weighed assuming a density of 1.500 g/mL at 25 °C). Weighed into *n* NMR tubes was an aliquot of cage **1** solution (0.5500 mL, 0.8250 g). Added to each NMR tube was the appropriate alcohol as 10 µL of a stock solution prepared such that 10 µL stock was equivalent to 3.84 µmol alcohol. The NMR tubes were sealed and mixed and measurement commenced within 5 h. For measurement, the tube was inserted into an NMR probe (500 MHz) and the sample locked and shimmed and an initial proton spectrum recorded. Working quickly, the sample was ejected from the probe, the anhydride (0.077-0.090 mmol) added at a noted time, the tube shaken to mix, and the tube reinserted. A series of ^{1}H-NMR spectra were recorded, using the measurement

start/end time function to track conversion over time, calibrating the zero point to the anhydride addition and time points at the central time point of the measurement duration. Conversion to product was calculated using the MestReNova Advanced Data Analysis Integrals function, which allows time-annotated integrals to be extracted from stacks of spectra. Conversions were calculated from ratios of integrals e.g. alcohol HOC**H**$_n$– calculated relative to ester AcOC**H**$_n$– setting the total integral to 1 (i.e. all conversions were automatically corrected by internal referencing). Initial rates were extracted from linear fits of at least 5 data points, and pseudo second order rate constants calculated using equation **SE1** for the background and equation **SE7** for cage-catalyzed reactions after subtraction of the background contribution **SE5**, as described above. Background rates were measured in the presence of pyridine (10 mM) to take into account any pyridine-catalyzed conversion.

Initial rates data

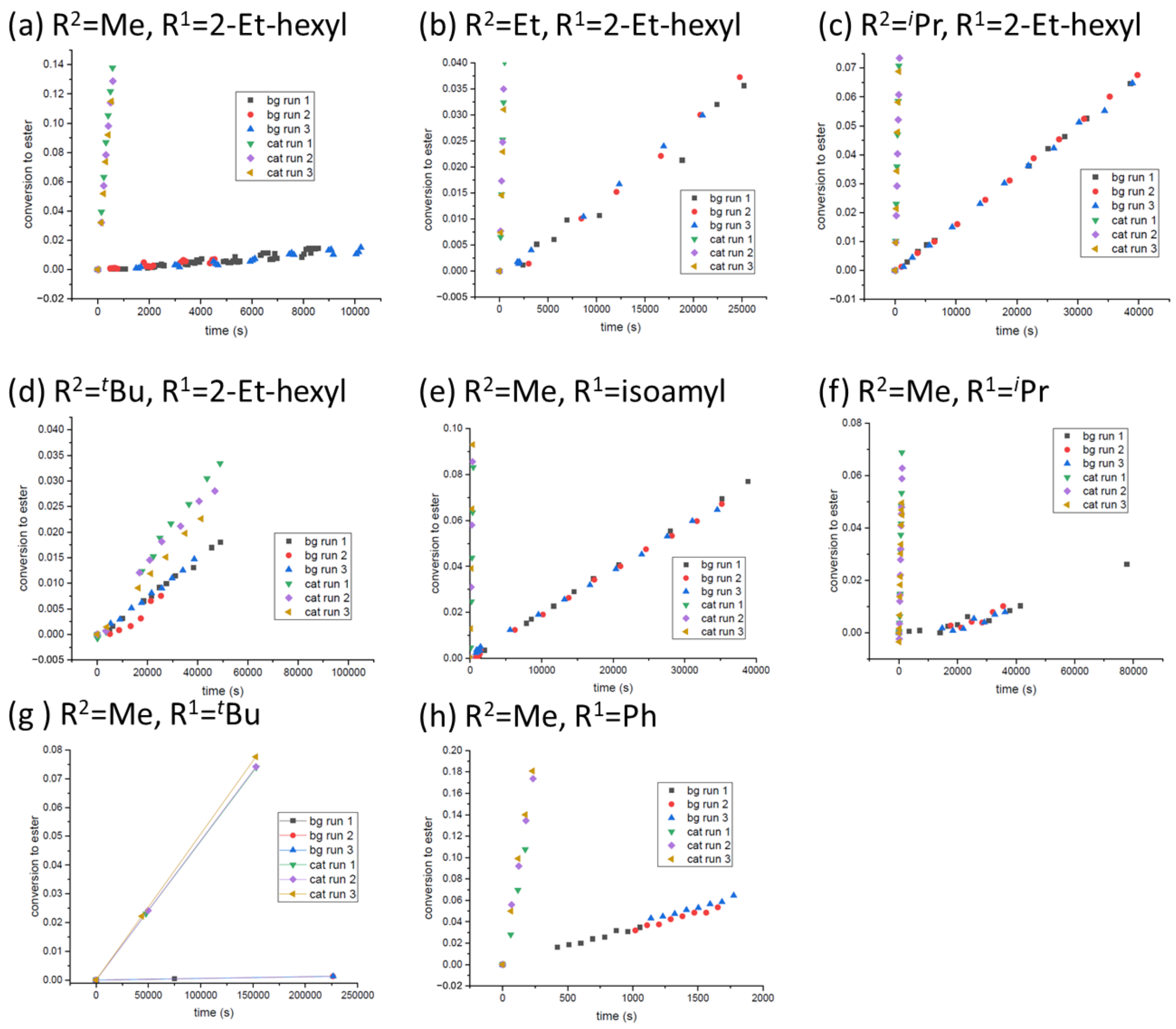

Figure S8. SAR initial rates data for different alcohols and anhydrides in the presence of pyridine (bg=background) or catalyst (cat=cage **1**). Reaction (g) was very slow, and so the rates are estimated from the conversion at two time points.

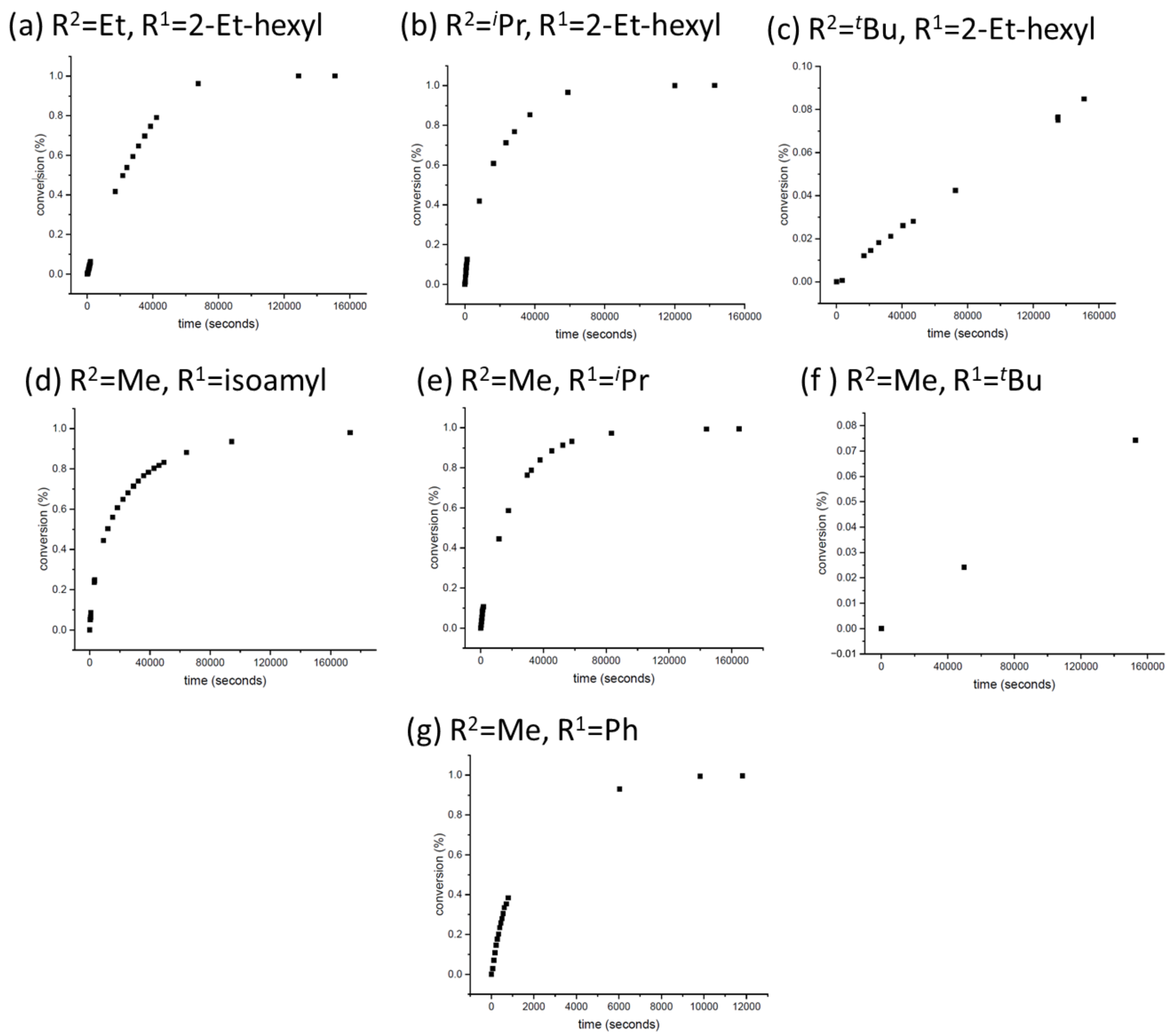

Figure S9. Full catalysis profiles. Examples of cage-**1**-catalyzed esterification data over 160,000 seconds (~44 h), 298 K, $CDCl_3$, where R^2 is the anhydride alkyl group, and R^1 is the alcohol identity.

Figure S10. Reaction of cage **1** with different anhydride cofactors

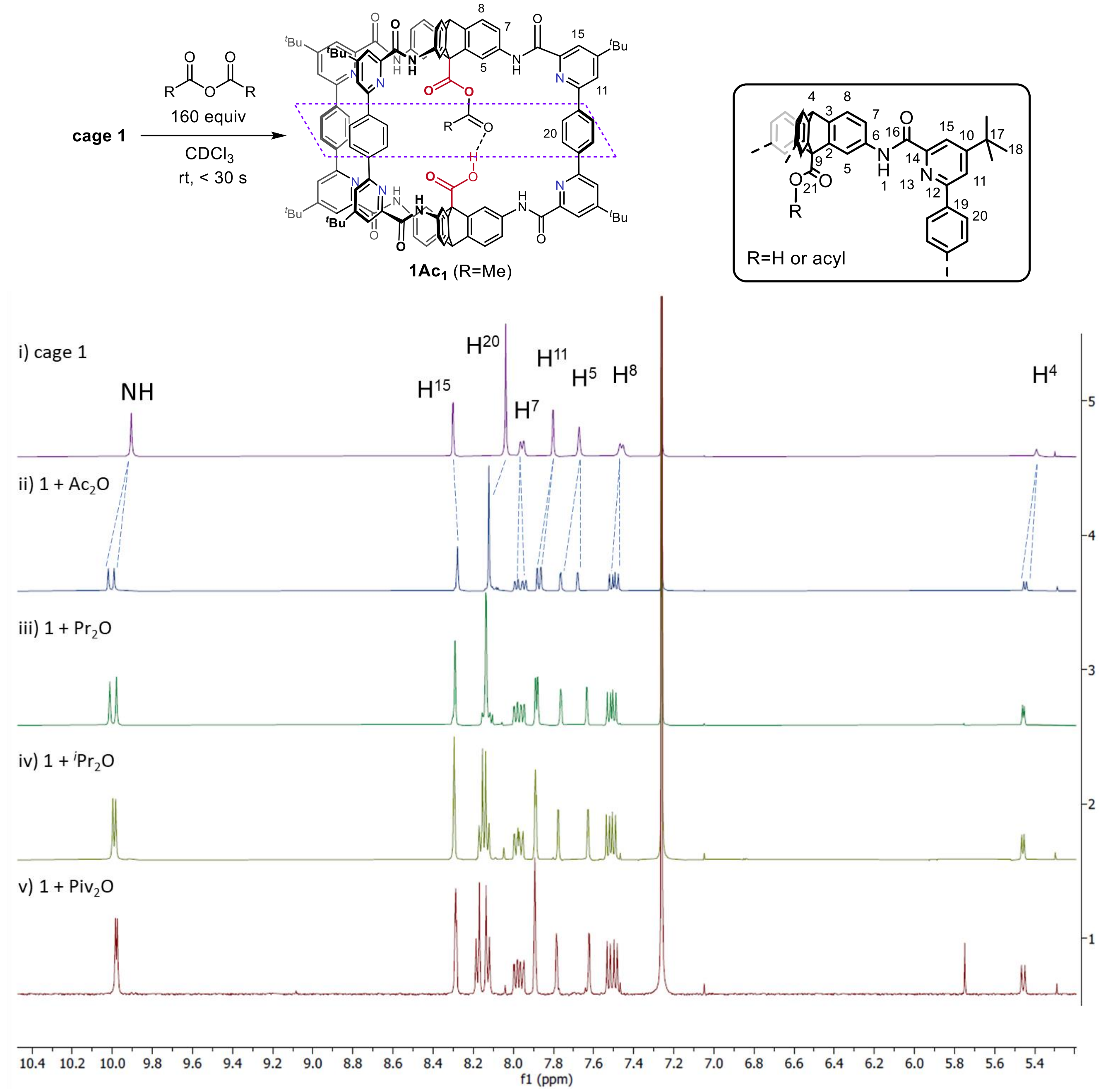

Method: Solutions of cage (0.00175 M) in $CDCl_3$ were weighed into NMR tubes (0.550 mL, 0.825 g) (final cage concentration 1.69 mM). Added was the relevant anhydride (160 equiv, 0.154 mmol) and ^{1}H-NMR spectrum recorded immediately.

Ac_2O: **$1Ac_1$** ^{1}H DATA: ^{1}H NMR (500 MHz, $CDCl_3$) δ 10.02 (s, 3H), 9.99 (s, 3H), 8.28 (d, *J* = 1.7 Hz, 3H), 8.28 (d, *J* = 1.7 Hz, 3H), 8.12 (s, 12H), 7.98 (dd, *J* = 8.1, 1.7 Hz, 3H), 7.95 (dd, *J* = 8.1, 1.7 Hz, 3H), 7.88 (d, *J* = 1.7 Hz, 3H), 7.86 (d, *J* = 1.7 Hz, 3H), 7.76 (d, *J* = 1.7 Hz, 3H), 7.68 (d, *J* = 1.7 Hz, 3H), 7.51 (d, *J* = 8.1 Hz, 3H), 7.48 (d, *J* = 8.1 Hz, 3H), 5.45 (s, 1H), 5.44 (s, 1H), 2.90 (s, 3H), 1.40 (s, 27H), 1.40 (s, 27H).

Pr_2O: **$1Pr_1$** ^{1}H DATA: ^{1}H NMR (500 MHz, $CDCl_3$) δ 10.01 (s, 3H), 9.98 (s, 3H), 8.29 (d, *J* = 1.7 Hz, 3H), 8.29 (d, *J* = 1.7 Hz, 3H), 8.16 – 8.11 (m, 12H), 7.99 (dd, *J* = 8.1, 2.0 Hz, 3H), 7.95 (dd, *J* = 8.1, 2.0 Hz, 3H), 7.89 (d, *J* = 1.6 Hz, 3H), 7.88 (d, *J* = 1.6 Hz, 3H), 7.76 (d, *J* = 2.0 Hz, 3H), 7.63 (d, *J* = 2.0 Hz, 3H), 7.52 (d, *J* = 8.1 Hz, 3H), 7.50

(d, J = 8.1 Hz, 3H), 5.46 (s, 1H), 5.45 (s, 1H), 3.05 (q, J = 7.3 Hz, 2H), 1.77 (t, J = 7.3 Hz, 3H), 1.41 (s, 27H), 1.41 (s, 27H).

$^{i}Pr_2O$: **1^{i}Pr$_1$** ^{1}H DATA: ^{1}H NMR (500 MHz, $CDCl_3$) δ 10.00 (s, 3H), 9.98 (s, 3H), 8.31 – 8.29 (m, 6H), 8.16 (d, J = 8.5 Hz, 6H), 8.13 (d, J = 8.5 Hz, 6H), 7.99 (dd, J = 8.2, 2.1 Hz, 3H), 7.96 (dd, J = 8.2, 2.1 Hz, 3H), 7.90 – 7.88 (m, 6H), 7.78 (s, 3H), 7.63 (s, 3H), 7.53 (d, J = 8.1 Hz, 3H), 7.50 (d, J = 8.1 Hz, 3H), 5.46 (s, 1H), 5.45 (s, 1H), 3.12 (hept, J = 6.8 Hz, 1H), 1.70 (d, J = 6.8 Hz, 6H), 1.42 (s, 27H), 1.41 (s, 27H).

Piv_2O: **1Piv$_1$** ^{1}H DATA: ^{1}H NMR (500 MHz, $CDCl_3$) δ 9.98 (s, 3H), 9.97 (s, 3H), 8.31 – 8.26 (m, 6H), 8.18 (d, J = 8.4 Hz, 6H), 8.13 (d, J = 8.4 Hz, 6H), 7.99 (dd, J = 8.1, 2.0 Hz, 3H), 7.96 (dd, J = 8.1, 2.0 Hz, 3H), 7.91 – 7.87 (m, 6H), 7.78 (d, J = 2.0 Hz, 3H), 7.62 (d, J = 2.0 Hz, 3H), 7.52 (d, J = 8.1 Hz, 3H), 7.49 (d, J = 8.1 Hz, 3H), 5.47 (s, 1H), 5.45 (s, 1H), 1.63 (s, 9H), 1.41 (s, 27H), 1.38 (s, 27H).

Crystal methods

Crystallization was performed using the double vial method, with the outer vial containing the lower boiling solvent (e.g. *n*-pentane), which was allowed to slowly diffuse by evaporation into an inner vial containing a solution of high boiling solvent (e.g. THF/EtOAc or DCM/Ac_2O) containing cage (0.5 mg in ~100-200 µL). The vials were stored in a dark cupboard at ambient temperature (~20 °C) away from light or vibrations, and crystals grew over a timescale of 1-2 weeks.

Similarly to processing the crystal data for cage **2**,[9] it was not possible to accurately determine any individual solvent molecules such that Solvent Masking[10] was employed. However, the overall basic skeletal architecture of each cage is well defined. Also, in each case the two cage molecules only really differ in the extremities of the cages, such as some of the *t*-butyl groups along with the likely orientation of the internal acid groups.

Crystal Data for **1Ac₁** $C_{173}H_{210}Cl_{12}N_{12}O_{56}$ (including solvent), Mr = 3778.92, triclinic, P1 (No. 1), a = 20.0800(4) Å, b = 22.0181(6) Å, c = 24.9570(5) Å, a = 97.603(2)°, b = 92.367(2)°, g = 102.487(2)°, V = 10650.5(4) Å3, T = 100(2) K, Z = 2, Z' = 2, m(Cu Ka) = 2.059 mm-1, 171084 reflections measured, 63954 unique (Rint = 0.0720) which were used in all calculations. The final wR2 was 0.2103 (all data) and R1 was 0.0742 (I≥2 s(I)).

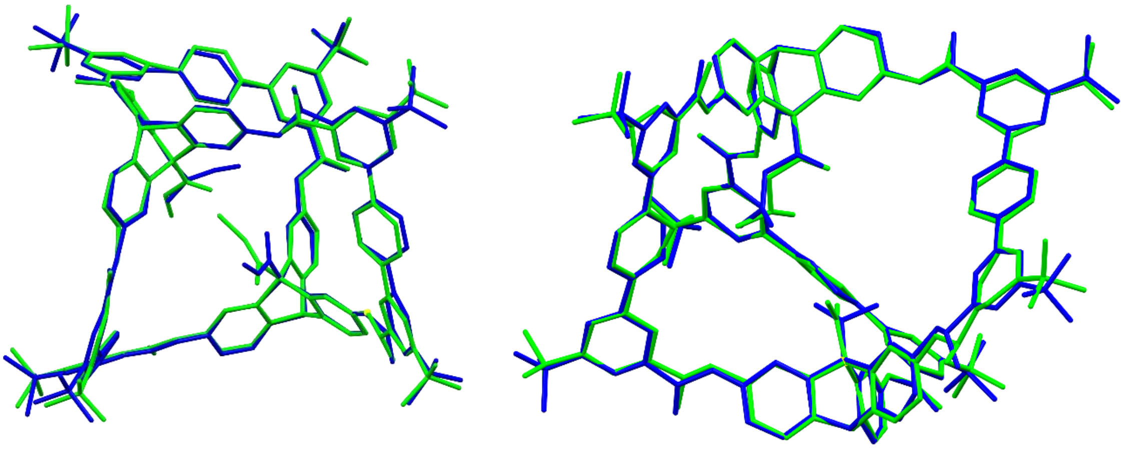

Figure S11. Overlaid images of crystal structures **2** (green) and **$1Ac_1$** (blue). Two perspectives are presented.

Full crystallography data is available in separate crystallography files.

Note on biaryl twisting: In recent work, we show how the twisting of one triptycene relative to another requires the edge piece terphenyl groups to adjust their conformation.[9] The distance between the amide groups must remain the same, but the amide bonding vectors to the triptycenes must now be twisted. Much of this twisting can be accommodated by an increase in the biaryl dihedral angle in the terphenyl units. This small reduction in aryl-aryl conjugation is preferred over bond angle strain. Overall, the effect is that the triptycene groups get closer together, reducing the acid-carbon acid-carbon distance, r_{CC}. The triptycenes would be expected to get closer if an attractive hydrogen bond were present to "pay for" some of the twisting strain. The NMR data for **$1Ac_1$** compared to **1** is suggestive of this biaryl twisting in solution, and therefore supportive of the presence of a hydrogen bond in solution.

Conversion of **$1Ac_1$** and **$1Ac_2$** to ester in isolation

Method to prepare $CDCl_3$ solutions of acylated cage with no Ac_2O or AcOH present: Solutions of cage (1.67 mM) in $CDCl_3$ were weighed into NMR tubes (0.550 mL, 0.825 g). Added was acetic anhydride (2 uL, excess) and the tube shaken, and immediately placed under high vacuum (~0.5 mbar). The tube was dried over 24 h; during this time, $CDCl_3$ (0.1 mL) was added and the tube placed back under vacuum (~4 times), in order to remove Ac_2O and AcOH. Then, $CDCl_3$ (0.550 mL, 0.825 g) was weighed into the tubes to make solutions of mixtures of cage **1**, **$1Ac_1$** and **$1Ac_2$** without Ac_2O or AcOH present.

Kinetics of **$1Ac_1$** and **$1Ac_2$**: To a solution containing a mixture of **1**, **$1Ac_1$** and **$1Ac_2$** (0.959 µmol) without Ac_2O or AcOH present in $CDCl_3$ (0.550 mL) in an NMR tube was added alcohol **6** (2 µL, 0.0128 mmol) and NMR spectra recorded immediately every 120 s.

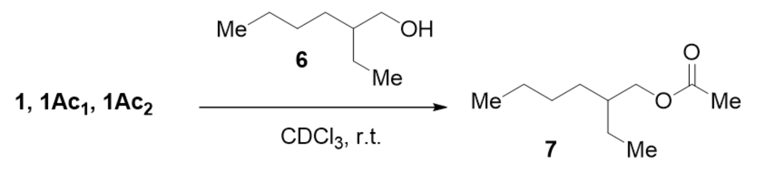

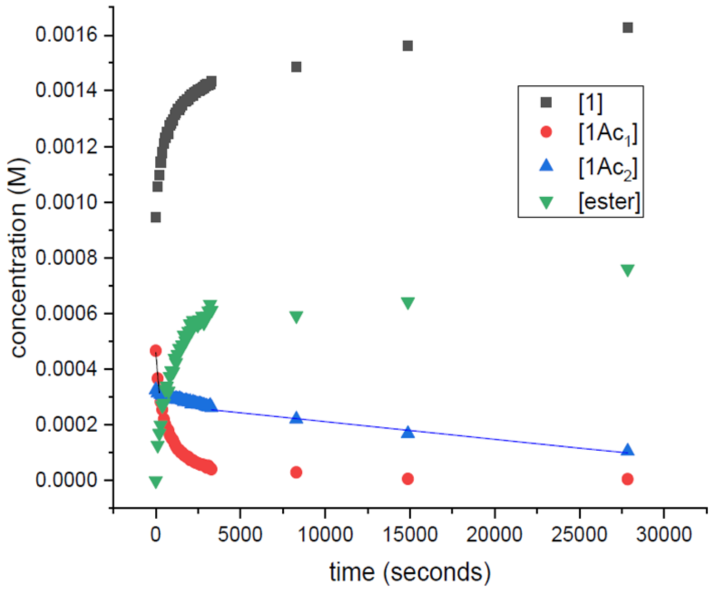

Figure S12. $1Ac_1$ is the active acylating agent. In the presence of alcohol and absence of Ac_2O, **$1Ac_1$** is consumed rapidly and ester forms at the same rate. **$1Ac_2$** is consumed 10^7 times more slowly, correlated with slower ester formation. Rates were extracted for **$1Ac_1$** and **$1Ac_2$** using linear fits in suitable time periods (lines shown).

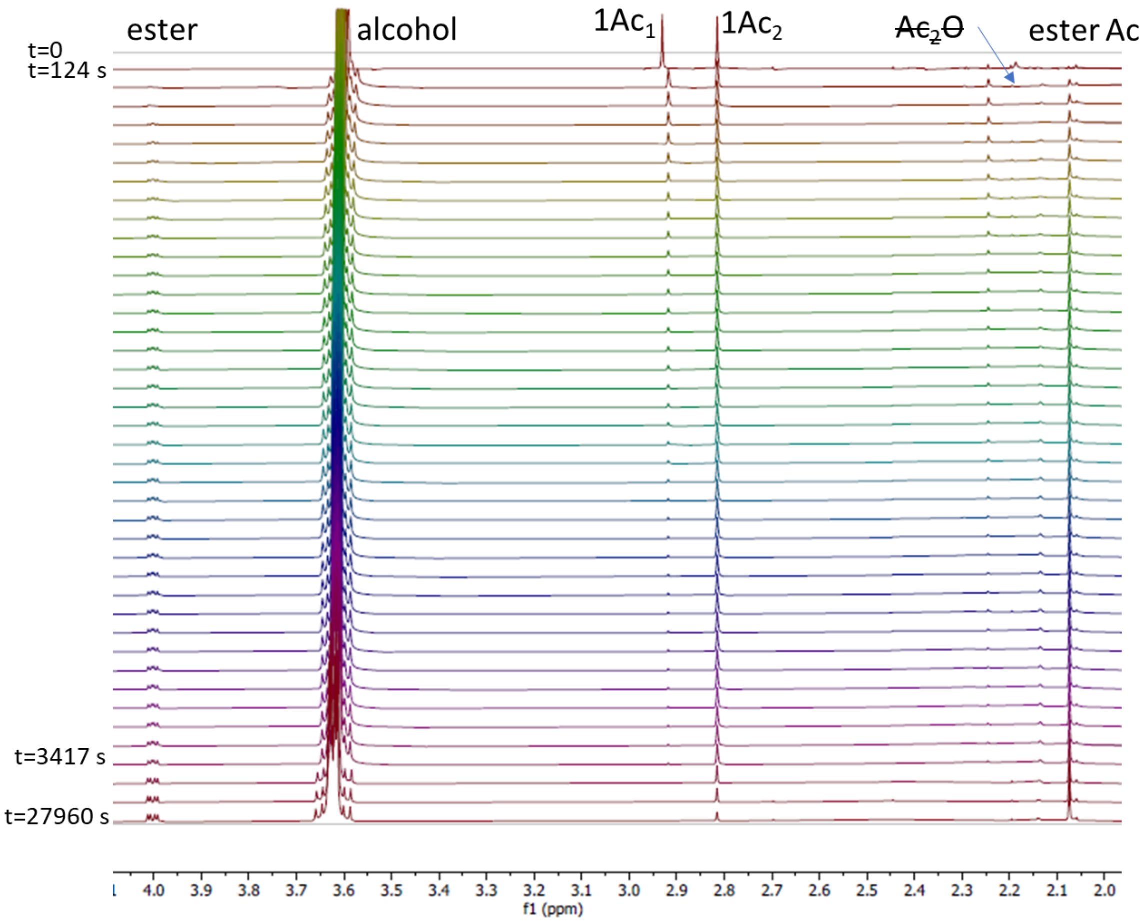

Figure S13. Consumption of **1Ac₁** and **1Ac₂**. In the absence of acetic anhydride, a mixture of cage **1**, monoacylated cage **1Ac₁** and bisacylated cage **1Ac₂** convert to ester in the presence of alcohol **6**. **1Ac₁** is converted faster than **1Ac₂**.

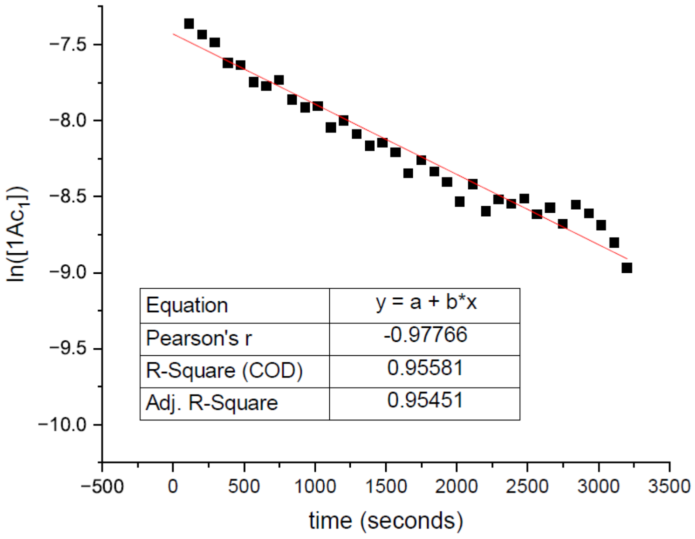

Figure S14. Ester formation is first order in **1Ac₁** (0.33 mM) in the presence of excess alcohol **6** (23 mM) ($CDCl_3$, 298 K). A linear fit of ln[**1Ac₁**] against *t* under pseudo first order conditions is consistent with first order consumption of **1Ac₁**.

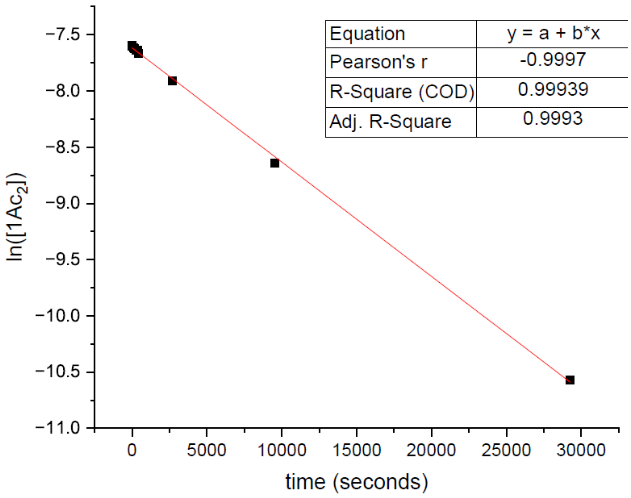

Figure S15. First order consumption of **$1Ac_2$** (0.5 mM) in the presence of excess alcohol 6 (69 mM) ($CDCl_3$, 298 K); measured with no **$1Ac_1$** present.

Acyl exchange reactions of cage **1**

Acyl exchange reactions of cage **1** demonstrate two forms of catalysis: To probe the reactivity of the monoacylated cage **1Ac₁**, propionic acid (propanoic acid) was added to a solution of acetic anhydride in $CDCl_3$ with and without 1 mol% cage **1** (0.68 mM) and the population of anhydride species measured by ^{1}H-NMR over 7 h. Equilibrium of the three anhydride species was reached after ~50 minutes in the presence of cage **1** (**Figure S16i**) whilst without the cage, conversion to $(EtCO)_2O$ was only ~70% of the equilibrium value after 7 h (**Figure S16ii**). In the cage experiment, monoanhydride cage **1Ac$_1$** formed immediately and reached a steady state ^{1}H-NMR ratio of ~1:1 with the propionic-cage mixed anhydride **1(COEt)$_1$** on the same time scale as the small molecule anhydrides (50 minutes, **Figure S17-19**), consistent with the hypothesis that the monoanhydrides (and not the bisanhydrides) play a key role in acyl exchange catalysis. This rapid reaching of two equilibria requires two forms of catalysis to be operative: i) activation of cage **1** as a nucleophile (relative to free acid) and/or anhydride in the cage as an electrophile (relative to free anhydride), and ii) activation of the monoacylated cage species as an electrophile (relative to free anhydride) and/or of acid as a nucleophile (relative to free acid). This is analogous to enzymatic catalysis, where an overall rate acceleration requires both cofactor activation and substrate reaction to be faster than the single background rate-determining step (**Figure 1a**, main manuscript).

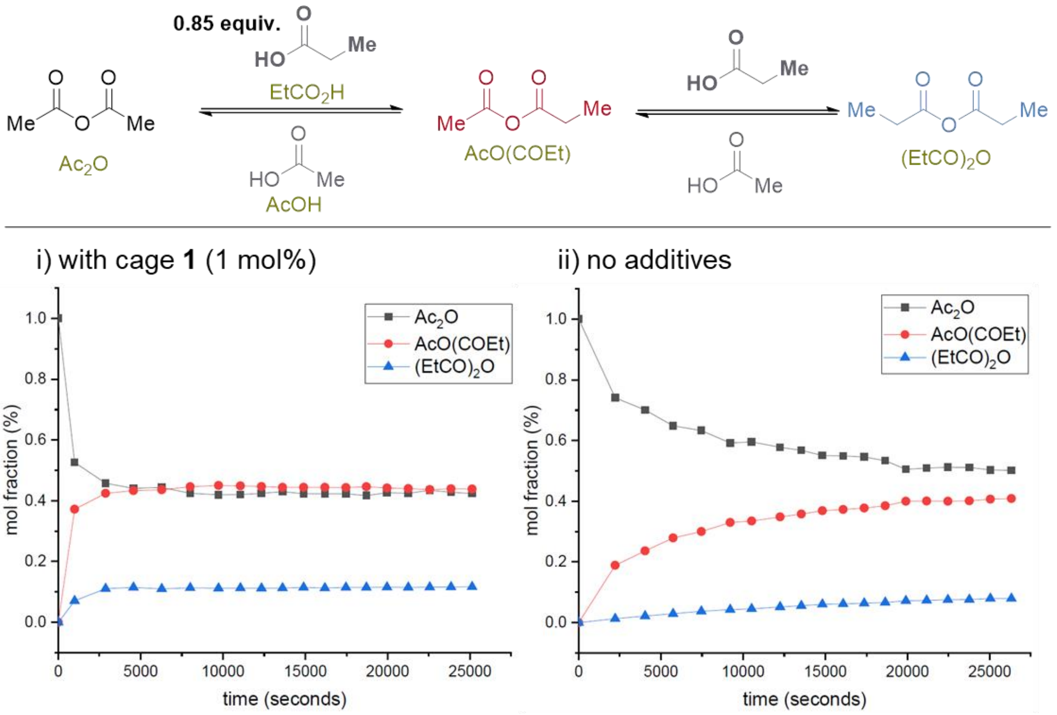

Figure S16. Anhydride/acid scrambling rates with/without 1 mol% cage **1**.

Method: Cage **1** (0.72 mg, 0.38 µmol) was dissolved in $CDCl_3$ (0.550 mL, 0.825 g) in an NMR tube. Added was acetic anhydride (4.0 µL, 42 µmol) and ^{1}H-NMR spectrum recorded immediately. Immediately thereafter was added propanoic acid (2.7 µL, 36 µmol) and NMR spectra recorded over several time points. The same reaction was recorded in the absence of cage **1**. Ratios of anhydrides/acids were measured by integration. A statistical equilibrium was reached, with the ratio of products observed to be 4:4:1 Ac_2O:AcO(COEt):$(EtCO)_2O$). The concentrations of cages could also be tracked, and shows an initial spike for the **1Ac$_1$** before conversion to other cages is observed (**Figure S17**).

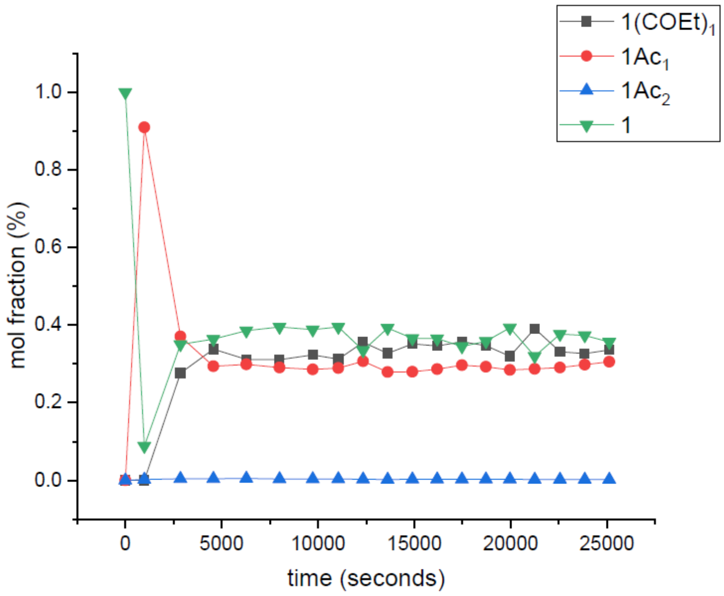

Figure S17. Cage speciation kinetics during the anhydride exchange reaction show a roughly 1:1:1 equilibrium mixture of monoanhydrides **1Ac$_1$** and **1(COEt)$_1$**, and empty cage **1**. This speciation is consistent with the assertion that the monoacylated cages are responsible for catalysis.

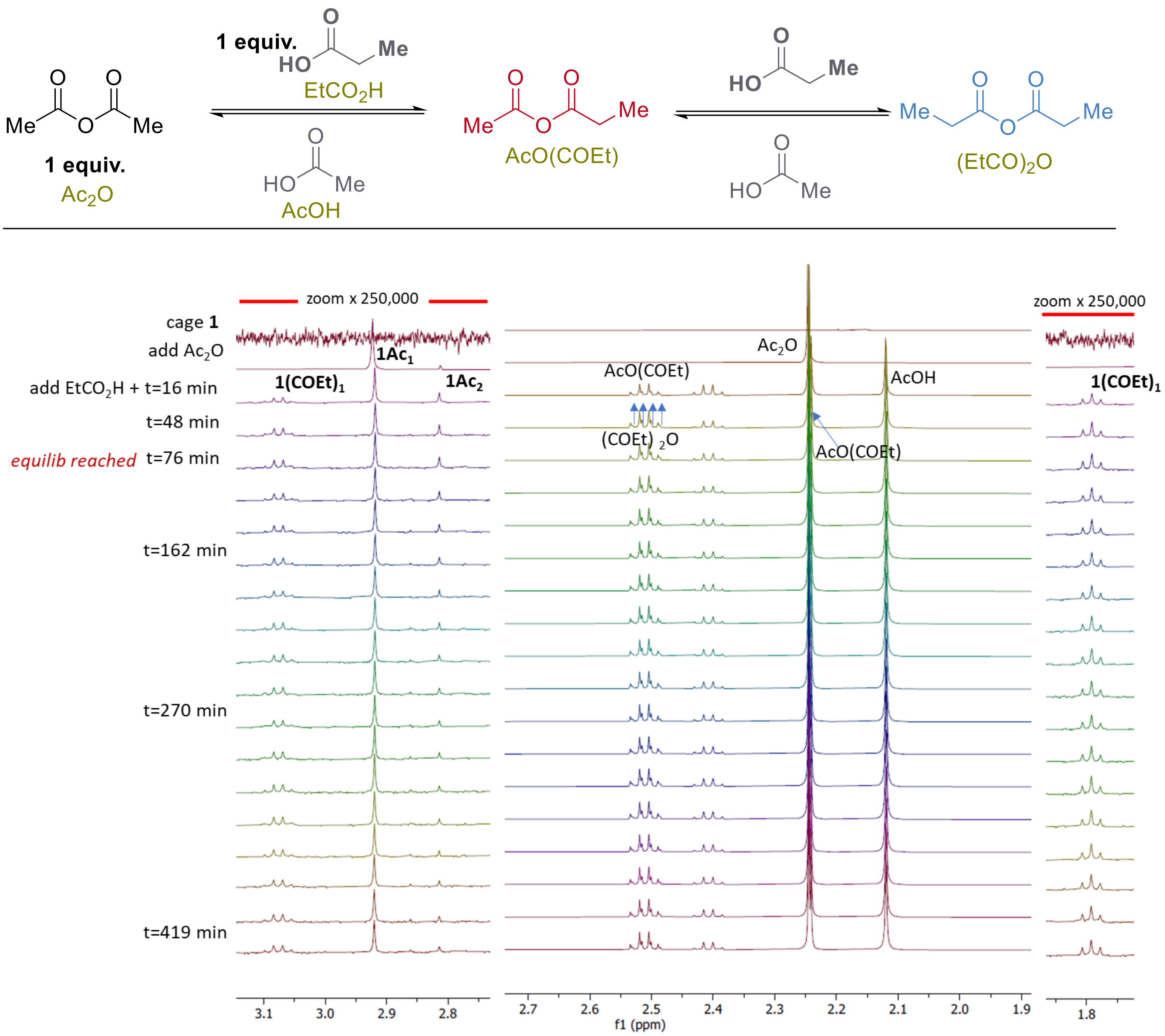

Figure S18. ^{1}H-NMR stack showing equilibrium of acetic anhydride with propionic acid in the presence of cage **1**. Equilibrium is reached within ~50 min. Visible are the monoacetylated cage **1Ac$_1$** and propionic cage monoanhydride **1(COEt)$_1$** in a 1:1 ratio. A small amount of bis-acetylated cage **1Ac$_2$** is also visible.

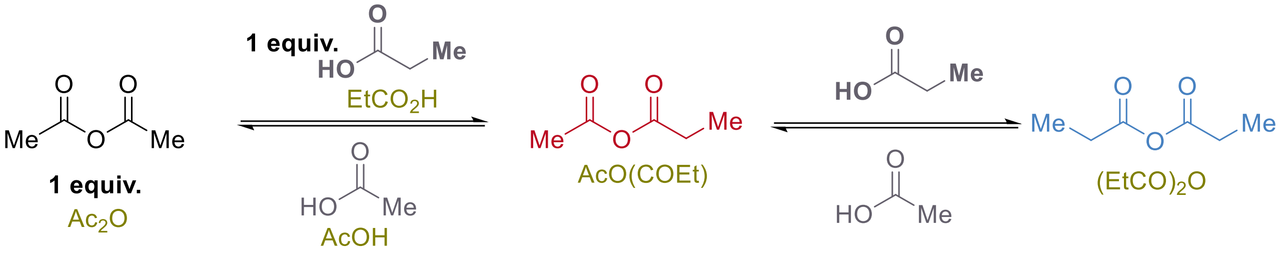

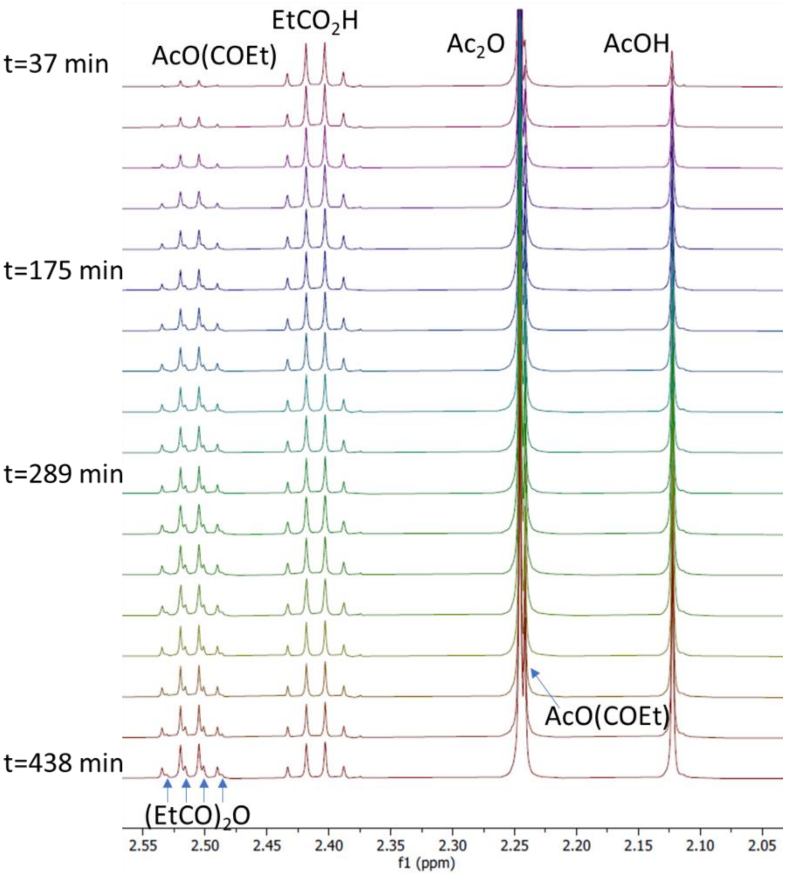

Figure S19. ^{1}H-NMR stack showing reaction of acetic anhydride with propionic acid in the absence of cage (background). Equilibrium is still not reached after 438 min.

Michaelis-Menten analysis of cage **1** with acetic anhydride and 2-ethylhexanol

Due to inhibition effects by acetic acid, the initial rates method was necessary to accurately measure the direct effect of substrate concentration on catalysis. Excess Ac_2O and constant cage **1** were used. All product formation was background corrected as described above. The resulting data could be fit using non-linear regression to the Michaelis-Menten equation, which is preferred to linear-regression.[11]

Method: Cage **1** was dissolved in $CDCl_3$ (0.550 mL, 0.825 g) in an NMR tube. Added was acetic anhydride (8.56 µL, 0.0906 mmol) and ^{1}H-NMR spectrum recorded immediately. Immediately thereafter was added 2-ethylhexanol from a stock solution (various) and NMR spectra recorded over several time points. The total volume was constant (+/– 5 µL) between runs. Initial rates were extracted from, plotted as a function of substrate concentration, and fit to the Michaelis-Menten equation in the software Origin Pro using non-linear regression. A minimum of three consistent initial rates were used.

$$\upsilon = \frac{d[ester]}{dt} = \frac{V_{max}[S]}{K_M+[S]} \quad \text{(SE8)}$$

where υ is the rate of (ester) product formation, V_{max} represents the maximum rate when the catalyst (enzyme) is saturated with substrate, [S] is the concentration of substrate, and K_M is the Michaelis constant, which is equal to the substrate concentration at half V_{max}, and has units M (i.e. is the inverse of a typical association constant, K_a).

Table S3. Initial esterification rate dependency on substrate (alcohol) concentration at constant cage concentration.

[alcohol]/[cage]	[alcohol] (M)	d[ester]/dt ($M^{-1}s^{-1}$)	error	error (%)
4	6.75E-03	1.49E-06	1.18E-07	8%
8	1.35E-02	5.23E-06	3.48E-07	7%
12	2.03E-02	6.85E-06	5.71E-07	8%
20	3.42E-02	1.07E-05	1.00E-06	9%
40	6.80E-02	1.71E-05	1.88E-06	11%
80	1.35E-01	2.65E-05	3.03E-06	11%
120	2.00E-01	3.28E-05	2.73E-06	8%

standard error in the initial rate quoted at 95% confidence intervals for a minimum of three repeats.

$K_M = 1.6 \times 10^{-1}$ M (±0.14×10^{-1} M) (from fit, see graph below)

$V_{max} = 5.9 \times 10^{-5}$ M s^{-1} (±0.29×10^{-6} M s^{-1}) (from fit, see graph below)

$k_{cat} = 3.5 \times 10^{-2}$ s^{-1} (±0.17×10^{-2} s^{-1}) = V_{max} / [cage] = 5.9×10^{-5} M s^{-1} / [0.0017 M]

(note: this value of k_{cat} is the value commonly extracted from the Michaelis-Menton equation; the value k_{cage}, which is quoted elsewhere, is a standard second order rate constant, as defined in **Table 1**.)

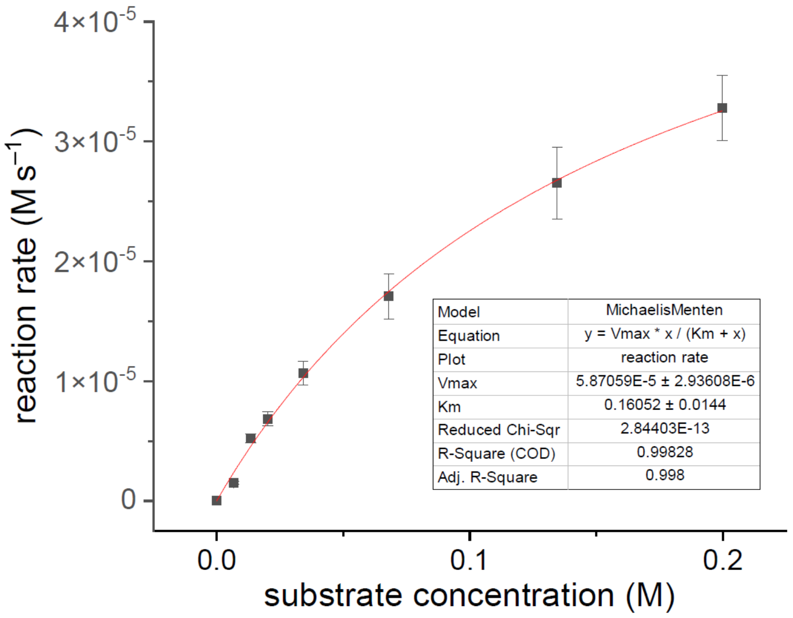

Figure S20. Michaelis-Menten fit of data in **Table S3**.

Notes on the Michaelis-Menten analysis

The Michaelis-Menten analysis is dependent on a steady-state approximation. In the case of this reaction, the steady state approximation is valid only when the re-activation of cage **1** to form the proposed active species, **1Ac$_1$**, is not limiting, which can be the case at higher substrate concentrations. In this case, E (and therefore ES) in equation SE# would be lower than expected. Therefore, we used the initial rates method with an excess of Ac_2O and 25 mol% cage, and processed data within the first 300 seconds.

$$E + S \rightleftharpoons \mathrm{ES} \rightarrow \mathrm{E} + \mathrm{P} \qquad \text{(SE9)}$$

$$1 + Ac_2O + S \rightarrow 1Ac_1 + S + AcOH \rightleftharpoons 1Ac_1.S + AcOH \rightarrow 1 + \mathrm{ester} + AcOH$$

Increasing AcOH concentration also has an effect on the equilibrium position of **1** and **1Ac$_1$**. This effect is minimal under the initial rate assumptions, and indeed, titrating relevant amounts of AcOH (i.e. the amounts naturally formed) into the initial state of a reaction does not affect [**1Ac$_1$**] or the observed binding of the alcohol to **1Ac$_1$** significantly (**Figure S19**, below) due to the excess of Ac_2O used.

The increasing AcOH concentration may also interfere with the pyridyl groups in the cage cavity. d[AcOH]/dt is proportional to d[ester]/dt, so it is not trivial to decouple rate inhibition due to increasing [AcOH] from reduced rate with increasing alcohol concentrations due to saturation binding kinetics.

For these reasons, we prefer to discuss second order rate constants rather than the pseudo first order rate constant k_{cat}. For the same reason, the measured value of K_M must be treated as an approximation. However, the strong data presented in **Figure 4b** (manuscript) indicating a close interaction of the alcohol and **1Ac$_1$** (but not **1Ac$_2$**) and the fact that the initial rates are estimated within 300 s when the steady-state approximation is strongest, means we conclude an initial binding is likely dominating the non-linear kinetics.

As noted above, titrating AcOH into the **1Ac$_1$** complex does not afford strong shift changes for the internal acyl methyl group, unlike additions of alcohol (**Figure S21**).

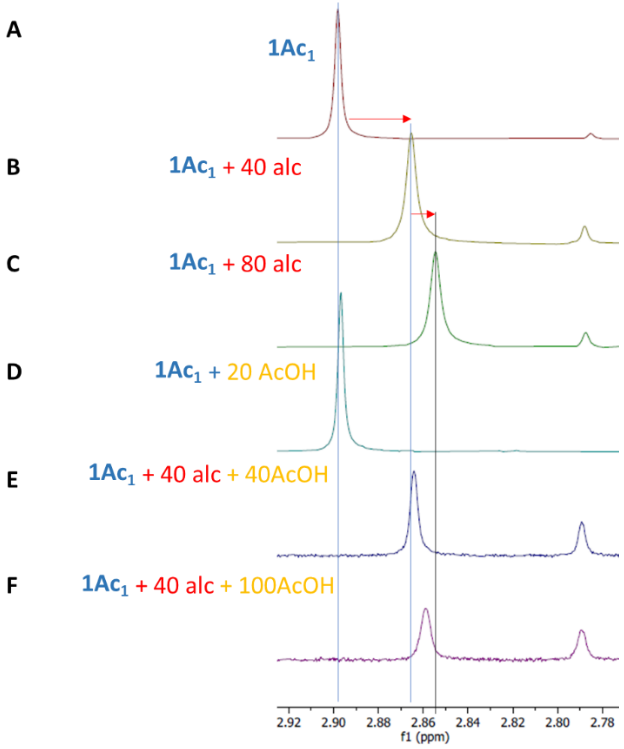

Figure S21. Alcohol binding in the cavity is not strongly affected by AcOH concentration. Increasing concentration of alcohol (**A,B,C**), and not AcOH (**D,E,F**), is responsible for the majority of the **$1Ac_1$** methyl group ^{1}H-NMR shifts ($CDCl_3$), supporting preferential interaction of the cage cavity of **$1Ac_1$** with the alcohol (and not acetic acid). **$1Ac_2$** (peak on the right) is relatively unaffected.

The effect of additives on esterification catalysis with cages

Acetic acid and ester product were used as additives to probe inhibition or enhancement effects on the cage **1** promoted esterification catalysis of acetic anhydride with 2-ethylhexanol. When acetic acid (34 mM, 50 mol% with respect to alcohol) is added at the start of cage **1** (0.64 mM)-promoted esterification reactions between acetic anhydride and alcohol **6** (69 mM), the catalytic esterification rate is inhibited significantly (**Figure S22**). Catalysis is accelerated marginally by inclusion of 100 mol% ester product (indicating that product inhibition is not a problem for this system); the ester likely reduces the effective acidity of AcOH (**Figure S22**).

Method: A solution of cage **1** in $CDCl_3$ (0.698 mM) was weighed (0.550 mL, 0.825 g) into an NMR tube (0.384 µmol of cage, 1 mol%). Added was 2-ethylhexanol 6.00 µL, 38.4 µmol), the chosen additive, and acetic anhydride (4.28 µL, 38.4 µmol) and NMR spectra recorded over several time points. Conversion to ester against time was plotted. These data are not background corrected.

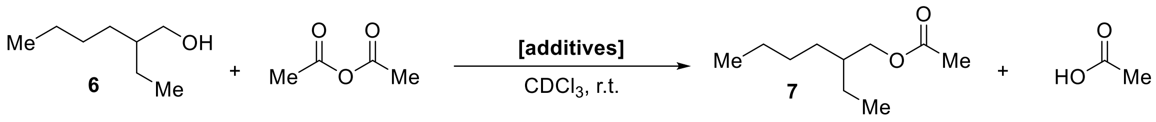

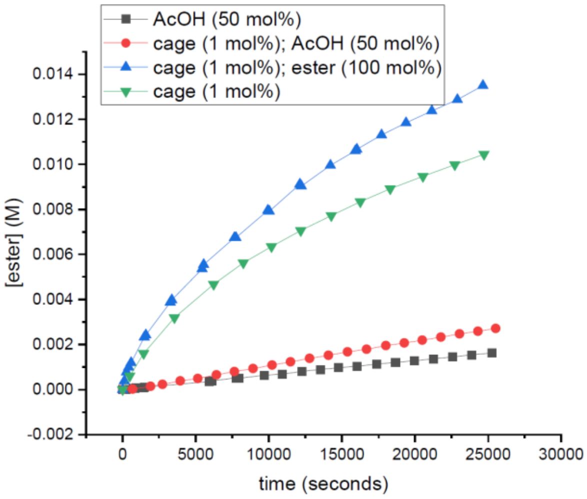

Figure S22. Addition of 50 mol% AcOH to cage **1** promoted esterifications inhibits the rate of catalysis. Addition of 100 mol% ester **7** to cage **1** promoted esterifications enhances the rate of catalysis slightly. The cage-promoted reactions are not background corrected in this graph.

Formation and availability of **$1Ac_1$** is not limiting under these conditions, although the equilibrium concentration of **$1Ac_1$** is reduced by ~20%, presumably due to reformation of Ac_2O by reaction with excess AcOH (as evidenced in **Figure S16**). The rate of catalysis in standard esterification reactions therefore decreases as the reaction progresses due to the build-up of acetic acid.

Use of chloroform containing HCl completely inhibits catalysis (*including cage acetylation)*, so care must be taken to use fresh $CDCl_3$, stored in the dark, over molecular sieves and potassium carbonate.

Attempt to observe transesterification with propionic acid

Enzymes can catalyze the backwards reaction as a well as the forwards reaction, which follows from the principle of microscopic reversibility. In practice, the rate of the reverse reaction might not be observed if the backwards barrier is too large. If the ester were breaking back into alcohol and cage-anhydride (**1Ac$_1$**), then the presence of a different carboxylic acid or alcohol would lead to observation of a crossover (transesterification) product. In order to reduce the possibility of observing background transesterification, we chose to use propionic acid in the control reaction. As observed for the anhydride exchange reactions (**Figures S16-19**, any formation of cage-acetyl-anhydride from the acetate ester would result in mixed anhydride equilibria, and therefore the formation of propionic-cage-anhydride, and also propionic ester.

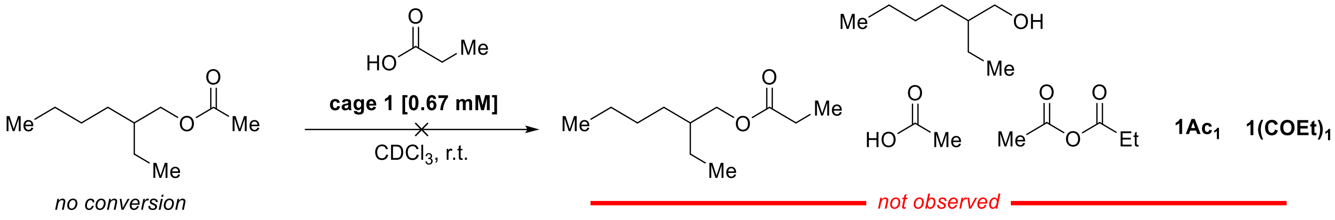

In practice, the standard catalysis conditions were performed in $CDCl_3$ with cage (0.67 mM), ethyl-2-hexyl acetate (66.8 mM), and propionic acid (66.8 mM) at 298 K. The reaction was monitored for 34 h by ^{1}H-NMR, but no evidence of acyl transfer to either the new ester, anhydride, or any cage anhydride species was observed.

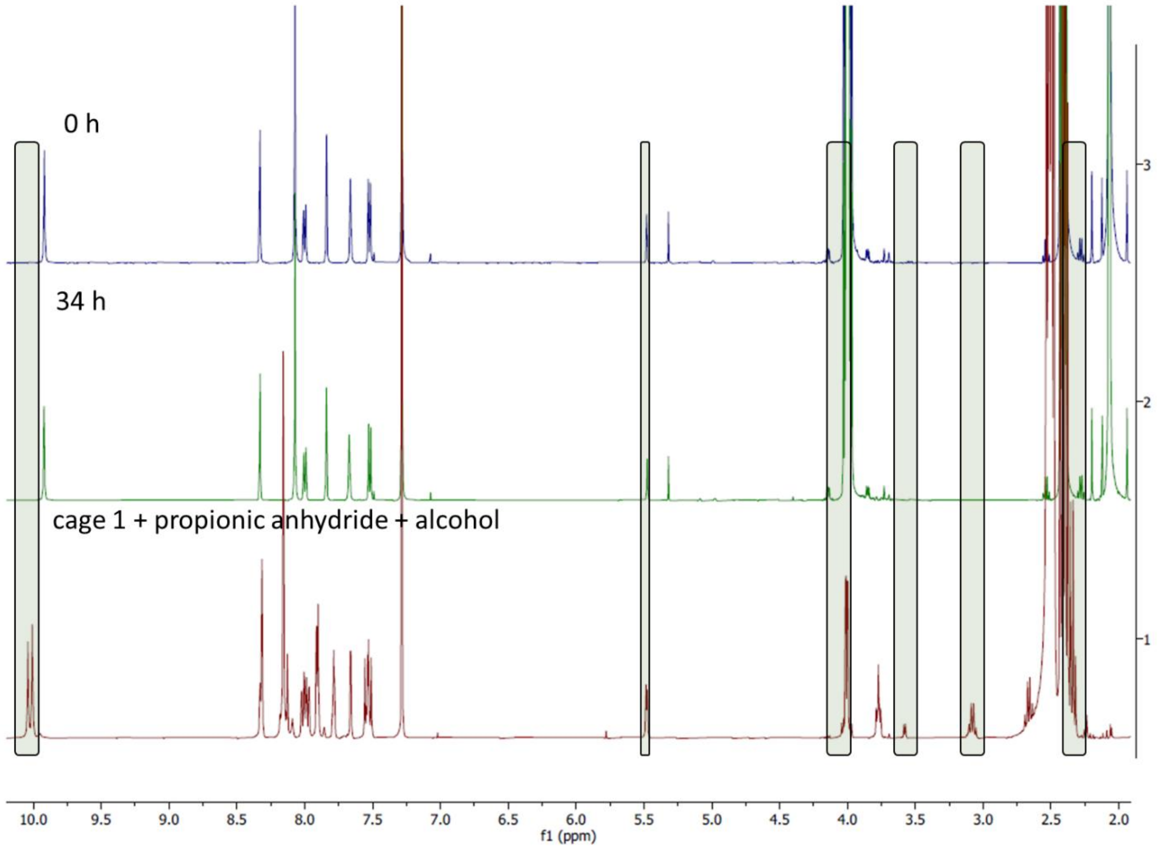

Figure S23. No transesterification of ester product is observed in the presence of cage **1**. No scrambling of ester groups (acetic/propionic) under catalysis conditions suggests the backwards acyl transfer is slow at best.

Addition of pyridine to cage 1-catalyzed reactions

Pyridine was used as an additive to probe inhibition or enhancement effects on cage promoted esterification catalysis of acetic anhydride with 2-ethylhexanol. Addition of pyridine (50 mol%) to an esterification reaction with cage **1** (2.5 mol%) increases the initial cage-catalyzed rate by an additional ~2.4 times (after background corrections) (**Figure S24**).

Method: A solution of cage **1** in $CDCl_3$ (0.698 mM) was weighed (0.550 mL, 0.825 g) into an NMR tube (0.384 µmol of cage, 1 mol%). Added was 2-ethylhexanol (6.00 µL, 38.4 µmol), the chosen additive, and acetic anhydride (4.28 µL, 38.4 µmol) and NMR spectra recorded over several time points. Conversion to ester against time was plotted. For the cage catalyzed reactions, the conversion was corrected for the background contribution.

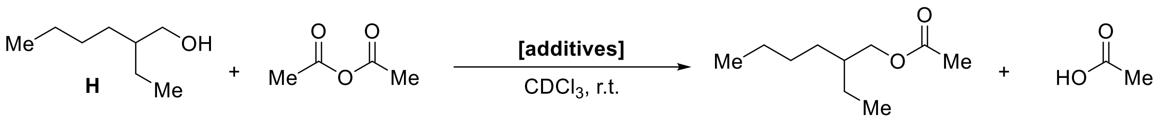

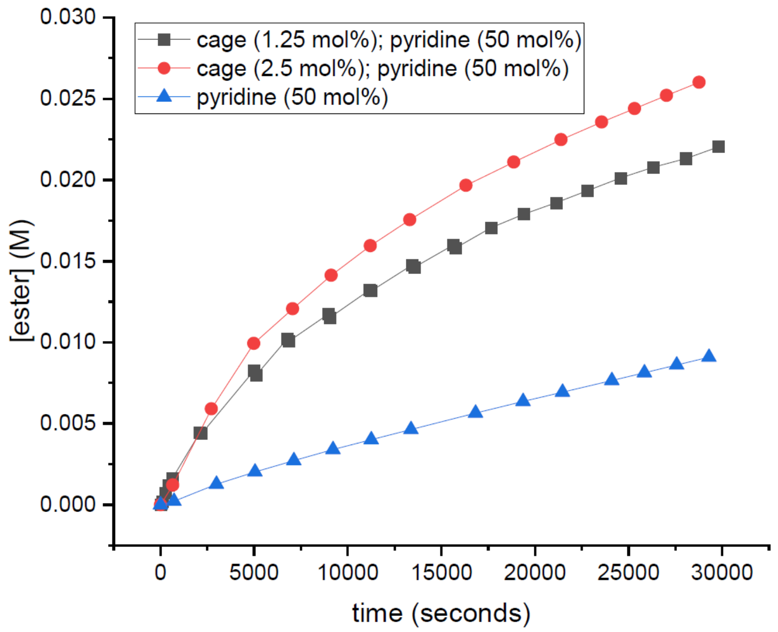

Figure S24. Pyridine enhances the catalytic properties of cage **1** in esterification reactions (catalytic profiles are corrected for background contributions).

Addition of pyridine to esterification reactions with cage 3 present leads to catalysis

Method: Using the standard kinetic method, the initial rates were measured by NMR in $CDCl_3$, with cage **3** (1.67 mM), pyridine (33.9 mM) (or as shown in the graph), 2-ethylhexanol (6.68 mM) and Ac_2O (159 mM). Conversion to ester against time were plotted after background correction (see **Figure S7** for data indicating cage **3** alone is not active).

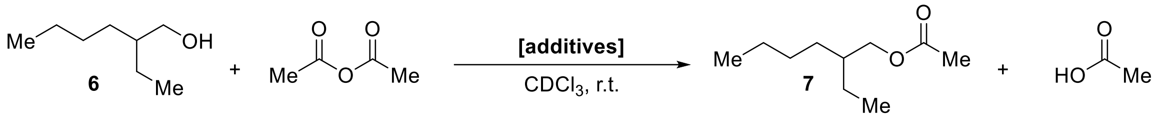

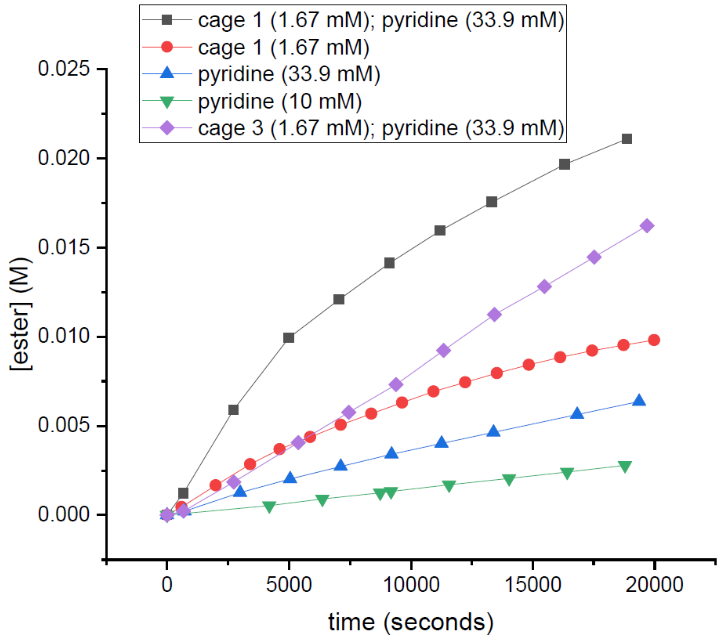

Figure S25. Addition of 50 mol% pyridine to inactive cage **3** leads to an active catalyst system, which is less susceptible to acid-inhibition. Addition of 50 mol% pyridine to cage **1**-promoted esterification reactions increases the rate of catalysis. The cage-promoted reactions are background corrected.

Reaction of cages **1,3,5** with acetic anhydride

Method: Solutions of cage (1.69 mM) in $CDCl_3$ were weighed into NMR tubes (0.550 mL, 0.825 g). Added was acetic anhydride (159 mM) and ^{1}H-NMR spectrum recorded immediately.

For cage 1 and Ac_2O, the fastest recorded spectrum was recorded within 30 seconds of addition of Ac_2O at 298 K to a pre-shimmed NMR tube, and showed full conversion to the monoacetyl cage **$1Ac_1$**.

Assuming a rate equation of: $$\frac{d[1Ac_1]}{dt} = k[cage][Ac_2O] \qquad \text{(SE10)}$$

The estimated minimum rate constant for cage acylation is therefore: $k > 0.21$ M^{-1} s^{-1}

Using cage **1** (0.67 mM) and Ac_2O (80 mM), the lower limit of k was: $k > 0.42$ M^{-1} s^{-1}.

For monoacid cage 5 + Ac_2O

The approximate second order rate constant for cage **5** acylation is: $k = 1.6 \times 10^{-4}$ M^{-1} s^{-1}

$$\frac{d[5Ac_1]}{dt} = -\frac{d[cage\ \mathbf{5}]}{dt} = k[cage\ \mathbf{5}][Ac_2O]$$

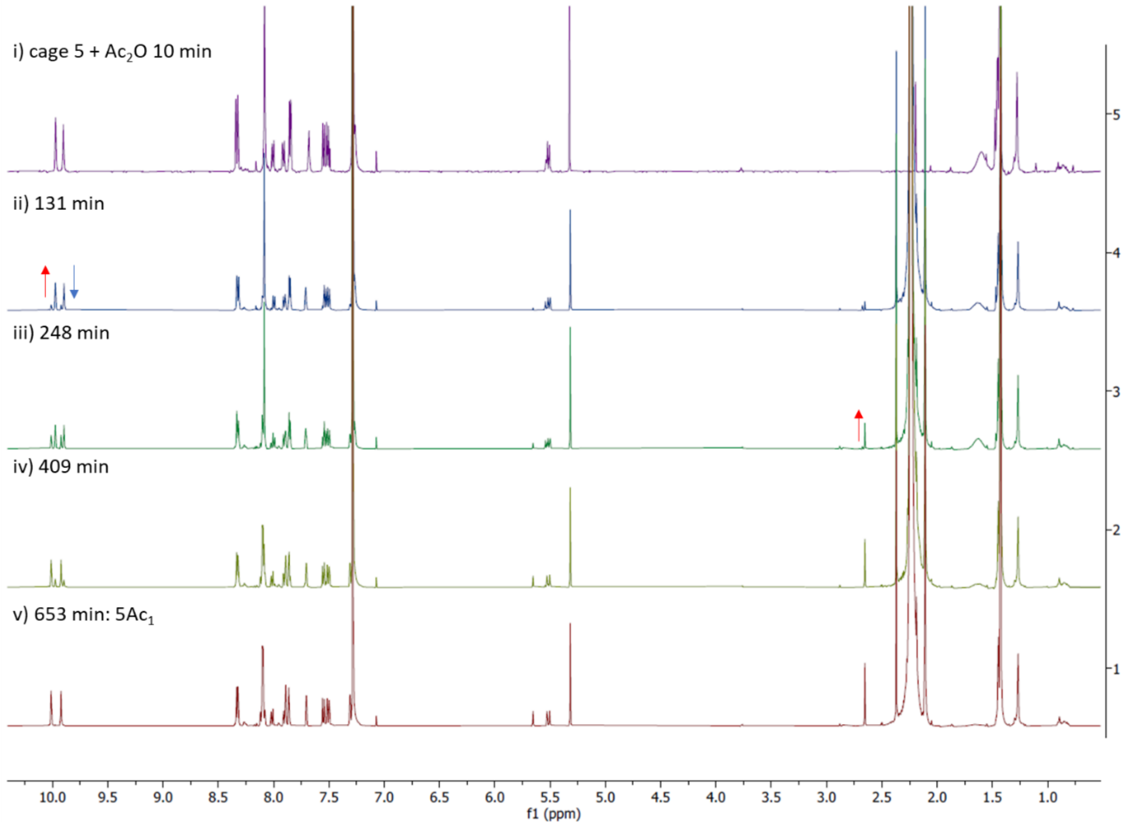

Figure S26. Cage **5** + Ac_2O. The slow formation of **$5Ac_1$** is noted with red arrows.

$5Ac_1$: ^{1}H NMR (500 MHz, $CDCl_3$) δ 10.01 (s, 3H), 9.92 (s, 3H), 8.34 (d, J = 1.7 Hz, 3H), 8.32 (d, J = 1.7 Hz, 3H), 8.13 – 8.06 (m, 12H), 8.02 (dd, J = 8.1, 2.1 Hz, 3H), 7.90 (dd, J = 8.1, 2.1 Hz, 3H), 7.89 (d, J = 1.8 Hz, 3H), 7.86 (d, J = 1.8 Hz, 3H), 7.70 (d, J = 2.1 Hz, 3H), 7.55 (d, J = 8.1 Hz, 3H), 7.51 (d, J = 8.1 Hz, 3H), 7.31 (d, J = 2.1 Hz, 3H), 5.65 (s, 1H), 5.53 (s, 1H), 5.50 (s, 1H), 2.65 (s, 3H), 1.43 (s, 27H), 1.42 (s, 27H).

Diacid (non-pyridine) cage 3 + Ac_2O

The approximate second order rate constant for acylation of cage **3** with Ac_2O is: $k = 8.8 \times 10^{-3}\ M^{-1}\ s^{-1}$

$$\frac{d[3Ac_1]}{dt} = -\frac{d[\mathbf{3}]}{dt} = k[cage\ \mathbf{3}][Ac_2O]$$

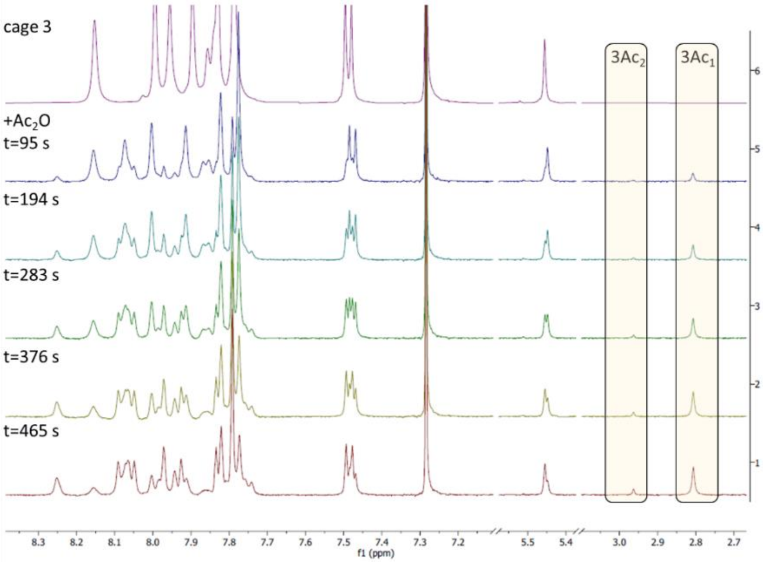

Figure S27. Cage **3** + Ac_2O. The rapid formation of **$3Ac_1$** and the slower formation of **$3Ac_2$** are highlighted.

When pyridine (10 mM, 6 equivalents relative to cage **3**) is present, the rate of formation of **$3Ac_1$** is approximately: k = ~$6.2 \times 10^{-2}\ M^{-1}\ s^{-1}$ (measured as the consumption of **3**). **$3Ac_2$** is formed more significantly too.

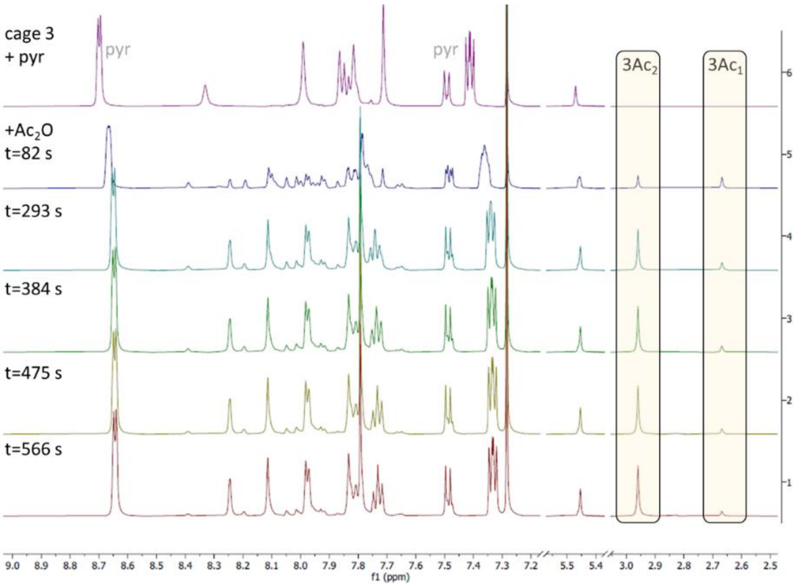

Figure S28. Cage **3** + Ac_2O + pyridine (6 equiv wrt cage).

Direct comparison of esterification catalysis rate using cage **1** or [cage **3** + 6 equiv pyr]

Using the standard kinetic method, the initial rates were measured by NMR in $CDCl_3$, with cage **3** (1.69 mM), pyridine (10 mM, 6 equiv wrt cage), 2-ethylhexanol (6.68 mM) and Ac_2O (159 mM). Conversion to ester against time was plotted after background correction.

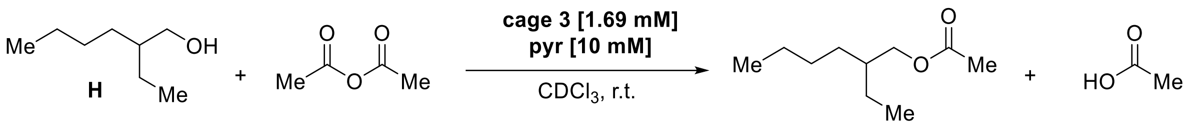

	run A	run B	run C	average	error
initial rate *(M s⁻¹)*	5.05E-08	5.26E-08	5.02E-08	5.11E-08	3%
k $(M^{-1}s^{-1})$	4.53E-03	4.71E-03	4.50E-03	4.58E-03	3%
rate enhancement	5.69	5.94	5.70	5.78E+00	3%

Initial rate = *k*[cage **3**][alcohol]; the initial rates are background corrected using the (initial) rate constant for the background in the presence of pyridine (10 mM). All measurements at 298 K. Error is the standard error at 95% confidence intervals from 3 measurements.

As shown in the table, the absolute (initial) rate enhancement of ester formation compared to background with just pyridine (10.0 mM) under these conditions is a **factor of ~6**.

Second order rate constant ratio: $k_{cage3+pyr} / k_{bg} = 4.58 \times 10^{-3}\ M^{-1}s^{-1} / 9.36 \times 10^{-6}\ M^{-1}s^{-1} = 489$

The rate constant for cage **3** + 6 equiv free pyridine is 32 times smaller than for pyridine cage **1** (with no external pyridine).

Second order rate constant ratio: $k_{cage1} / k_{cage3+pyr} = 1.44 \times 10^{-1}\ M^{-1}s^{-1} / 4.58 \times 10^{-3}\ M^{-1}s^{-1} = 31.9$

These rate constants all assume [cage] is representative of the active cage species.

Initial measured rates with cage **5** (1.67 mM) are no better than background. Therefore, **Figure 5b** in the manuscript shows the rate constant calculated using the same equation as background, SE1.

	run A	run B	run C	average	error
initial rate *(M s⁻¹)*	9.81E-09	9.36E-09	9.49E-09	9.55E-09	3%

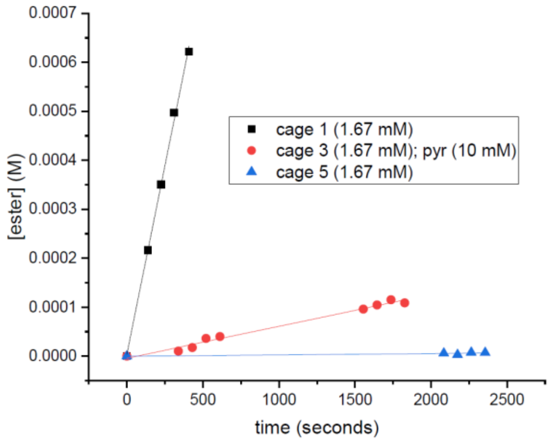

Figure S29. Comparison of initial rates of esterification catalyzed by cage **1**, cage **5**, and cage **3**+pyr under standardized conditions.

Eyring Analysis

Pseudo first order conditions were used to obtain initial rate data.

Background

Method: From stock solutions, 2-ethylhexanol (3.83 µmol in 10 µL) and pyridine (5.76 µmol in 6 µL) were dissolved in $CDCl_3$ (0.550 mL, 0.825 g) in an NMR tube, and the solution mixed with vigorous shaking. The sample was inserted into an NMR probe (500 MHz) and the sample locked and shimmed and an initial proton spectrum recorded. The NMR tube was temperature-equilibrated in the NMR probe at the appropriate temperature for 20 min, and then removed briefly to add acetic anhydride (8.56 µL, 76.8 µmol) and a timer started. The tube was quickly returned, and conversion to ester against time monitored by ^{1}H-NMR spectra, and a linear fit of the initial points (14-26 data points) up to 1% conversion made. The rate data were fit and processed as for the SAR study. The effective second order rate constant, k_{eff}, was obtained using SE1.

Table S4. Second order rate constants for the background esterification reaction at different temperatures.

	k_{eff} / M^{-1} s^{-1}				
**temp / K**	**run A**	**run B**	**run C**	**average *k***	**error**
293	7.91E-06	1.16E-05	1.05E-05	1.00E-05	21%
298	1.63E-05	1.10E-05	9.98E-06	1.24E-05	30%
303	1.37E-05	1.73E-05	1.89E-05	1.66E-05	18%
308	3.47E-05	3.33E-05	2.89E-05	3.23E-05	10%
313	3.00E-05	4.80E-05	4.14E-05	3.98E-05	25%

$k_{eff} = v_i$ / ([Ac_2O][alc]); where k_{eff} is the effective second order rate constant, v_i is the initial rate, and [Ac_2O] and [alc] are the initial concentrations of acetic anhydride and alcohol, respectively; standard error quoted at 95% confidence intervals for three repeats.

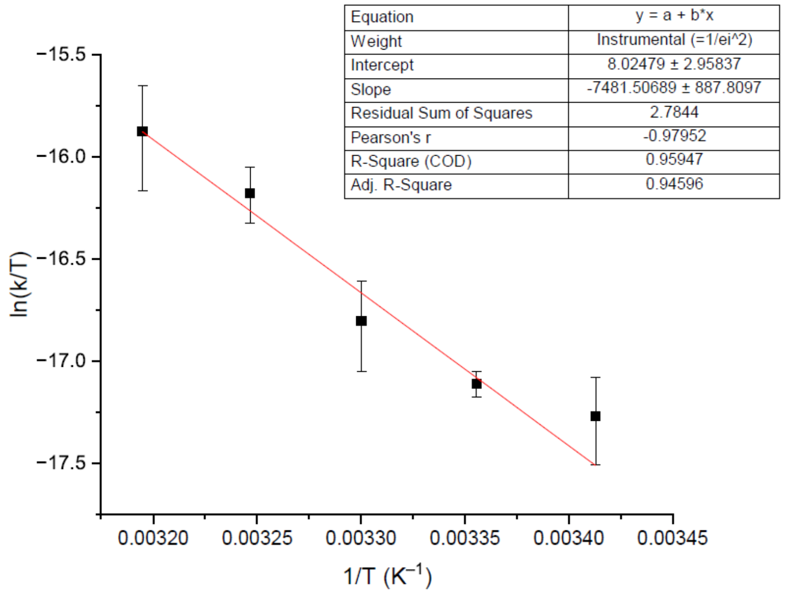

Figure S30. Eyring plot for the background catalysis reaction at 293, 298, 303, 308 and 313 K.

Catalysis

Method: From a stock solution, cage **1** (0.96 µmol) in $CDCl_3$ (0.550 mL, 0.825 g) was weighed into an NMR tube. From a $CDCl_3$ stock solution, was added 2-ethylhexanol (3.83 µmol in 10 µL). The NMR tube was temperature-equilibrated in an NMR probe at the appropriate temperature for 20 min, and removed briefly to add acetic anhydride (8.56 µL, 76.8 µmol). The tube was quickly returned, and conversion to ester against time monitored by ^{1}H-NMR spectra. The data was fit and processed as for the SAR study, including background correction using the rate constants measured above. The effective second order rate constant, k_{cage}, was obtained using SE7.

Table S5. Second order rate constants for the cage-catalyzed esterification reaction at 293, 298, 303, 308, 313 K.

		k / M^{-1} s^{-1}			
**temp / K**	**run A**	**run B**	**run C**	**average *k***	**error**
293	1.22E-01	1.37E-01	1.07E-01	1.22E-01	14%
298	1.55E-01	1.42E-01	1.34E-01	1.44E-01	8%
303	1.80E-01	1.83E-01	1.84E-01	1.82E-01	1%
308	2.25E-01	2.11E-01	2.09E-01	2.15E-01	5%
313	2.93E-01	2.60E-01	2.41E-01	2.65E-01	11%

$k = v_i$ / ([cage][alc]); where k is the effective second order rate constant, v_i is the initial rate, and [cage] and [alc] are the initial concentrations of cage and alcohol, respectively; standard error quoted at 95% confidence intervals for three repeats. We make the assumption that the active cage species (**1Ac$_1$**) is well approximated by using the initial concentration of cage.

Graphs of initial rates of catalyzed reactions at 293, 298, 303, 308, 313 K

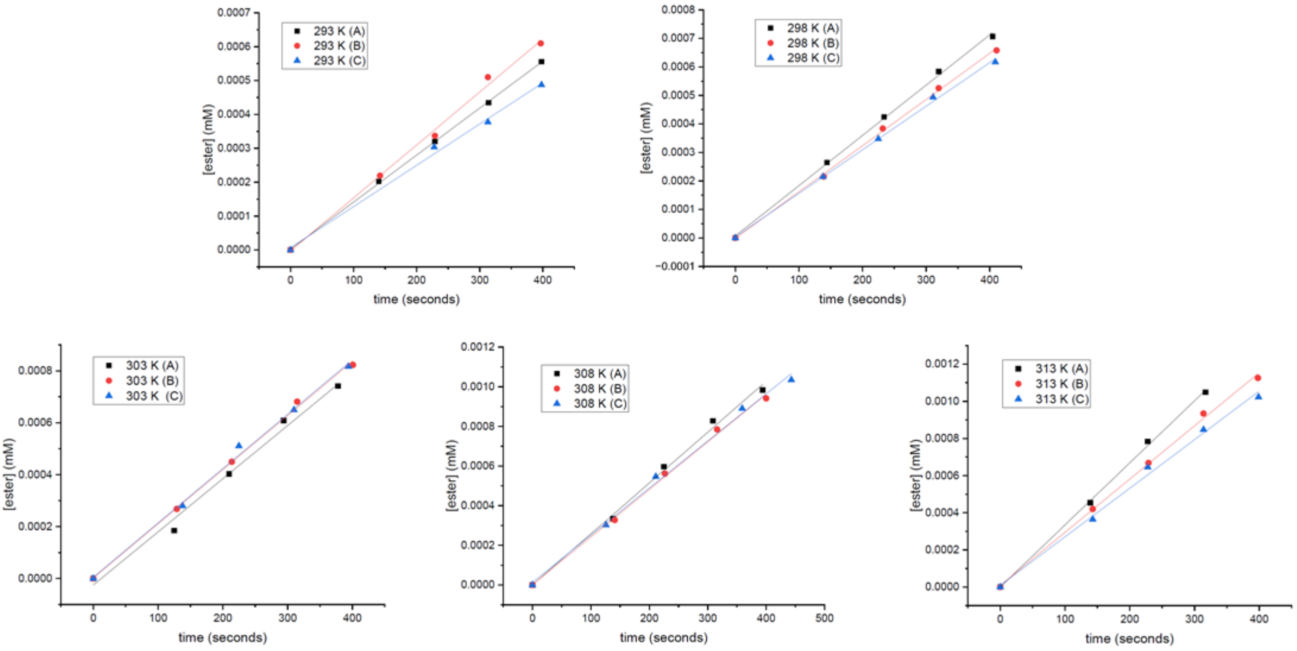

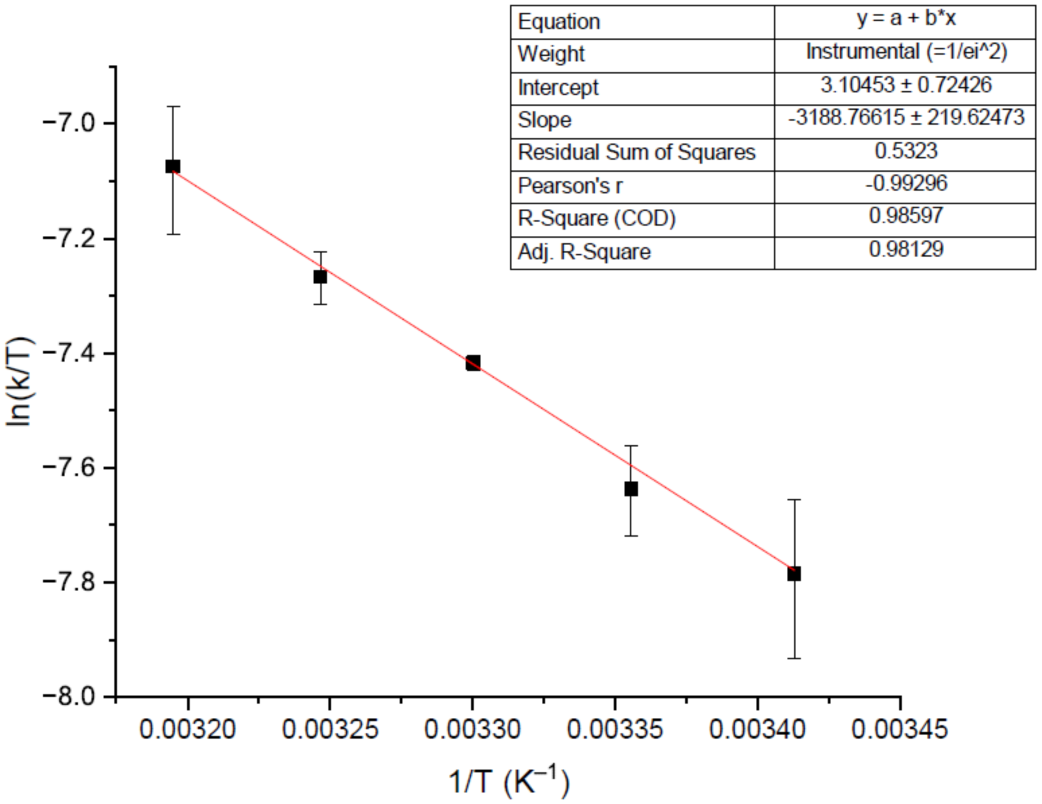

Figure S31. Eyring plot for the cage-catalyzed esterification reaction at 293, 298, 303, 308 and 313 K.

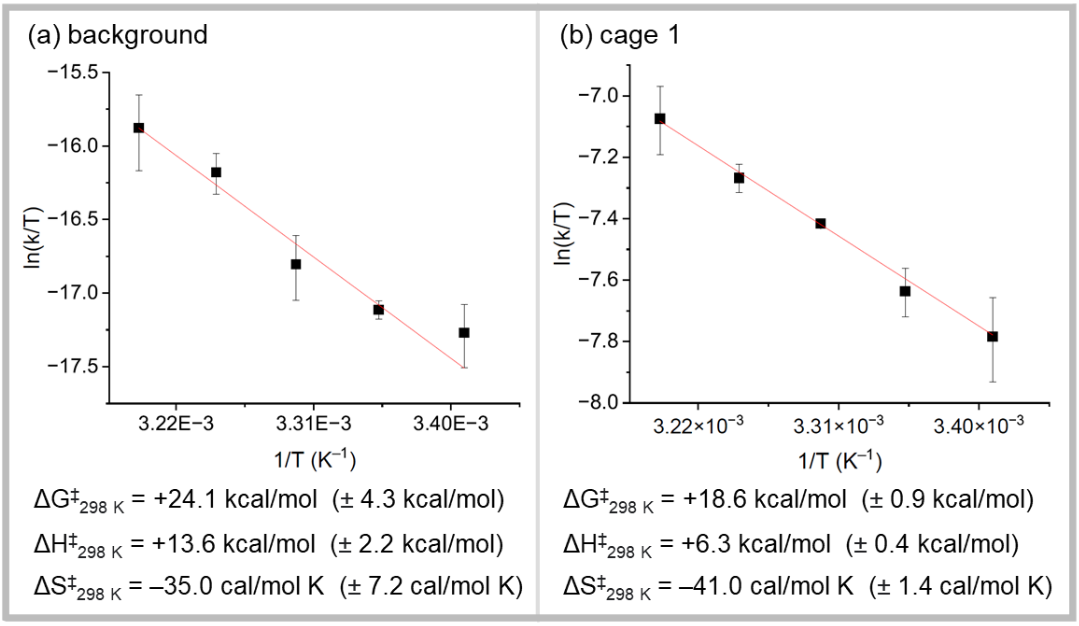

Figure S32. Eyring analysis summary. The background reaction has an activation free energy barrier ΔG‡(298 K) = +100.8 kJ/mol, with ΔH‡ = +57.1 kJ/mol and ΔS‡ = –146.6 J/mol K. The catalyzed reaction has ΔG‡(298 K) = +77.7 kJ/mol, with ΔH‡ = +26.5 kJ/mol and ΔS‡ = –171.7 J/mol K.

Estimation of transition state thermodynamic parameters from Eyring analysis

Using the Eyring equation, the activation Gibbs free energy barrier and estimations of the thermodynamic quantities contributing to it can be obtained.

$$k = \kappa \frac{k_B T}{h} e^{\frac{-\Delta G^{\ddagger}}{RT}} \qquad \text{(SE11)}$$

where k is the measured rate constant, κ is the transmission coefficient, assumed here to be 1 (not necessarily true), k_B is the Boltzmann constant, T is the temperature of the measurement, h is Planck's constant, $\Delta G^{\ddagger}$ is the Gibbs free energy of activation, and R is the ideal gas constant.

The linearized form (SE12) was used to plot the data, and extract $\Delta H^{\ddagger}$ and $\Delta S^{\ddagger}$.

$$ln\frac{k}{T} = \frac{-\Delta H^{\ddagger}}{R}\frac{1}{T} + ln\frac{\kappa k_B}{h} + \frac{\Delta S^{\ddagger}}{R} \qquad \text{(SE12)}$$

At 298 K, this gave for the cage-promoted reaction:

Slope: -3188.77 (+/– 219.625)

Intercept: 3.10453 (+/– 0.72426)

$\Delta G^{\ddagger}_{298\ K}$ = +77.7 kJ/mol (+/– 3.6 kJ/mol)

$\Delta H^{\ddagger}_{298\ K}$ = +26.5 kJ/mol (+/– 1.8 kJ/mol)

$\Delta S^{\ddagger}_{298\ K}$ = –171.7 J/mol K (+/– 6.0 J/mol K)

At 298 K, this gave for the background reaction:

Slope: -6862.27 (+/– 1097.31)

Intercept: 6.12888 (+/– 3.60975)

$\Delta G^{\ddagger}_{298\ K}$ = +100.7 kJ/mol (+/– 18.1 kJ/mol)

$\Delta H^{\ddagger}_{298\ K}$ = +57.1 kJ/mol (+/– 9.1 kJ/mol)

$\Delta S^{\ddagger}_{298\ K}$ = –146.6 J/mol K (+/– 30.0 J/mol K)

Error bars on the Eyring plot are set using the confidence intervals at 95% over 3 runs. The linear fit of the linearized Eyring plot uses "instrumental" weighting, which gives more weighting to the points with a lower error, given by the reciprocal of the square of the error (which is defined using the error bars). The slope and intercept values changed by less than the error when no weighting was used.

Computational Modelling

Molecular dynamics

Parametrization. For consistency, parametrizations were done following the procedure from our previous work.[9,12,13] The supramolecular cage and substrate were parametrized using Antechamber with AMBER force-field.[14] The partial charges were assigned using restrained electrostatic potential (RESP) obtained from Gaussian 16[15] on HF/6-31G*//B3LYP/6-31G* level of theory. The parameters of the chloroform solvent were obtained from virtualchemistry.org.[16]

Simulations. Molecular dynamics simulations were performed with Gromacs 2021.3.[17,18] The cage (and other reactants) were placed into a 5 nm × 5 nm × 5 nm cubic box with 500 chloroform molecules. The systems were energy minimized. Subsequently, they were equilibrated in an NVT ensemble using a v-rescale thermostat (298 K; τ=0.1 ps) for 20 ps and an NPT ensemble using a c-rescale barostat[19] (1 atm; τ=2 ps) for 20 ps. Production runs were done with v-rescale thermostats and c-rescale barostats.[20] All calculations were done using a 2 fs time step.

Density functional theory (DFT)

All DFT calculations were performed with ORCA 5.0.3, except for the RESP calculation (see above). The structures were firstly optimized with xTB,[21,22] followed by optimization with PBE0 functional[23], with Ahlrichs' def2-SVP basis set[24] and damped D3 dispersion correction (D3BJ).[25,26] The solvent effects were included using the conductor-like polarisable continuum model (CPCM).[27] Thermal contributions at 298 K were calculated using Grimme's quasi-rigid-rotor-harmonic oscillator on the same level of theory.[28] The single-point calculations were done with M06-2X functional[29] with a def2-TZVP basis set. This level of theory was validated by comparing the results of the uncatalyzed reaction computed at CPCM(CHCl3)-DLPNO-CCSD(T)/def2-TZVPP level of theory. For the background reaction, the transition state (TS) was located using *autode*.[30] For the catalyzed reaction, the initial guess of the TS was identified by the growth string method with xTB,[31,32] followed by optimization to the TS using ORCA.

Conformational Study

MD calculations

To search accessible conformers, we generated cages with all possible configurations and performed 13×200 ns MD simulations for each as previously described.[9] Additionally, we performed 4 extended simulations (1 µs each) starting from conformer **C5**. The trajectories were analyzed by categorizing the cage into one of 13 possible conformers. The orientations of the amides were considered 'outward' if the distance between the amide carbonyl oxygen atoms and the external ortho-triptycene proton (H7) was below 3.6 Å. All the amide orientations were converted into "Cage ID" values, which were sorted alphabetically to ensure cages were symmetry normalized for comparison (first within each pair of edge carbonyls, retaining top/bottom information across the three edges; then reordering the edges alphabetically, which is possible due to the cage symmetry). The data shows that **C13** is favored, with **C12**, **C9**, **C5**, **C10** and **C6** accessible.

QM calculations

All possible cage conformations were generated after conformational sampling[9] and optimized using DFT. In line with the MD simulations, the conformer with all amide oxygens facing outward (**C13**) shows the lowest energy (**Figure S33**, **Table S6**). Furthermore, the optimized structures display significant differences in the cavity size, which can be estimated from the distance between carbons of both carboxylic groups, C_{COOH}-C_{COOH}. This distance correlates with the number of oxygen amides facing inwards (i.e., more amides pointing inwards results in a larger cavity) and ranges from 6.6 Å for the smallest one (with all amide oxygen pointing outwards) to 10.0 Å for the largest one (with all amide oxygen pointing inwards).

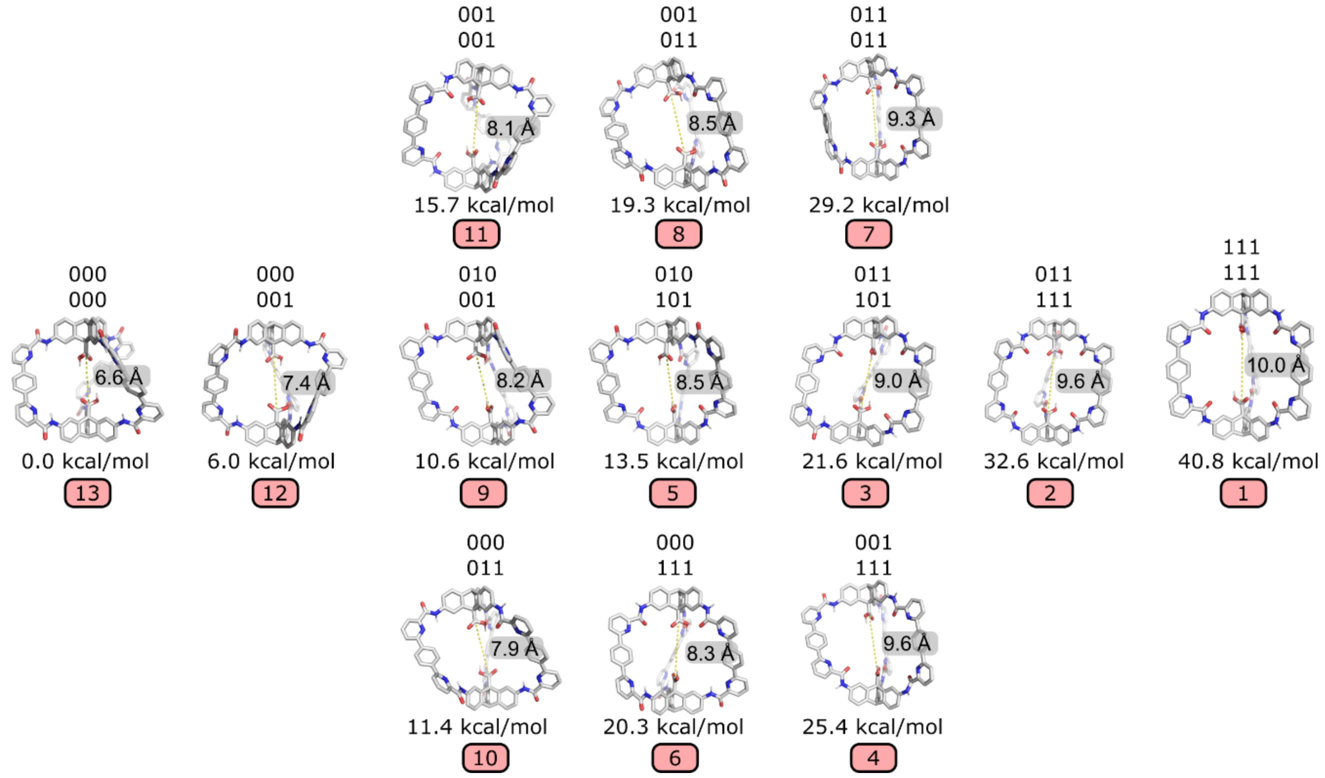

Figure S33. Landscape of available conformational space for cage **1**. Energies calculated CPCM($CHCl_3$)-M06-2X/def2-TZVP//D3BJ-CPCM($CHCl_3$)-PBE0/def2-SVP level of theory. The yellow dotted line represents the $\mathbf{C}_{COOH}$-$\mathbf{C}_{COOH}$ distance (r_{cc}).

Table S6. Single point energies of the optimized cage **1** conformers.

	D3BJ-CPCM($CHCl_3$)-PBE0/def2-SVP//D3BJ-CPCM($CHCl_3$)-PBE0/def2-SVP		CPCM($CHCl_3$)-M06-2X/def2-TZVP//D3BJ-CPCM($CHCl_3$)-PBE0/def2-SVP	
	E [Eh]	ΔE [kcal/mol]	E [Eh]	ΔE [kcal/mol]
**C1**	−5093.5932	38.1	−5102.6124	40.8
**C2**	−5093.6033	31.8	−5102.6255	32.6
**C3**	−5093.6223	19.9	−5102.6431	21.6
**C4**	−5093.6133	25.5	−5102.6370	25.4
**C5**	−5093.6356	11.5	−5102.6560	13.5
**C6**	−5093.6227	19.6	−5102.6451	20.3
**C7**	−5093.6102	27.4	−5102.6309	29.2
**C8**	−5093.6211	20.6	−5102.6467	19.3
**C9**	−5093.6385	9.7	−5102.6605	10.6
**C10**	−5093.6374	10.4	−5102.6593	11.4
**C11**	−5093.6291	15.6	−5102.6525	15.7
**C12**	−5093.6452	5.5	−5102.6680	6.0
**C13**	−5093.6539	0.0	−5102.6775	0.0

Table S7. Boltzmann weighted (including degeneracy) populations for the conformers of cage **1**.

ID	degeneracy	population
**C1**	1	0.0%
**C2**	6	0.0%
**C3**	6	0.0%
**C4**	6	0.0%
**C5**	6	0.0%
**C6**	2	0.0%
**C7**	3	0.0%
**C8**	12	0.0%
**C9**	6	0.0%
**C10**	6	0.0%
**C11**	3	0.0%
**C12**	6	0.0%
**C13**	1	100.0%

(SPE//geometry optimization)
CPCM(CHCl3)-M06-2X/def2-TZVP//D3BJ-CPCM(CHCl3)-PBE0/def2-SVP

Acetylation of the cage

MD: Analysis of 1 µs MD simulation of the **$1Ac_1$** cage in chloroform showed that the cage exclusively exists in the configuration where all amide oxygens point outwards (**C13**). The sole presence of **$1Ac_1$** in this configuration is a result of the hydrogen bond formed between the carboxylic acid group and the acyl oxygen (**Figure S34, Figure 3a**), which forces the cage to adopt a compact structure. Cage **$1Ac_1$** (**C13**) has a C_{COOH}-C_{COOH} distance of 5.7 Å, 0.9 Å shorter than the most compact unacetylated cage **1** conformer (**C13**).

DFT: The computed reaction energy for the transformation of cage **1** into **$1Ac_1$** and **$1Ac_2$** shows that the three species have similar energies in solution, with **$1Ac_1$** slightly favored (**Figure 34**; **Table 8**). The small energy stabilization for **$1Ac_1$** compared to **1** or **$1Ac_2$** suggests that formation of the hydrogen bond in **$1Ac_1$** may be offset by a more strained cage conformation. Indeed, constraining cage **1** to a C_{COOH}–C_{COOH} distance of 5.7 Å (as observed in **$1Ac_1$**) results in an energy increase of 2.1 kcal/mol with respect to unconstrainted cage **1** (and 3.6 kcal/mol in respect to **$1Ac_1$**). These findings suggest that the hydrogen bond in **$1Ac_1$** is not at an ideal length in the ground state.

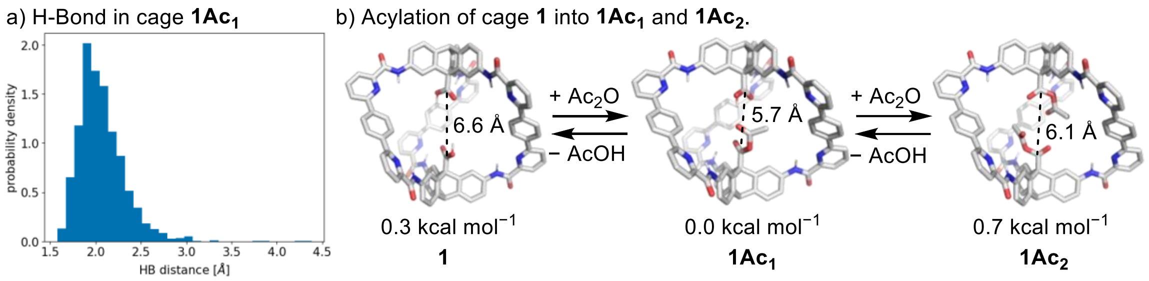

Figure S34. Cage acetylation calculations. (a) The distance distribution between acyl's oxygen and carboxylic acid hydrogen from 1 µs of MD with cage **$1Ac_1$**. (b) Computed reaction energies for acylation at the CPCM($CHCl_3$)-M06-2X/def2-TZVP//D3BJ-CPCM($CHCl_3$)-PBE0/def2-SVP level of theory.

Table S8. Single point energies and thermal contributions for acylation reaction of cage **1** into **$1Ac_1$** and **$1Ac_2$**.

	D3BJ-CPCM(CHCl3)-PBE0/def2-SVP//D3BJ-CPCM(CHCl3)-PBE0/def2-SVP				CPCM(CHCl3)-M06-2X/def2-TZVP//D3BJ-CPCM(CHCl3)-PBE0/def2-SVP				
	E[Eh]	G-E[Eh]	H-E[Eh]	Freq	E [Eh]	ΔE [kcal/mol]	ΔG [kcal/mol]	ΔH [kcal/mol]	−TΔS [kcal/mol]
**Ac_2O**	-381.0442	0.0699	0.1075	None	-381.7395				
**AcOH**	-228.6724	0.0381	0.0674	None	-229.0973				
**1**	-5093.6539	1.2502	1.4448	None	-5102.6775	1.5	0.3	1.6	-1.4
**$1Ac_1$**	-5246.0311	1.2840	1.4847	None	-5255.3221	0.0	0.0	0.0	0.0
**$1Ac_2$**	-5398.3978	1.3154	1.5249	None	-5407.9629	0.9	0.7	0.9	-0.3
**1** (constrained to 5.7 Å)	-5093.652				-5102.6741	3.6			

Binding of alcohol in cage **1** and cage **1Ac$_1$**

A series of 1 µs simulations of cage **1** with varying alcohol ratios (cage: alcohol at 1:1, 1:2, 1:4, 1:8, 1:16, 1:32) showed that the centre of mass (COM) of bound alcohol is within 6 Å from the COM of cage **1** (**Figure S35a**). Using 6 Å as the cut-off distance for bound alcohol, the cage reached saturation at a ratio of 16:1 (**Figure S35b**), accommodating two alcohol molecules forming a hydrogen bond network connecting the carboxylic acids (**Figure S35c**).

In contrast to cage **1**, 1 µs simulations of **1Ac$_1$** showed weak alcohol binding with 0-1 bound molecules at the saturation limit (16:1 ratio). Further analysis of the hydrogen bond network between alcohol and the acidic/ester groups revealed three distinctive binding modes (A, B, C), for which representative frames were extracted and optimized using DFT (**Figure S35d**; **Table S9**).

DFT calculations of the complexes show that **1Ac$_1$** and complex A can exist in equilibrium as the reaction energy is close to zero (<0.1 kcal/mol). Cages **1**, **1Ac$_1$**, **1Ac$_2$** and complex **6⊂1Ac$_1$** have similar Gibbs energies (<1 kcal/mol) and are expected to co-exist in solution at equilibrium with a ratio of 0.23:0.33:0.10:0.35 (obtained from Boltzmann weightings).

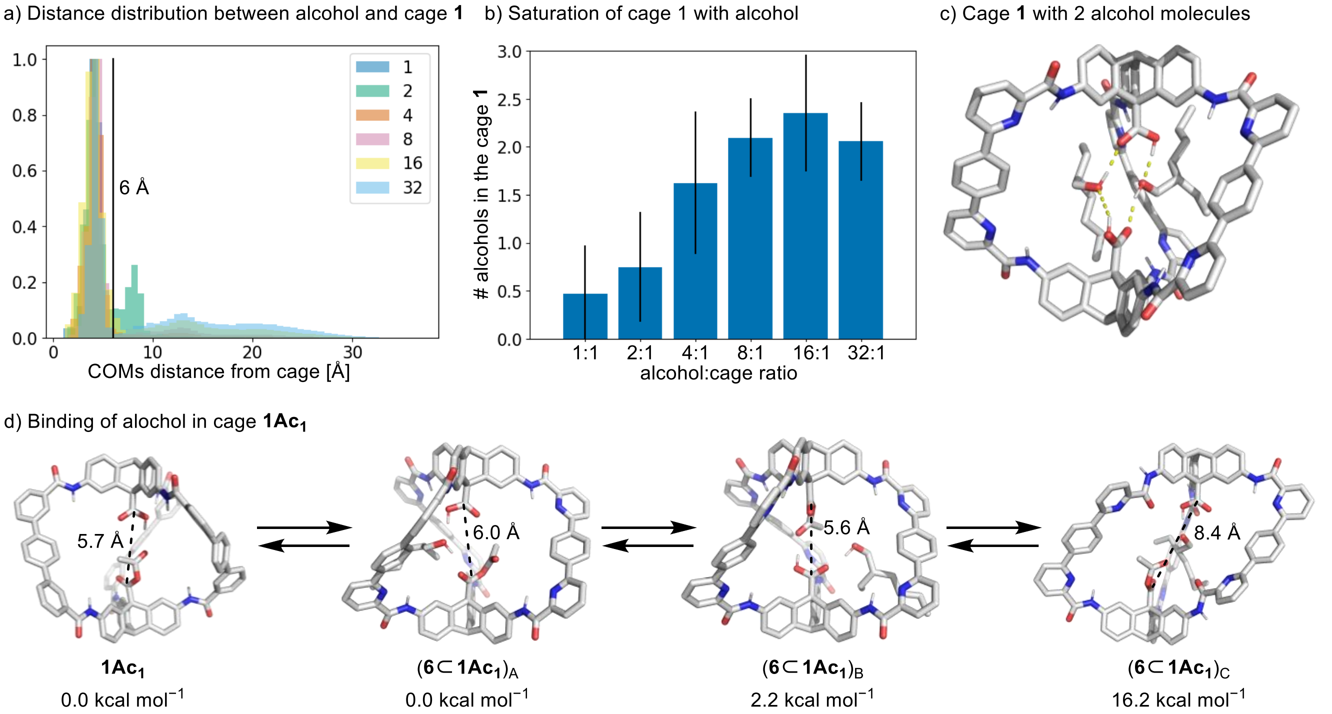

Figure S35. Alcohol binding in cage **1** and **1Ac$_1$**. (a) Distribution of distances between COMs of the cage and alcohols from 1 µs simulation. (b) Number of alcohols in cage 1 for simulations with varying alcohol:cage ratios. (c) Snapshot of the two alcohol molecules bound in cage **1**. (d) Binding of the alcohol in cage **1Ac$_1$** with different hydrogen bond networks extracted from MD and optimized at CPCM($CHCl_3$)-M06-2X/def2-TZVP//D3BJ-CPCM($CHCl_3$)-PBE0/def2-SVP level of theory.

Table S9. Single point energies and thermal contributions for alcohol binding in cage **1Ac₁**.

	D3BJ-CPCM(CHCl3)-PBE0/def2-SVP//D3BJ-CPCM(CHCl3)-PBE0/def2-SVP				CPCM(CHCl3)-M06-2X/def2-TZVP//D3BJ-CPCM(CHCl3)-PBE0/def2-SVP						
	E [Eh]	G-E [Eh]	H-E [Eh]	im. Freq [cm^{-1}]	E [Eh]	ΔE [kcal/mol]	ΔG [kcal/mol]	ΔH [kcal/mol]	−TΔS [kcal/mol]	Boltzmann factor	Probability
Ac_2O	-381.0442	0.0699	0.1075	None	-381.7395						
AcOH	-228.6775	0.0382	0.0675	None	-229.0973						
**6**	-390.1930	0.2167	0.2638	None	-390.8747						
**1**	-5093.6539	1.2502	1.4448	None	-5102.6775	6.5	0.1	5.9	-5.8	0.83	0.23
**1Ac$_1$**	-5246.0311	1.2840	1.4847	None	-5255.3221	5.0	-0.1	4.3	-4.4	1.19	0.33
**1Ac$_2$**	-5398.3978	1.3154	1.5249	None	-5407.9629	5.9	0.6	5.3	-4.7	0.35	0.10
**(6⊂1Ac$_1$)$_A$**	-5636.2689	1.5253	1.7514	None	-5646.2215	-10.5	-0.1	-9.3	9.2	1.27	0.35
**(6⊂1Ac$_1$)$_B$**	-5636.2649	1.5265	1.7515	None	-5646.2192	-9.1	2.0	-7.9	9.9		
**(6⊂1Ac$_1$)$_C$**	-5636.2449	1.5259	1.7524	None	-5646.1963	5.3	16.0	7.0	9.0		
Effective Boltzmann reactant state						0.0	0.0	0.0	0.0		

Uncatalyzed (background) esterification reaction

We propose that the background reaction proceeds via a cyclic transition state in which the proton is transferred to the adjacent anhydride carbonyl group with a direct alcohol oxygen attack on the acyl carbon. The energy barrier has been calculated by optimizing with D3BJ-CPCM($CHCl_3$)-PBE0/def2-SVP level of theory and single point energies were calculated at CPCM($CHCl_3$)-M06-2X/def2-TZVP (**Figure S36**; **Table 10**). The accuracy of the single point calculations has been validated by CPCM($CHCl_3$)-DLPNO-CCSD(T)/def2-TZVPP showing good agreement for both methods (RMSE=1.02 kcal/mol; **Table S10**). We also assessed CPCM($CHCl_3$)-ωB97X-D3/def2-TZVP level of theory which performed slightly worse than M06-2X (RMSE=1.45 kcal/mol).

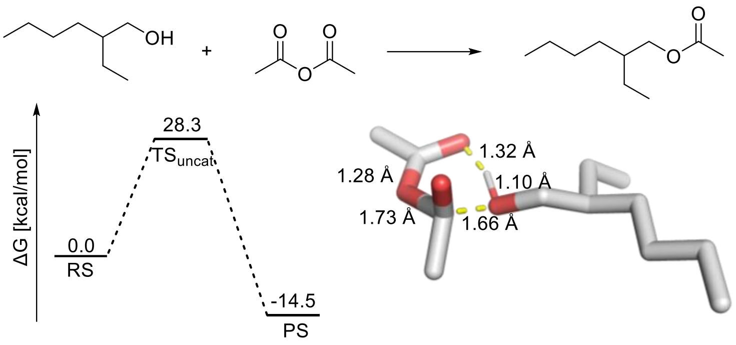

Figure S36. QM calculations of the uncatalyzed esterification. The thermal contributions were calculated at D3BJ-CPCM(CHC$_{l3}$)-PBE0/def2-SVP. Single point energy at CPCM($CHCl_3$)-M06-2X/def2-TZVP.

Table S10. Single point energies and thermal contributions for the uncatalyzed (background) reaction.

		D3BJ-CPCM(CHCl3)-PBE0/def2-SVP//D3BJ-CPCM(CHCl3)-PBE0/def2-SVP				CPCM(CHCl3)-M06-2X/def2-TZVP//D3BJ-CPCM(CHCl3)-PBE0/def2-SVP				
		E [Eh]	G-E [Eh]	H-E[Eh]	im. Freq [cm^{-1}]	E [Eh]	ΔE [kcal/mol]	ΔG [kcal/mol]	ΔH [kcal/mol]	–TΔS [kcal/mol]
RS	**6**	-390.1930	0.2063	0.2542	None	-390.8748				
	Ac_2O	-381.0442	0.0656	0.1038	None	-381.7395				
	6·Ac_2O	-771.2524	0.2899	0.3603	None	-772.6223	-5.0	6.3	-3.6	9.9
TS		-771.2249	0.2923	0.3574	-406.45	-772.5896	15.5	28.3	15.1	13.2
PS	ester	-542.5872	0.2387	0.2940	None	-543.5426	-16.0	-14.6	-15.3	0.8
	AcOH	-228.6777	0.0355	0.0651	None	-229.0973				

Table S11. Validation of level of theory by comparison of single point energies with CPCM(CHCl3)-DLPNO-CCSD(T)/def2-TZVPP.

	CPCM(Ch2Cl2)-DLPNO-CCSD(T)/def2-TZVPP		D3BJ-CPCM($CHCl_3$)-PBE0/def2-SVP		CPCM($CHCl_3$)-M06-2X/def2-TZVP		CPCM($CHCl_3$)-ωB97X-D3/def2-TZVPP	
	E [Eh]	ΔE [kcal/mol]	E [Eh]	ΔE [kcal/mol]	E [Eh]	ΔE [kcal/mol]	E [Eh]	ΔE [kcal/mol]
Alcohol	-390.24		-390.193		-390.875		-390.972	
Ac_2O	-381.17		-381.044		-381.739		-381.791	
TS	-771.38	16.95	-771.225	7.67	-772.59	15.52	-772.739	14.99
Ester	-542.67	-15.84	-542.587	-17.45	-543.543	-16.03	-543.66	-16.41
AcOH	-228.77		-228.678		-229.097		-229.13	
			RMSE	6.76	RMSE	1.02	RMSE	1.45

Catalyzed esterification reaction

We investigated 3 possible mechanisms of the acyl transfer reaction to alcohol in cage **$1Ac_1$** involving (i) transition states analogous to the background reaction; (ii) formation of a cage carboxylate to assist alcohol deprotonation, with the proton located at a pyridine or other basic site, and (iii) concerted proton transfers involving the cage acid group as base/acid.

Investigation of mechanism (i)

Mechanism (i) assumes that the esterification reaction in the cage mirrors the analogous transition states observed in the background reaction (**Figure S36; Table S10**), with stabilization potentially arising from dielectric changes relative to the bulk solvent, or through stabilization by a hydrogen bond (involving the second carboxylic acid group). Two possible transition states were found: one with the hydrogen atom (from the carboxylic acid group) oriented toward the oxygen of the acyl group and another with it positioned away from it (**Figure S37**). In both cases, the activation barrier resembled that of the uncatalyzed reaction ($\Delta G^{\ddagger}_{uncat}$=28.3 kcal/mol): the $\Delta G^{\ddagger}$ values are 25.6 and 25.1 kcal/mol with and without the hydrogen bond, respectively (**Table S12**).

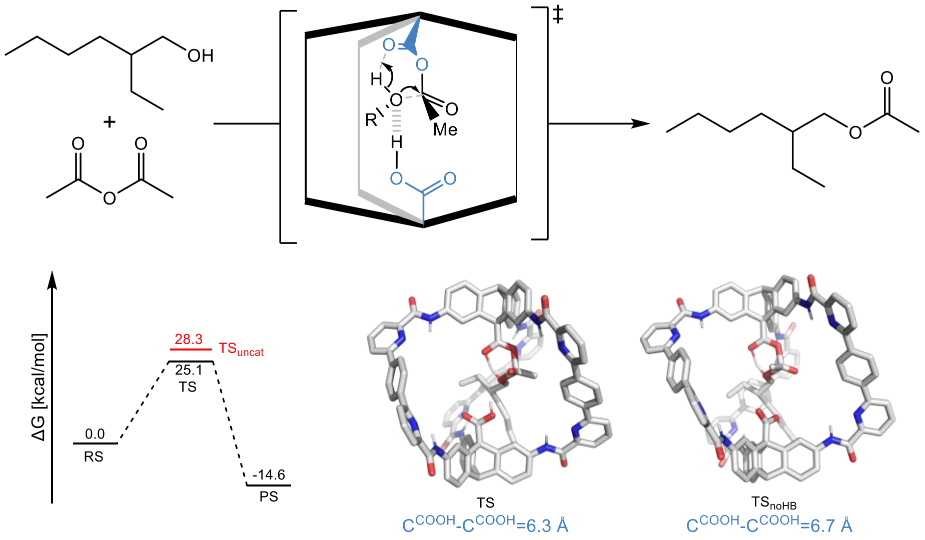

Figure S37. Proposed mechanism (i) with a TS analogous to the background, with and without a minor assisting role from the second carboxylic acid group. Energy profiles at CPCM($CHCl_3$)-M06-2X/def2-TZVP//D3BJ-CPCM($CHCl_3$)-PBE0/def2-SVP level of theory.

Investigation of mechanism (ii)

The increased catalytic activity of the unreactive cage **3** upon adding exogeneous pyridine suggests a potential basic role for the pyridine units in cage **1** (similar to acid-base catalysis observed in enzymes). In mechanism (ii), an initial proton transfer from the carboxylic acid group to a pyridine occurs, forming a zwitterionic cage. Subsequently, the oxygen atom of the alcohol group performs a nucleophilic attack on the acyl group while simultaneously transferring its proton to the carboxylic anion. However, DFT calculations indicate that the energy required to form the zwitterionic state is significant (25.5 kcal/mol), followed by an additional 14.8 kcal/mol to reach the transition state (**Figure S38**; **Table S12**). Additional attempts to position the proton at the anhydride, or to protonate a pyridine group after initial carbonyl rotation to access **C12**, did not result in competitive transition states. Therefore, we are unable to report an energetically tractable pathway for mechanism (ii).

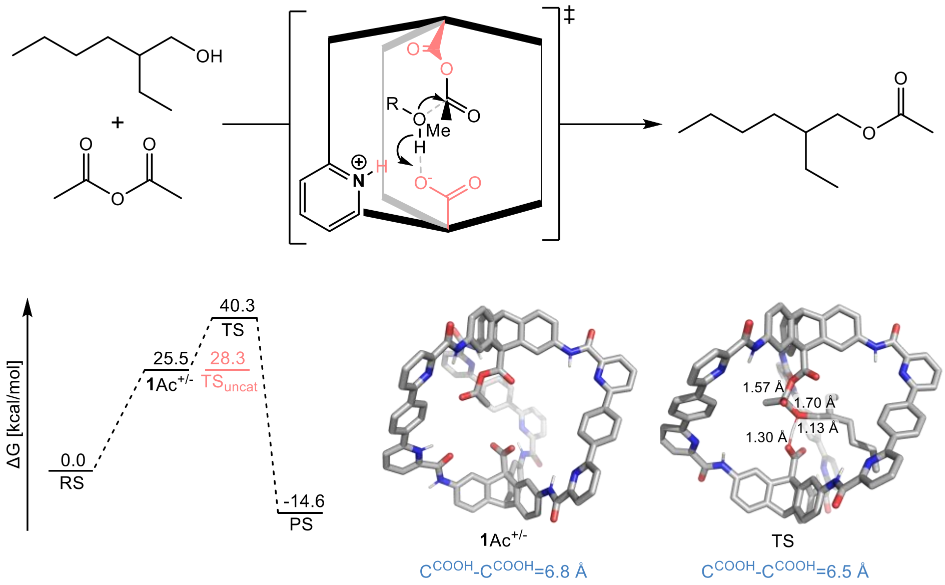

Figure S38. Proposed mechanism (ii) involving zwitterion formation and carboxylate assisted alcohol attack. Energy profiles at CPCM($CHCl_3$)-M06-2X/def2-TZVP//D3BJ-CPCM($CHCl_3$)-PBE0/def2-SVP level of theory.

Investigation of mechanism (iii)

In mechanism (iii), the cage promotes protic activation of the anhydride carbonyl group via the carboxylic acid, and basic activation of the alcohol by proton transfer to the incipient carboxylate group, resulting in a cyclic transition state. As a result, the alcohol attacks the acyl group to form a stabilized (protonated) tetrahedral intermediate, which can rearrange into the final product by ejection of cage **1**. The single-point calculations show that the nucleophilic attack of the alcohol and proton transfer is the rate-limiting step ($\Delta G^{\ddagger}$ = 16.8 kcal/mol; **Figure S39**; **Table S12**), which is typical for esterification reactions (e.g. see the background reaction mechanism, **Figure S36**). In agreement with the experimental result from the Eyring analysis, there is a much lower enthalpic contribution to the transition state, $\Delta H^{\ddagger}_{calculated\_cat}$ = 3.8 kcal/mol ($\Delta H^{\ddagger}_{experimental\_cat}$ = 6.4 kcal/mol) in contrast to the background reaction, $\Delta H^{\ddagger}_{calculated\_bkg}$ = 15.1 kcal/mol ($\Delta H^{\ddagger}_{experimental\_bkg}$ = 13.6 kcal/mol), indicating that the cage stabilizes the transition state.

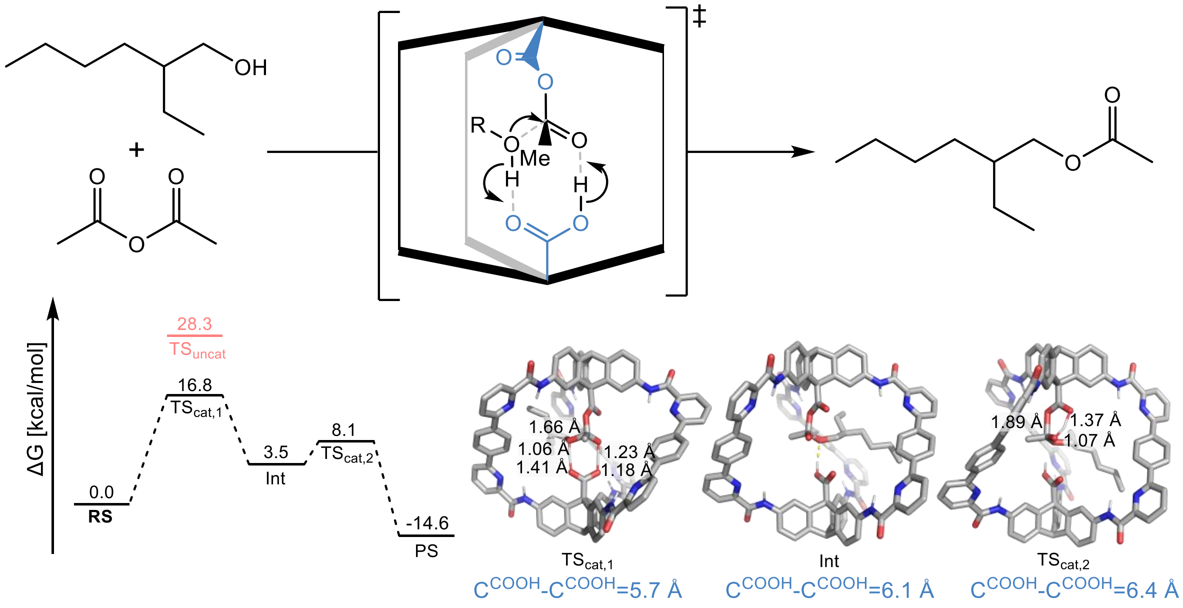

Figure S39. Proposed mechanism (iii) involving both carboxylic groups and energy profiles CPCM($CHCl_3$)-M06-2X/def2-TZVP//D3BJ-CPCM($CHCl_3$)-PBE0/def2-SVP level of theory.

Table S12. Single point energies and thermal contributions for the three proposed cage-catalyzed mechanisms.

	D3BJ-CPCM(CHCl3)-PBE0/def2-SVP//D3BJ-CPCM(CHCl3)-PBE0/def2-SVP				CPCM(CHCl3)-M06-2X/def2-TZVP//D3BJ-CPCM(CHCl3)-PBE0/def2-SVP				
	E [Eh]	G-E [Eh]	H-E [Eh]	im. Freq [cm^{-1}]	E [Eh]	ΔE [kcal/mol]	ΔG [kcal/mol]	ΔH [kcal/mol]	−TΔS [kcal/mol]
Reactant State									
Effective Boltzmann reactant state						0	0	0	0
Transition state: mechanism (i)									
TS_1	-5636.2287	1.5264	1.7485	-403	-5646.1816	14.5	25.6	13.8	11.7
TS_1 (no H-B)	-5636.2252	1.5262	1.7494	-297.35	-5646.1809	14.9	25.1	14.8	10.3
Transition state: mechanism (ii)									
1Ac$_1$ZI_C12	-5245.9871	1.2839	1.4853	None	-5255.2813	30.6	25.5	30.3	-4.8
1Ac$_1$ZI_C13	-5245.9842	1.2846	1.4852	None	-5255.2791	31.9	27.3	31.6	-4.3
$(6\subset 1Ac_1ZI)_A$	-5636.2154	1.5267	1.7525	None	-5646.171351	20.9	32.3	32.1	0.2
$(6\subset 1Ac_1ZI)_B$	-5636.2194	1.5254	1.7522	-8.41	-5646.176074	18.0	28.6	29.0	-0.4
TS_3_zwitterion_C12	-5636.2116	1.5254	1.7474	-338.75	-5646.1572	29.8	40.3	37.8	2.5
Transition state: mechanism (iii)									
TS_2_1	-5636.2457	1.5252	1.7454	-793.48	-5646.1945	6.4	16.8	3.8	13.0
Int	-5636.2694	1.5309	1.7525	None	-5646.2213	-10.4	3.5	-8.6	12.0
TS_2_2	-5636.2603	1.5276	1.7488	-407.85	-5646.2106	-3.7	8.1	-4.2	12.3
Product State									
**1Ac$_1$**	-5246.0311	1.2840	1.4847	None	-5255.3221				
**1**	-5093.6539	1.2502	1.4448	None	-5102.6775				
ester	-542.5872	0.2500	0.3050	None	-543.5420	-9.3	-14.6	-9.1	-5.5

Distortion-Interaction analysis

To trace the origin of the catalytic activity, we utilized the distortion-interaction (D/I) framework to analyze the results.[33] We performed the analysis for the transition state structures with (**6⊂1Ac$_1$**)$_A$ as a reference, which has the lowest electronic energy. The results of D/I analysis are shown in **Figure S40** (**Table S13**). The D/I analysis shows that the reaction with the cage follows a less strained mechanism in reference to the background reaction as the distortion is lowered by 7.1 kcal/mol. Lower distortion can be attributed to proton transfer relayed by carboxylic acid, which results in the 8-membered ring TS compared to the 6-membered ring TS for background reaction.

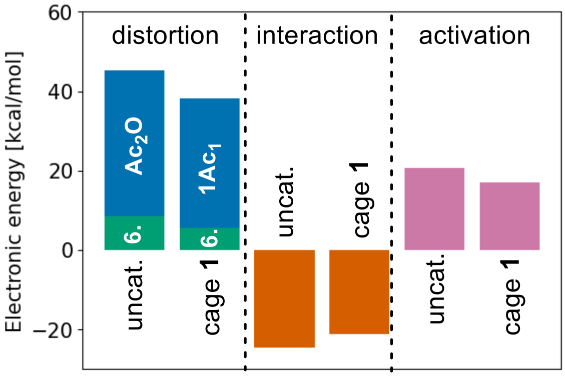

Figure S40. Transition states analyzed using the Distortion-Interaction (D/I) framework: from reactant complexes to the transition state in the uncatalyzed reaction and in the cage **1** promoted reaction.

Table S13. Single point energies for the Distortion-Interaction (D/I) analysis for the background reaction and reactions with cage **1** and cage **3**. Electronic activation barrier calculated relative to the reactant complex.

	E(RC) [Eh]	E(TS) [Eh]	$\Delta E_{distortion}$ [kcal/mol]	$\Delta E_{interaction}$ [kcal/mol]	$\Delta E_{activation}$ [kcal/mol]
background					
Ac_2O	-381.7387	-381.6800	36.8	-24.8	20.6
Alcohol	-390.8747	-390.8612	8.5		
cage **1**					
**1Ac$_1$**	-5255.3182	-5255.2660	32.7	-21.3	16.9
Alcohol	-390.8746	-390.8659	5.5		

Comparison with cage 3. To explore the role of the pyridine groups within cage **1**, we computed the activation barrier for its benzene analogue, cage **3**. The resulting activation barrier ($\Delta G^{\ddagger}$ = 24.9 kcal/mol; **Figure S41, Table 14**) is 9.1 kcal/mol higher than for cage **1**. The C^{COOH}-C^{COOH} distance of the optimized structure of cage **3** (in configuration **C5**) is 9.3 Å, suggesting that the cage requires a more substantial conformational change to reach the TS (C^{COOH}-C^{COOH} distance of 5.8 Å) than cage **1** (C^{COOH}-C^{COOH} distance of 6.6 Å). These findings suggest that a primary role of the pyridine groups within cage **1** is to access favorable conformations, and to increase its flexibility (to reduce strain). There may be an, as yet, unaccounted for role of the free pyridine in activating cage **3** as an acyl transfer catalyst.

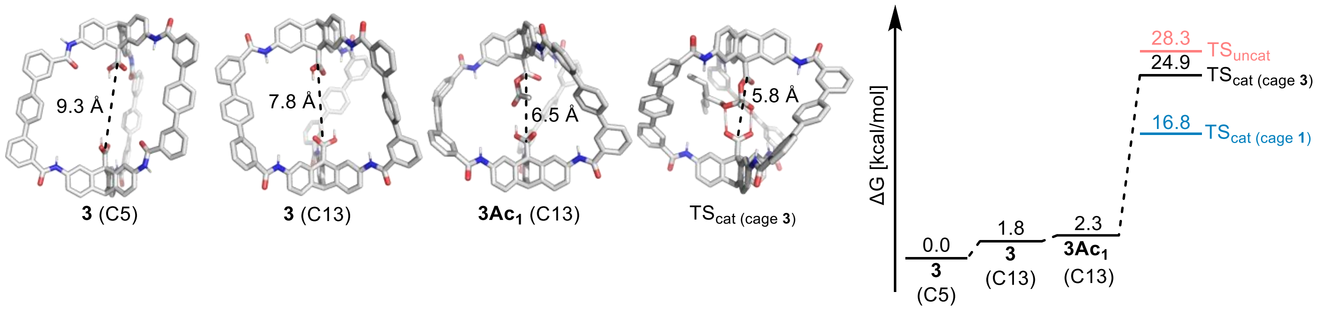

Figure S41. Calculated structures and energies for the esterification reaction catalyzed by cage **3**

Table S14. Single point energies and thermal contributions for mechanism (iii) for cage **3**.

	D3BJ-CPCM(CHCl3)-PBE0/def2-SVP//D3BJ-CPCM(CHCl3)-PBE0/def2-SVP				CPCM(CHCl3)-M06-2X/def2-TZVP//D3BJ-CPCM(CHCl3)-PBE0/def2-SVP				
	E [Eh]	G-E [Eh]	H-E[Eh]	im. Freq [cm^{-1}]	E [Eh]	ΔE [kcal/mol]	ΔG [kcal/mol]	ΔH [kcal/mol]	–TΔS [kcal/mol]
Reactant state									
Ac_2O	-381.0442	0.0699	0.1075	None	-381.7395				
AcOH	-228.6724	0.0381	0.0651	None	-229.0973				
6	-390.1930	0.2167	0.2638	None	-390.8747				
**3 (C5)**	-4997.5481	1.3226	1.5182	None	-5006.3797	0.0	0.0	0.0	0.0
**3 (C13)**	-4997.5393	1.3208	1.5173	None	-5006.3750	2.9	1.8	2.4	-0.6
**3Ac₁**	-5149.9118	1.3555	1.5580	None	-5159.0194	1.6	2.3	-0.1	2.3
(6⊂**1Ac₁**)A	-5540.1492	1.5956	1.8246	None	-5549.9093	-8.0	7.4	-7.9	15.3
(6⊂**1Ac₁**)B	-5540.1498	1.5990	1.8238	None	-5549.9173	-13.0	4.5	-13.4	17.9
(6⊂**1Ac₁**)C	-5540.1419	1.5981	1.8248	None	-5549.9093	-8.0	8.9	-7.8	16.7
Transition state: mechanism (ii)									
TS_2_1		1.5977	1.8188	-793.48	-5549.8835	8.2	24.9	4.7	20.2

Pyridine cage conformation may affect basicity

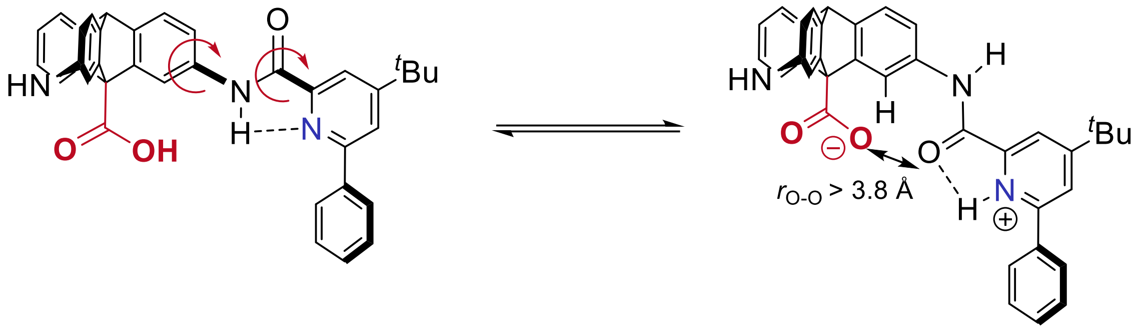

Figure S42. Amide group rotation and pyridine basicity in cage **1**. The “carbonyl-out” conformer (left) shows the pyridine and carbonyl dipoles away from each other. There may be an attractive NH---pyr interaction that lessens the basicity of the pyridyl nitrogen. We do not suspect (see main text) the pyridine atoms act as bases during catalysis. However, it is worth noting that the less stable conformer with the “carbonyl in” (right) has no such H-bond, and should have increased basicity in order to counter the carbonyl/pyridine dipole alignment, and so may (in theory) better promote carboxylate formation than the left-hand conformation. The carboxylate-amide oxygen atoms in this species are about 3.4-4.1 Å apart; the amide carbonyl may act as a mediator to transfer the proton to the pyridyl nitrogen.

Supporting Information References

(1) Kaminski, T.; Gros, P.; Fort, Y. Side-Chain Retention During Lithiation of 4-Picoline and 3,4-Lutidine: Easy Access to Molecular Diversity in Pyridine Series. *European J Org Chem* **2003**, *2003* (19), 3855–3860. https://doi.org/10.1002/EJOC.200300243.

(2) Buonomo, J. A.; Everson, D. A.; Weix, D. J. Substituted 2,2′-Bipyridines by Nickel Catalysis: 4,4′-Di-Tert-Butyl-2,2′-Bipyridine. *Synthesis (Stuttg)* **2013**, *45* (22), 3099–3102. https://doi.org/10.1055/S-0033-1338520.

(3) Yoon, I.; Suh, S. E.; Barros, S. A.; Chenoweth, D. M. Synthesis of 9-Substituted Triptycene Building Blocks for Solid-Phase Diversification and Nucleic Acid Junction Targeting. *Org Lett* **2016**, *18* (5), 1096–1099. https://doi.org/10.1021/acs.orglett.6b00169.

(4) Friedman, Lester.; Logullo, F. M. Benzynes via Aprotic Diazotization of Anthranilic Acids: A Convenient Synthesis of Triptycene and Derivatives. *J Am Chem Soc* **1963**, *85* (10), 1549. https://doi.org/10.1021/ja00893a045.

(5) Granda, J. M.; Grabowski, J.; Jurczak, J. Synthesis, Structure, and Complexation Properties of a C3-Symmetrical Triptycene-Based Anion Receptor: Selectivity for Dihydrogen Phosphate. *Org Lett* **2015**, *17* (23), 5882–5885. https://doi.org/10.1021/acs.orglett.5b03066.

(6) Andrews, K. G.; Christensen, K. E. Access to Amide-linked Organic Cages by in Situ Trapping of Metastable Imine Assemblies: Solution Phase Bisamine Recognition. *Chemistry – A European Journal* **2023**, e202300063. https://doi.org/10.1002/chem.202300063.

(7) Nicolaou, K. C.; Baran, P. S.; Zhong, Y. L.; Choi, H. S.; Yoon, W. H.; He, Y.; Fong, K. C. Total Synthesis of the CP Molecules CP-263,114 and CP-225,917- Part 1: Synthesis of Key Intermediates and Intelligence Gathering. *Angewandte Chemie - International Edition* **1999**, *38* (11), 1669–1675. https://doi.org/10.1002/(SICI)1521-3773(19990601)38:11<1669::AID-ANIE1669>3.0.CO;2-D.

(8) Chevella, D.; Thota, C.; Majumder, S. Zirconium Oxychloride Hydrate: An Efficient and Reusable Catalyst for Retro-Claisen Condensation of Alcohols with 1,3-Diketones. *Org Biomol Chem* **2023**, *21* (18), 3837–3843. https://doi.org/10.1039/D3OB00116D.

(9) Andrews, K. G.; Horton, P. N.; Coles, S. J. Programmable Synthesis of Organic Cages with Reduced Symmetry. *Chem Sci* **2024**, *15* (17), 6536–6543. https://doi.org/10.1039/D4SC00889H.

(10) Dolomanov, O. V.; Bourhis, L. J.; Gildea, R. J.; Howard, J. A. K.; Puschmann, H. OLEX2: A Complete Structure Solution, Refinement and Analysis Program. *J Appl Crystallogr* **2009**, *42* (2). https://doi.org/10.1107/S0021889808042726.

(11) Cho, Y. S.; Lim, H. S. Comparison of Various Estimation Methods for the Parameters of Michaelis-Menten Equation Based on in Vitro Elimination Kinetic Simulation Data. *Transl Clin Pharmacol* **2018**, *26* (1), 39–47. https://doi.org/10.12793/TCP.2018.26.1.39.

(12) Grimme, S.; Bannwarth, C.; Shushkov, P. A Robust and Accurate Tight-Binding Quantum Chemical Method for Structures, Vibrational Frequencies, and Noncovalent Interactions of Large Molecular Systems Parametrized for All Spd-Block Elements (Z = 1-86). *J Chem Theory Comput* **2017**, *13* (5). https://doi.org/10.1021/acs.jctc.7b00118.

(13) Bannwarth, C.; Caldeweyher, E.; Ehlert, S.; Hansen, A.; Pracht, P.; Seibert, J.; Spicher, S.; Grimme, S. Extended Tight-Binding Quantum Chemistry Methods. *Wiley Interdiscip Rev Comput Mol Sci* **2021**, *11* (2), e1493. https://doi.org/10.1002/wcms.1493.

(14) Wang, J.; Wang, W.; Kollman, P. A.; Case, D. A. Automatic Atom Type and Bond Type Perception in Molecular Mechanical Calculations. *J Mol Graph Model* **2006**, *25* (2), 247–260. https://doi.org/10.1016/j.jmgm.2005.12.005.

(15) M. J. Frisch, G. W. Trucks, H. B. Schlegel, G. E. Scuseria, M. A. Robb, J. R. Cheeseman, G. Scalmani, V. Barone, G. A. Petersson, H. Nakatsuji, X. Li, M. Caricato, A. V. Marenich, J. Bloino, B. G. Janesko, R. Gomperts, B. Mennucci, H. P. Hratchian, J. V., and D. J. F. Gaussian 16. Gaussian, Inc.: Wallingford CT 2016.

(16) van der Spoel, D.; van Maaren, P. J.; Caleman, C. GROMACS Molecule & Liquid Database. *Bioinformatics* **2012**, *28* (5). https://doi.org/10.1093/bioinformatics/bts020.

(17) Abraham, M. J.; Murtola, T.; Schulz, R.; Pall, S.; Smith, J. C.; Hess, B.; Lindah, E. Gromacs: High Performance Molecular Simulations through Multi-Level Parallelism from Laptops to Supercomputers. *SoftwareX* **2015**, *1–2*, 19–25. https://doi.org/10.1016/j.softx.2015.06.001.

(18) Van Der Spoel, D.; Lindahl, E.; Hess, B.; Groenhof, G.; Mark, A. E.; Berendsen, H. J. C. GROMACS: Fast, Flexible, and Free. *J Comput Chem* **2005**, *26* (16), 1701–1718. https://doi.org/10.1002/jcc.20291.

(19) Bussi, G.; Donadio, D.; Parrinello, M. Canonical Sampling through Velocity Rescaling. *Journal of Chemical Physics* **2007**, *126* (1). https://doi.org/10.1063/1.2408420.

(20) Bernetti, M.; Bussi, G. Pressure Control Using Stochastic Cell Rescaling. *Journal of Chemical Physics* **2020**, *153* (11). https://doi.org/10.1063/5.0020514.

(21) Neese, F.; Wennmohs, F.; Becker, U.; Riplinger, C. The ORCA Quantum Chemistry Program Package. *Journal of Chemical Physics* **2020**, *152* (22). https://doi.org/10.1063/5.0004608.

(22) Neese, F. Software Update: The ORCA Program System—Version 5.0. *Wiley Interdisciplinary Reviews: Computational Molecular Science*. 2022. https://doi.org/10.1002/wcms.1606.

(23) Adamo, C.; Barone, V. Toward Reliable Density Functional Methods without Adjustable Parameters: The PBE0 Model. *Journal of Chemical Physics* **1999**, *110* (13). https://doi.org/10.1063/1.478522.

(24) Weigend, F.; Ahlrichs, R. Balanced Basis Sets of Split Valence, Triple Zeta Valence and Quadruple Zeta Valence Quality for H to Rn: Design and Assessment of Accuracy. *Physical Chemistry Chemical Physics* **2005**, *7* (18). https://doi.org/10.1039/b508541a.

(25) Grimme, S.; Antony, J.; Ehrlich, S.; Krieg, H. A Consistent and Accurate Ab Initio Parametrization of Density Functional Dispersion Correction (DFT-D) for the 94 Elements H-Pu. *Journal of Chemical Physics* **2010**, *132* (15). https://doi.org/10.1063/1.3382344.

(26) Grimme, S.; Ehrlich, S.; Goerigk, L. Effect of the Damping Function in Dispersion Corrected Density Functional Theory. *J Comput Chem* **2011**, *32* (7). https://doi.org/10.1002/jcc.21759.

(27) Barone, V.; Cossi, M. Quantum Calculation of Molecular Energies and Energy Gradients in Solution by a Conductor Solvent Model. *Journal of Physical Chemistry A* **1998**, *102* (11). https://doi.org/10.1021/jp9716997.

(28) Grimme, S. Supramolecular Binding Thermodynamics by Dispersion-Corrected Density Functional Theory. *Chemistry - A European Journal* **2012**, *18* (32). https://doi.org/10.1002/chem.201200497.

(29) Zhao, Y.; Truhlar, D. G. The M06 Suite of Density Functionals for Main Group Thermochemistry, Thermochemical Kinetics, Noncovalent Interactions, Excited States, and Transition Elements: Two New Functionals and Systematic Testing of Four M06 Functionals and 12 Other Functionals (Theoretical

Chemistry Accounts DOI 10.1007/S00214-007-0310-x). *Theoretical Chemistry Accounts*. 2008. https://doi.org/10.1007/s00214-007-0401-8.

(30) Young, T. A.; Silcock, J. J.; Sterling, A. J.; Duarte, F. AutodE: Automated Calculation of Reaction Energy Profiles— Application to Organic and Organometallic Reactions. *Angewandte Chemie International Edition* **2021**, *60* (8), 4266–4274. https://doi.org/10.1002/ANIE.202011941.

(31) Zimmerman, P. Reliable Transition State Searches Integrated with the Growing String Method. *J Chem Theory Comput* **2013**, *9* (7). https://doi.org/10.1021/ct400319w.

(32) Zimmerman, P. M. Single-Ended Transition State Finding with the Growing String Method. *J Comput Chem* **2015**, *36* (9). https://doi.org/10.1002/jcc.23833.

(33) Bickelhaupt, F. M.; Houk, K. N. Analyzing Reaction Rates with the Distortion/Interaction-Activation Strain Model. *Angewandte Chemie - International Edition* **2017**, *56* (34), 10070–10086. https://doi.org/10.1002/anie.201701486.

Spectra for:

Enzyme-like acyl transfer catalysis in a bifunctional organic cage

Keith G. Andrews,* Tomasz K. Piskorz, Peter N. Horton, Simon J. Coles

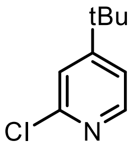

s1

^{1}H NMR (400 MHz, $CDCl_3$)

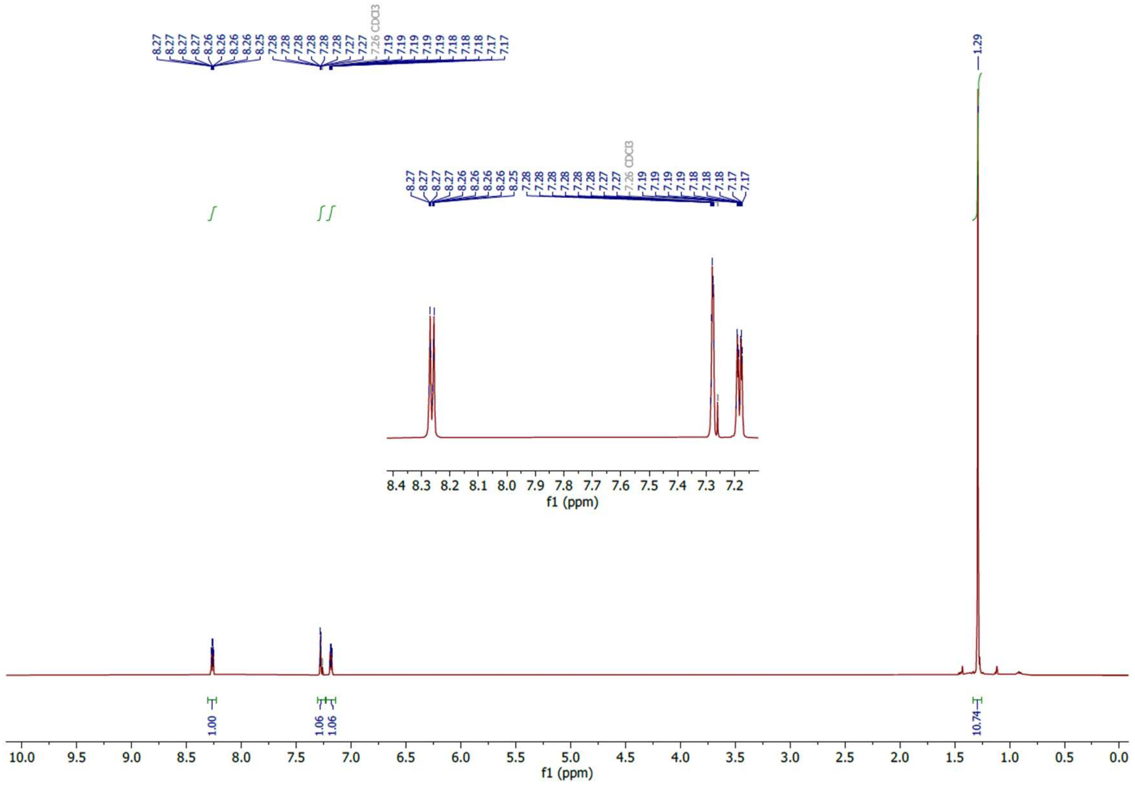

^{13}C NMR (101 MHz, $CDCl_3$)

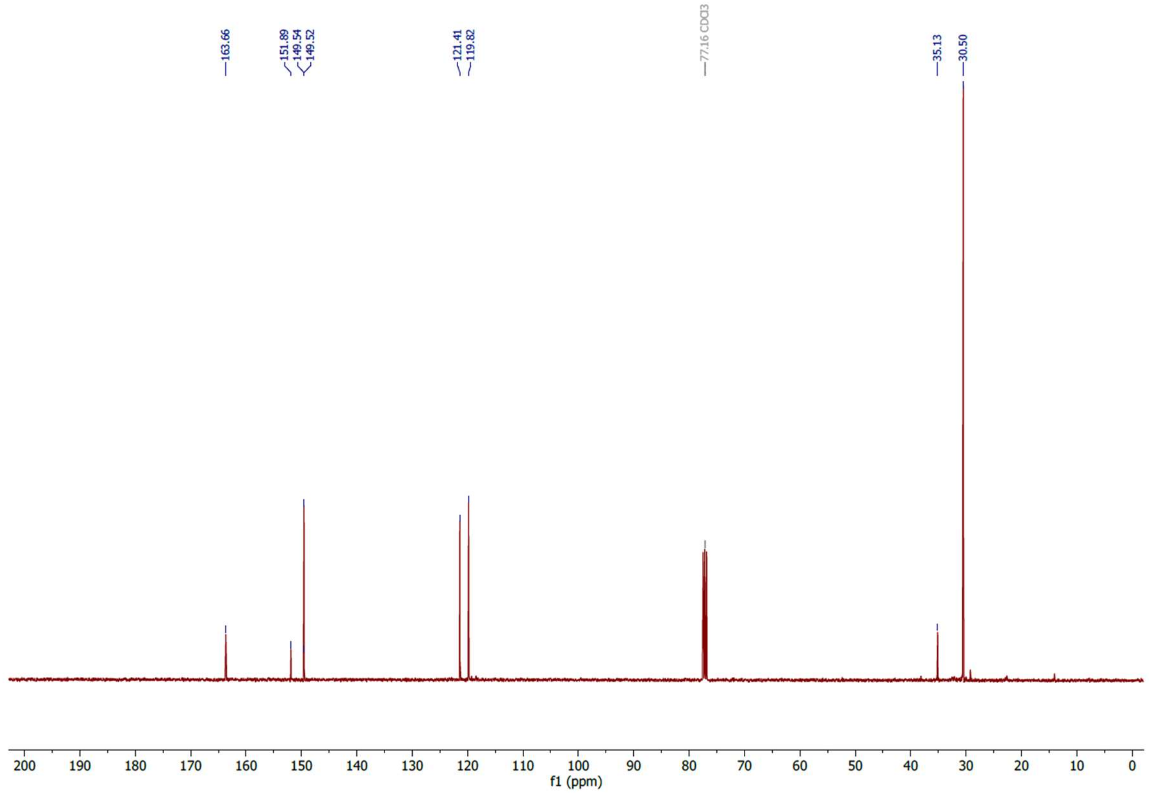

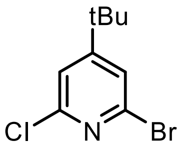

s2

^{1}H NMR (400 MHz, $CDCl_3$)

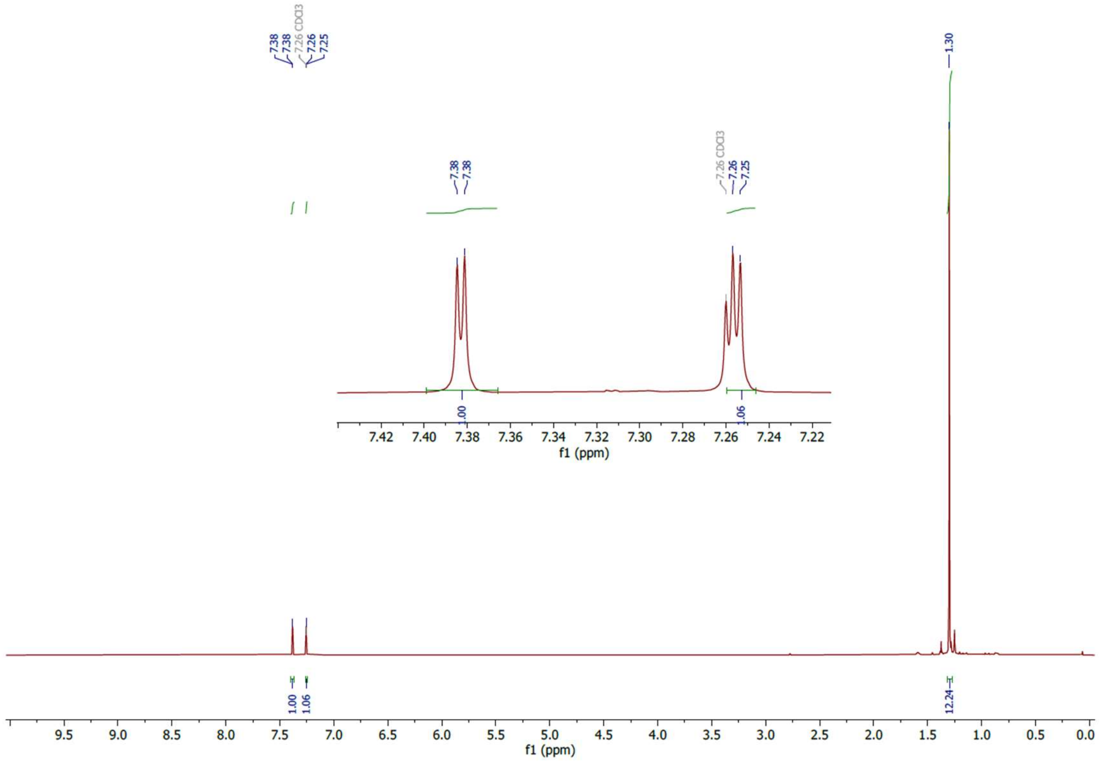

^{13}C NMR (101 MHz, $CDCl_3$)

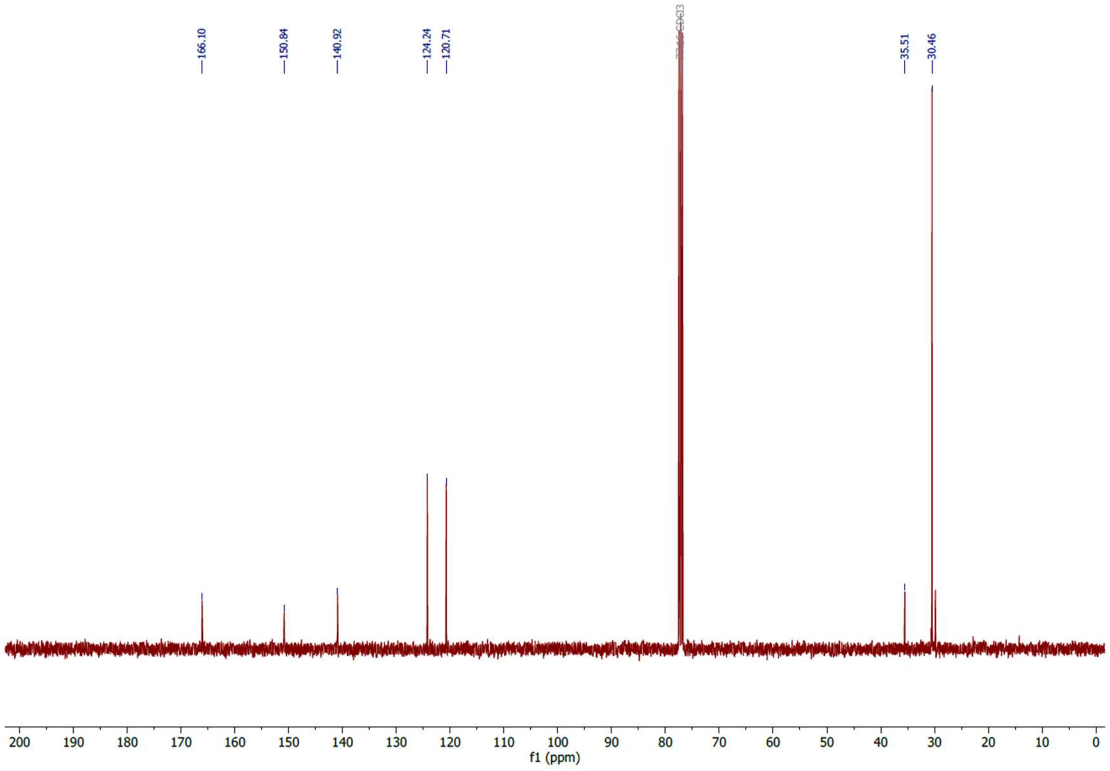

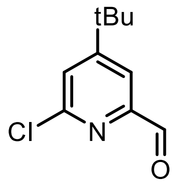

s3

^{1}H NMR (400 MHz, $CDCl_3$)

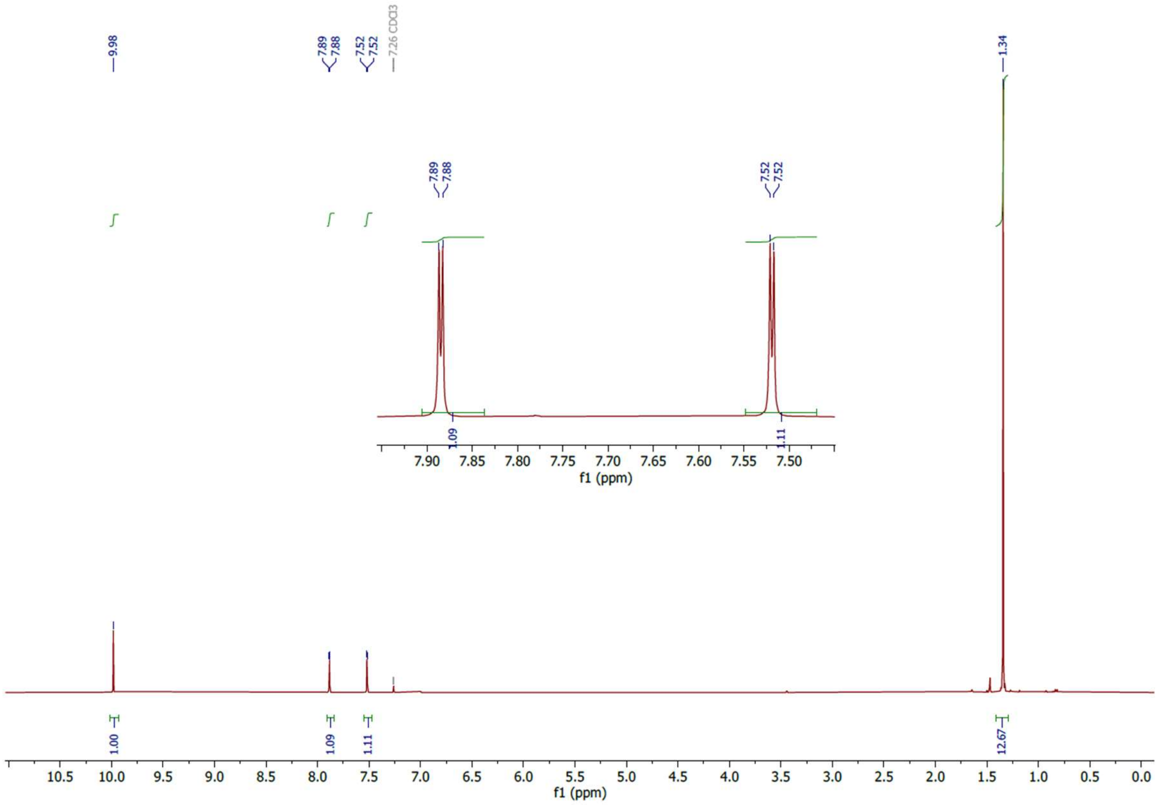

^{13}C NMR (101 MHz, $CDCl_3$)

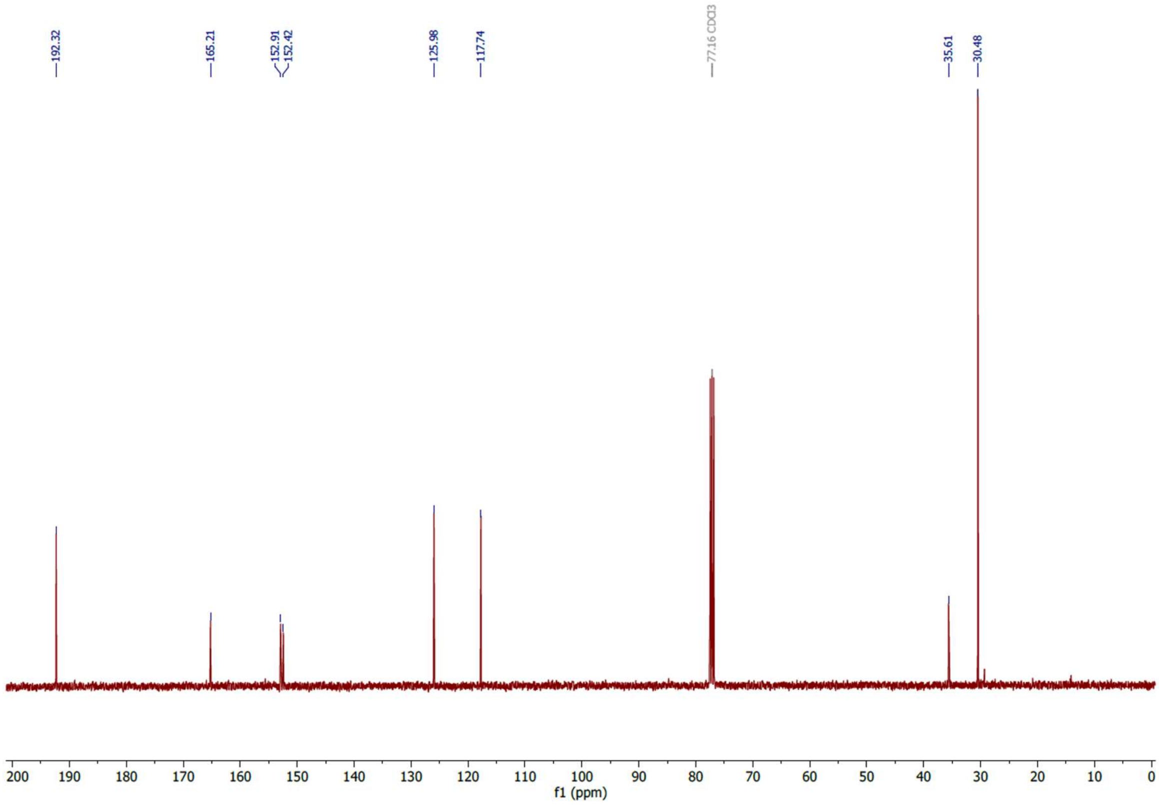

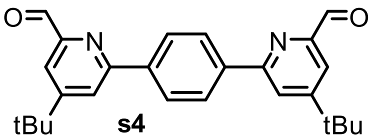

s4

^{1}H NMR (400 MHz, $CDCl_3$)

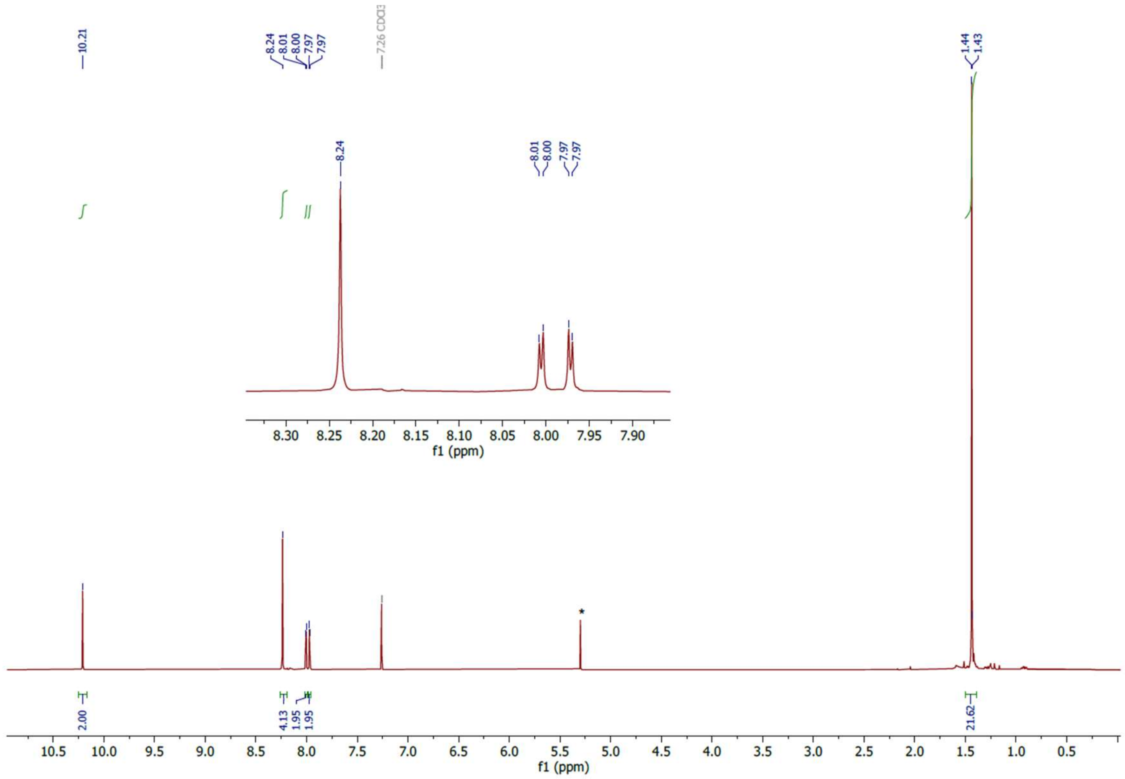

^{13}C NMR (101 MHz, $CDCl_3$)

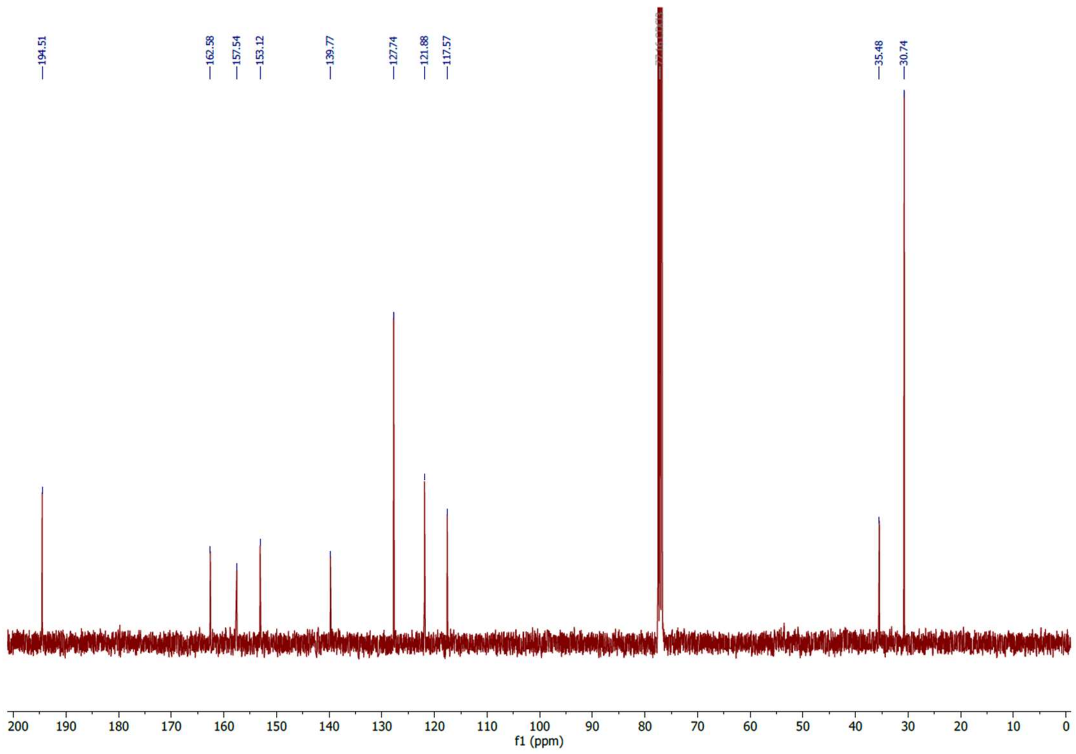

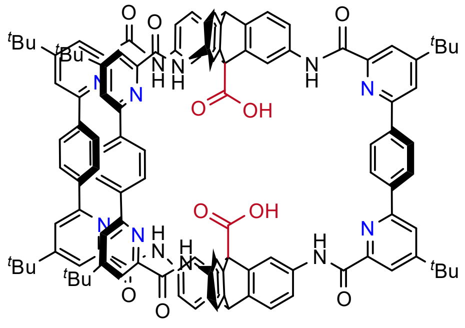

1

^{1}H NMR (400 MHz, $CDCl_3$)

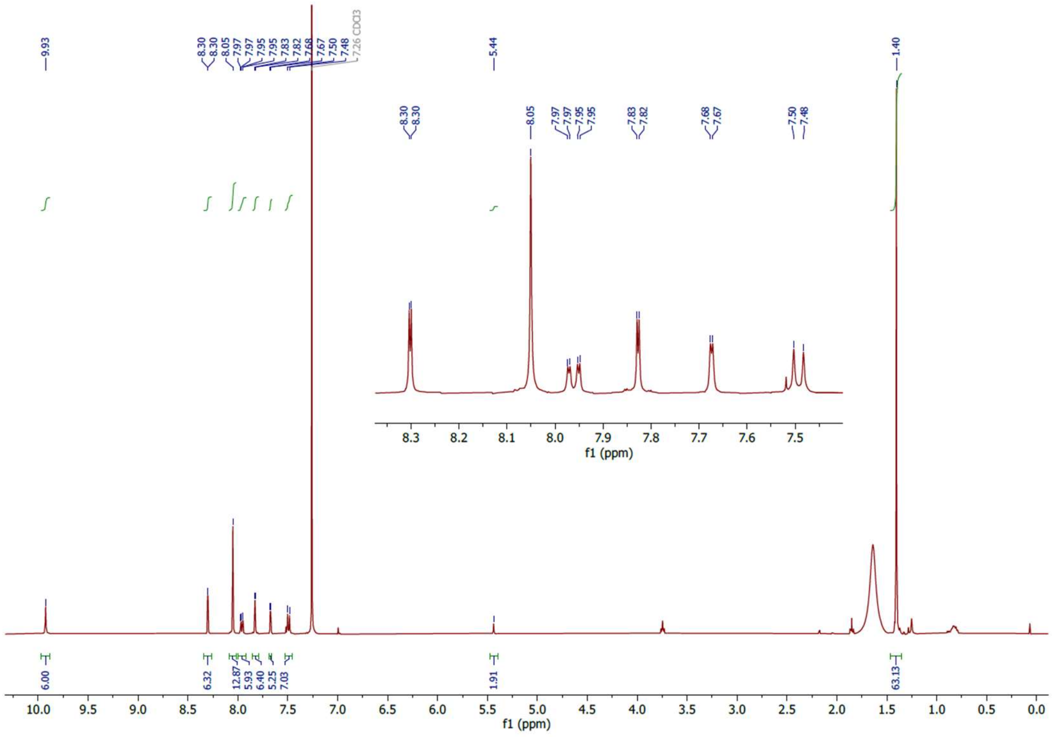

^{13}C NMR (101 MHz, $CDCl_3$)

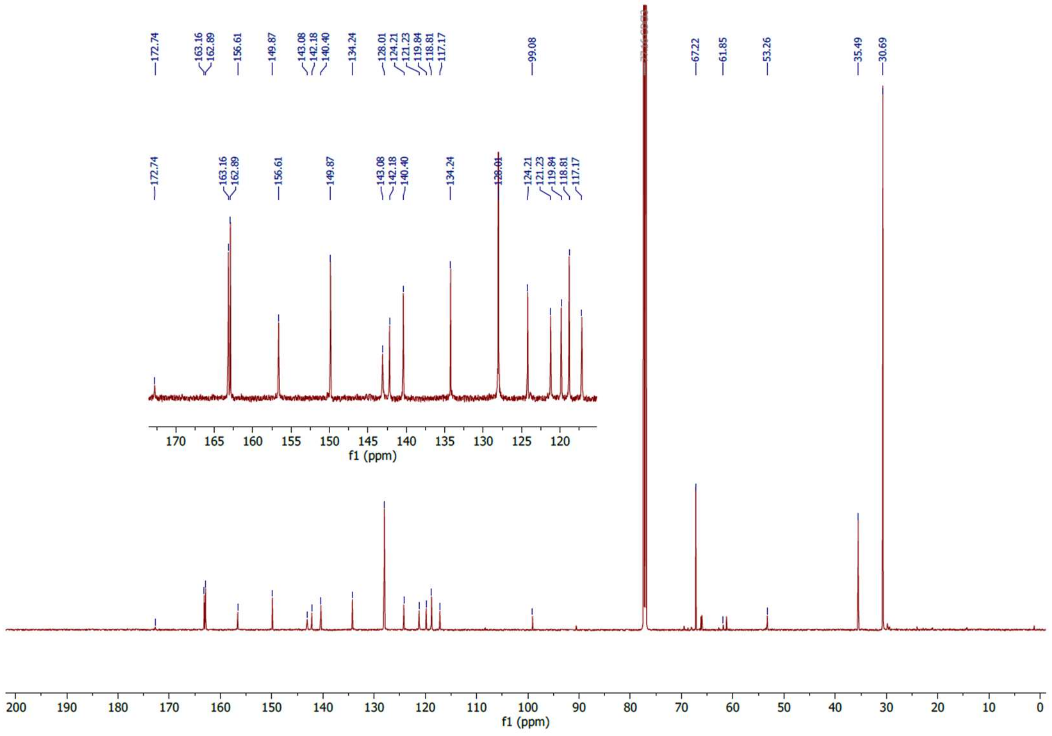

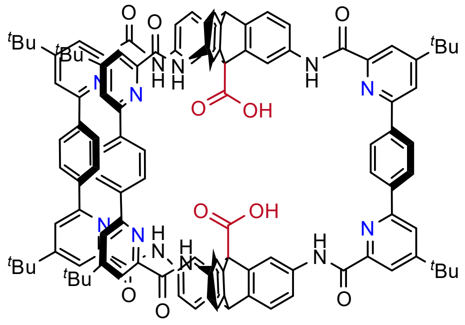

1

MS *m/z* (MALDI-ToF-RP) (DCTB matrix); M, M+Na

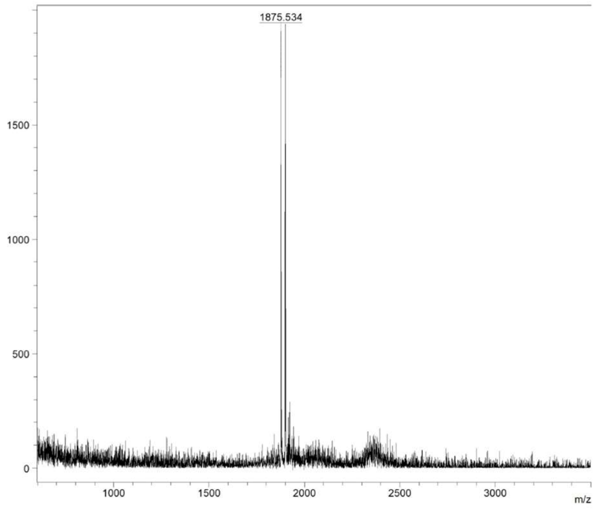

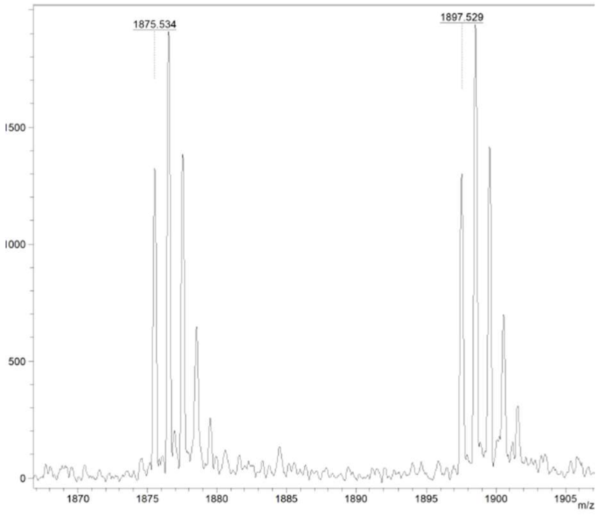

Calculated isotope pattern (top); measured (bottom)

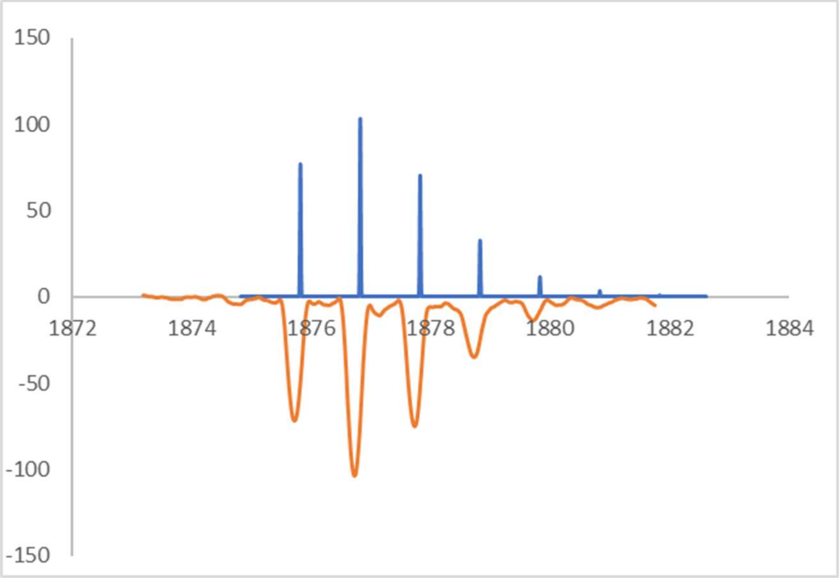

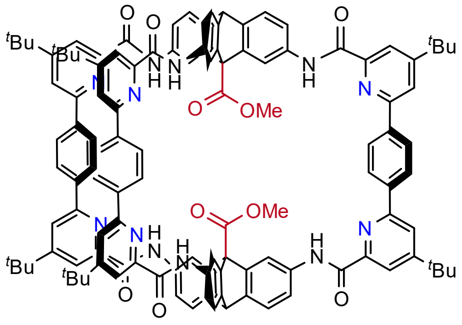

2

[1]**H NMR** (400 MHz, $CDCl_3$)

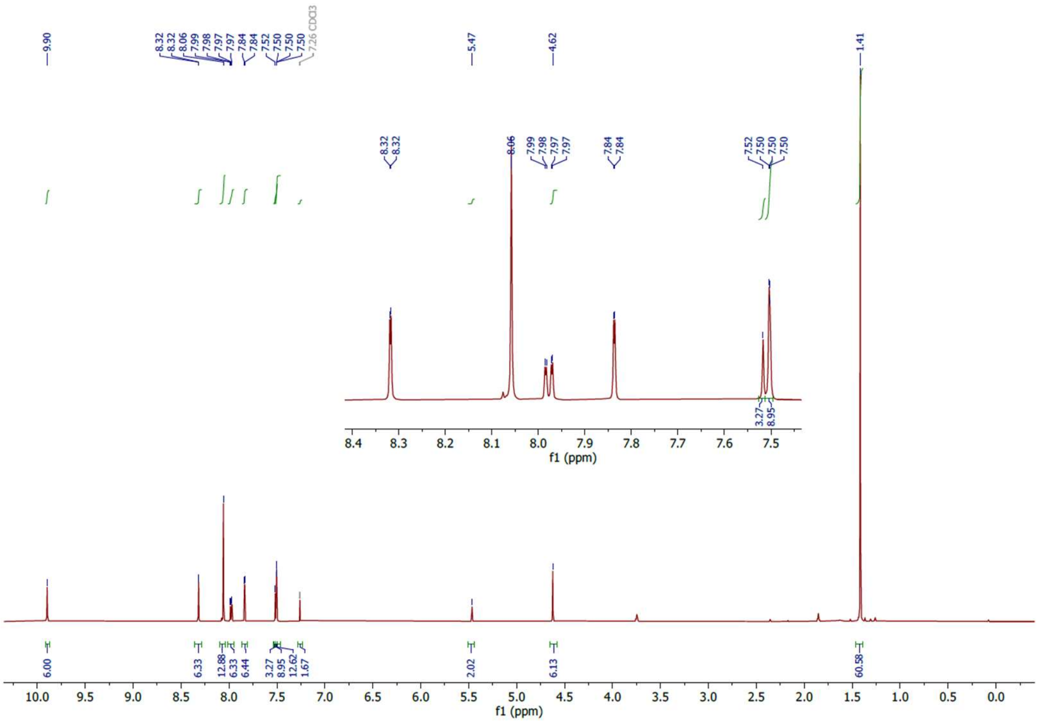

[13]**C NMR** (101 MHz, $CDCl_3$)

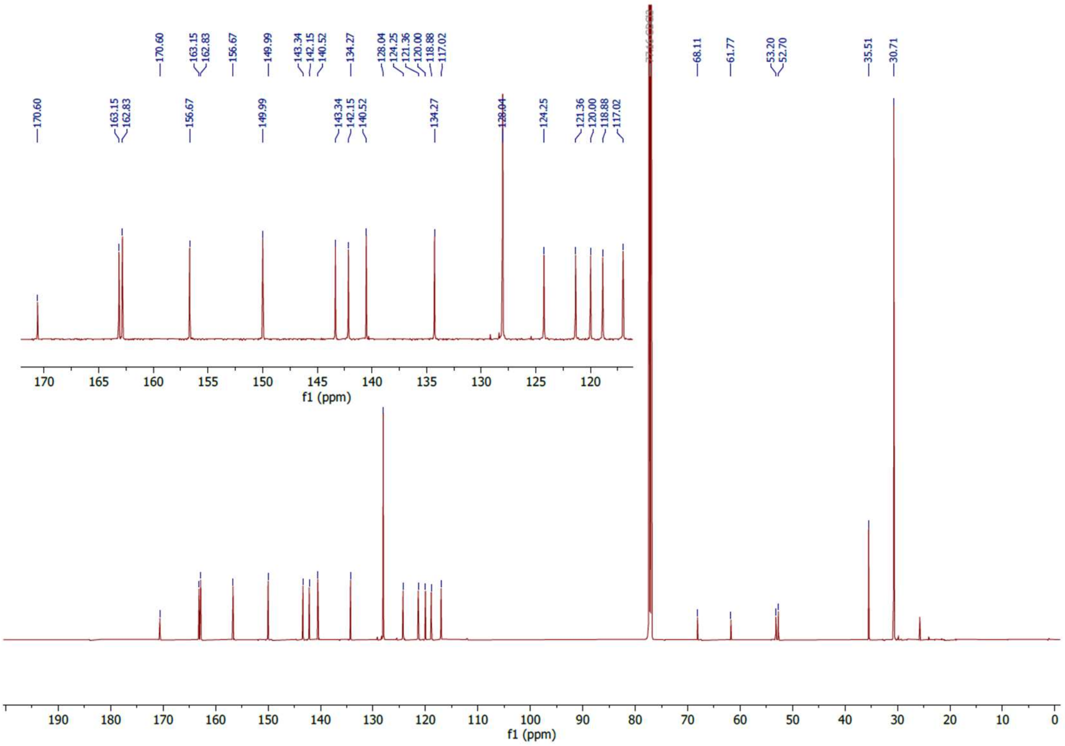

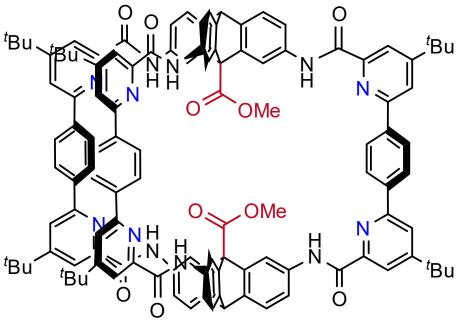

2

MS *m/z* (MALDI-ToF-RP) (DCTB matrix)

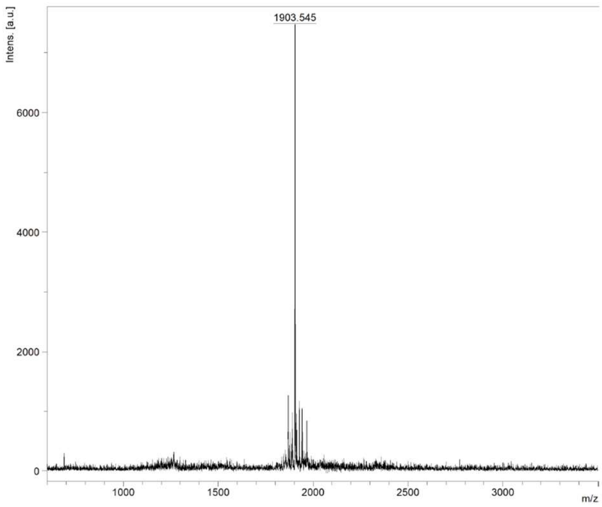

M+H

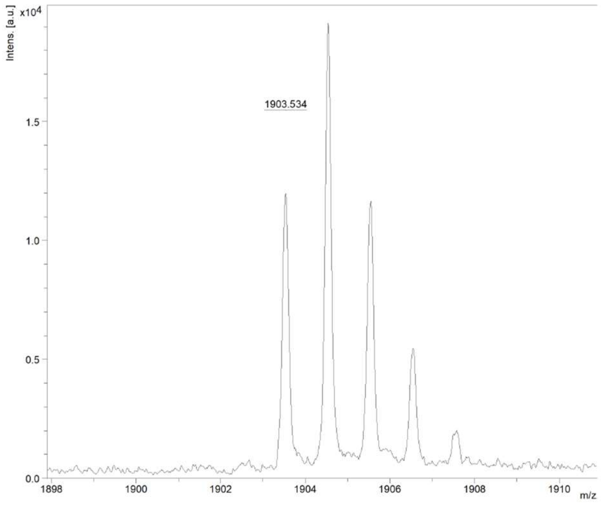

Calculated isotope pattern (top); measured (bottom)

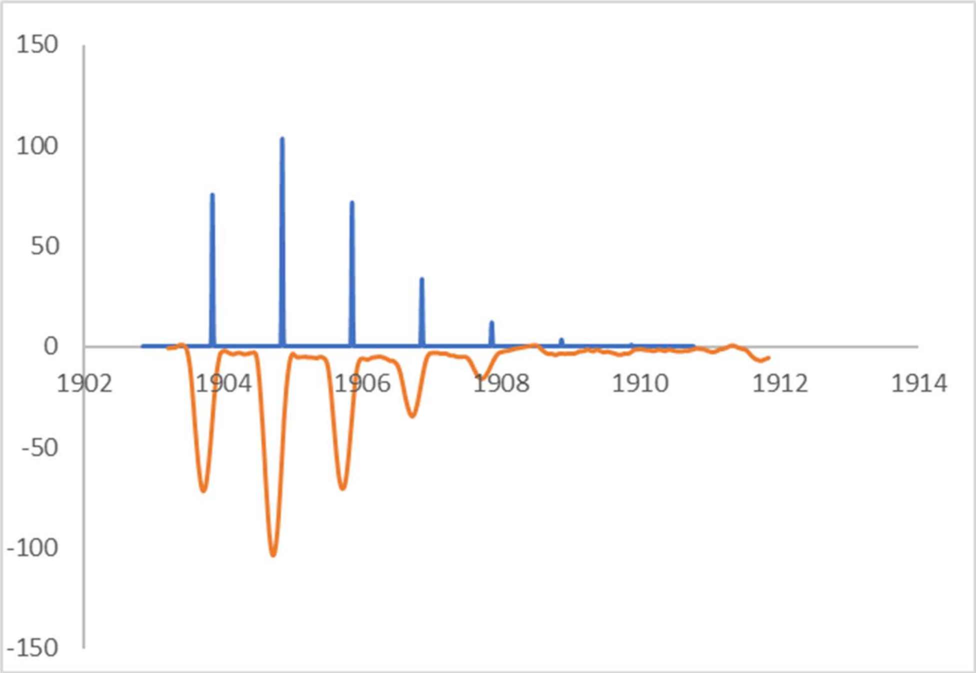

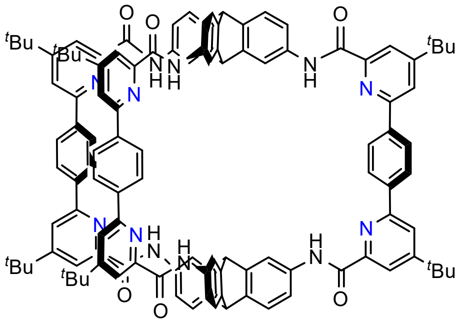

4

^{1}H NMR (400 MHz, $CDCl_3$)

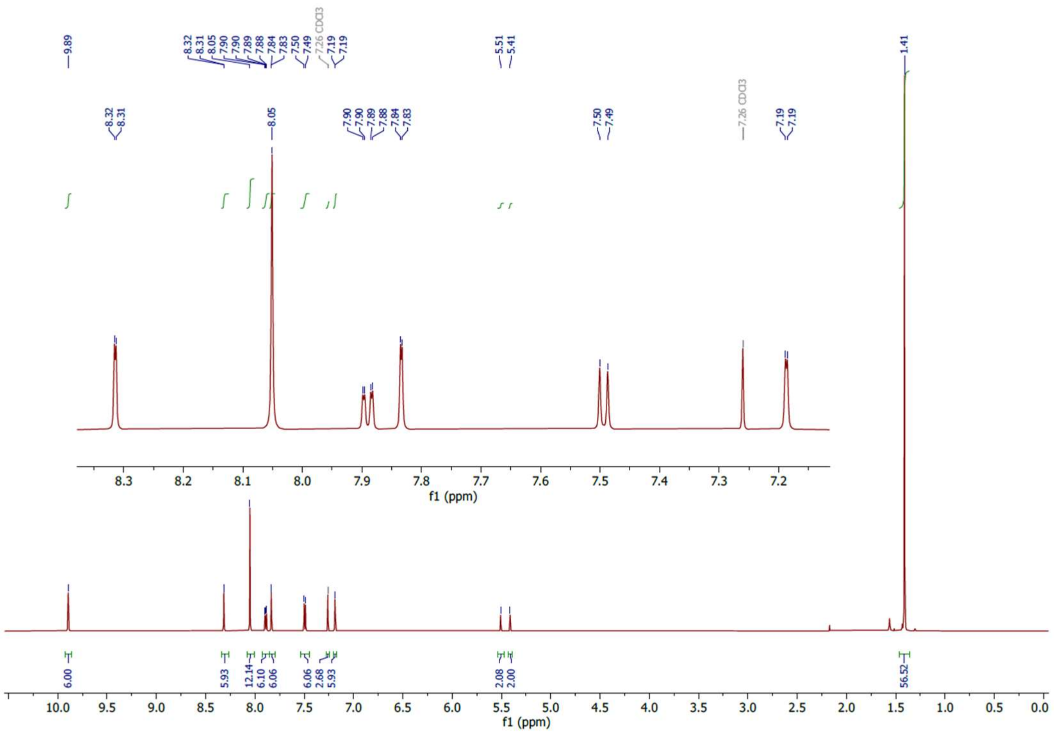

^{13}C NMR (101 MHz, $CDCl_3$)

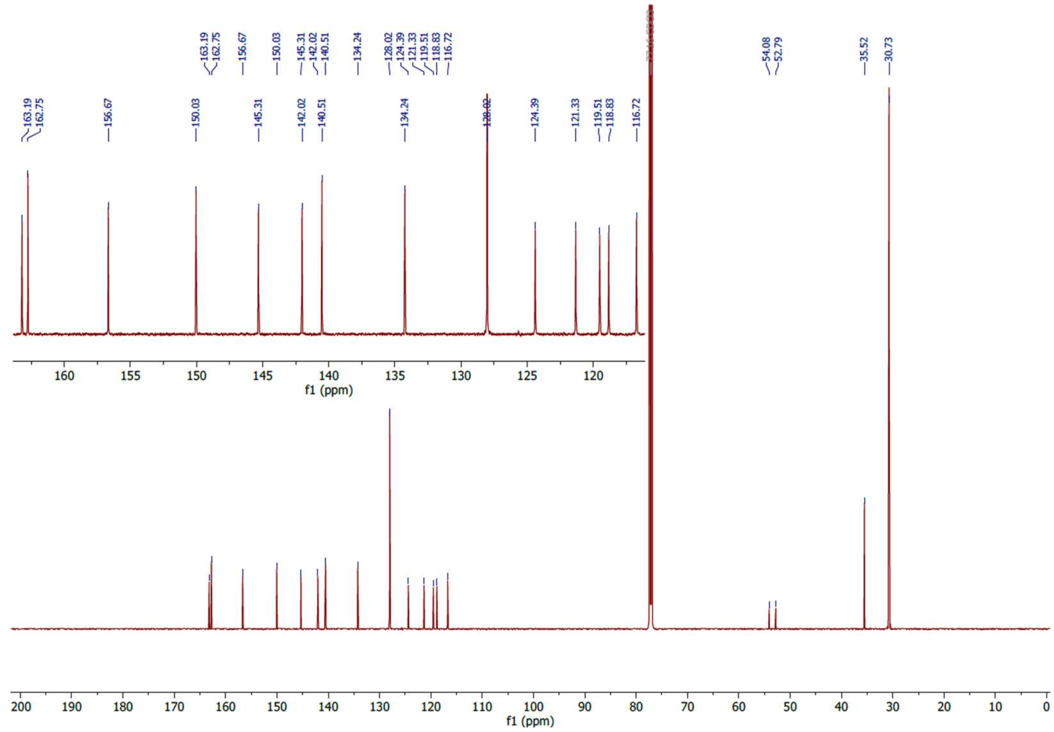

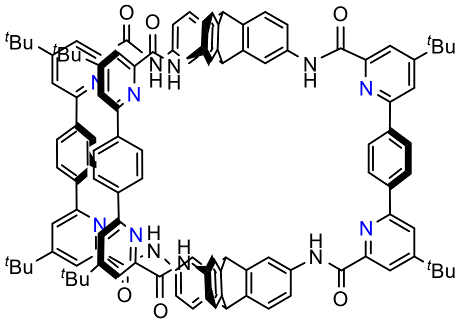

4

MS *m/z* (MALDI-ToF-RP) (DCTB matrix)

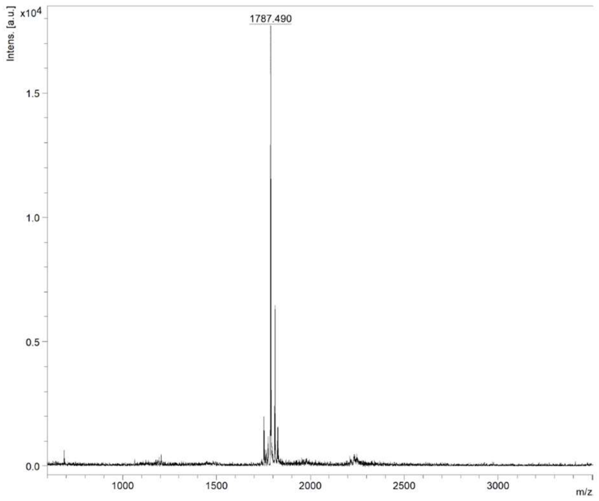

M+H

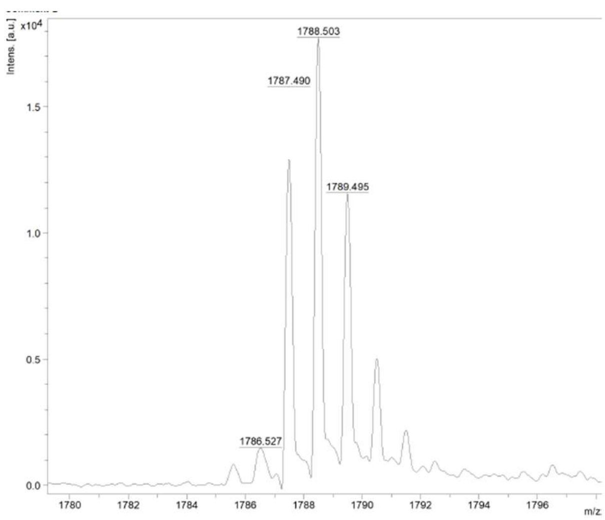

Calculated isotope pattern (top); measured (bottom)

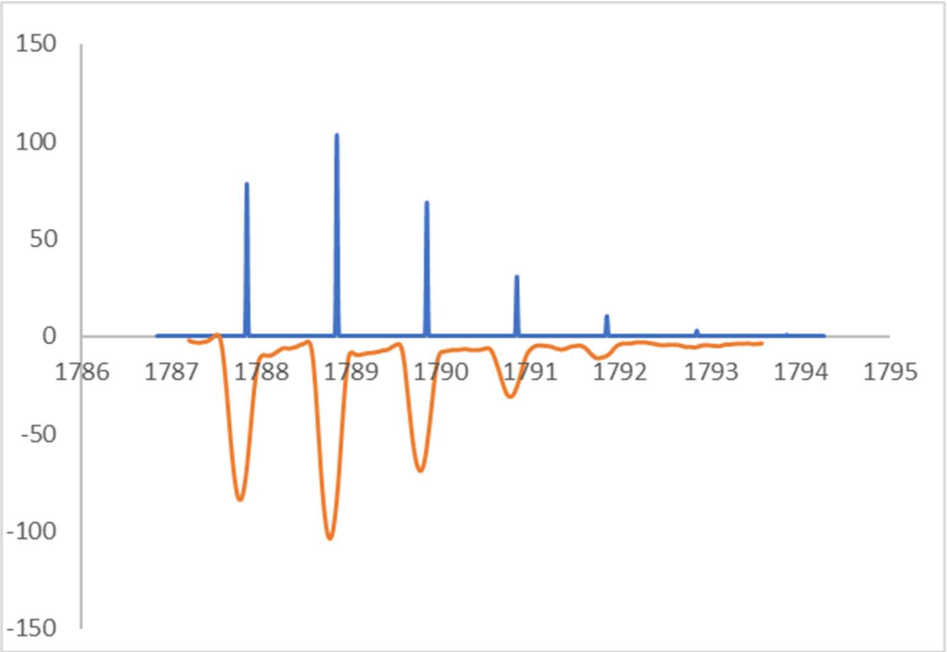

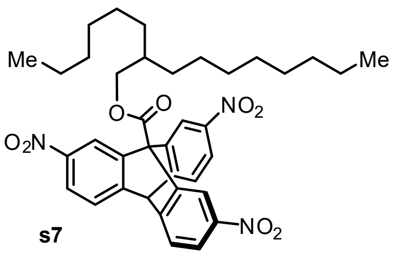

s7

^{1}H NMR (400 MHz, $CDCl_3$)

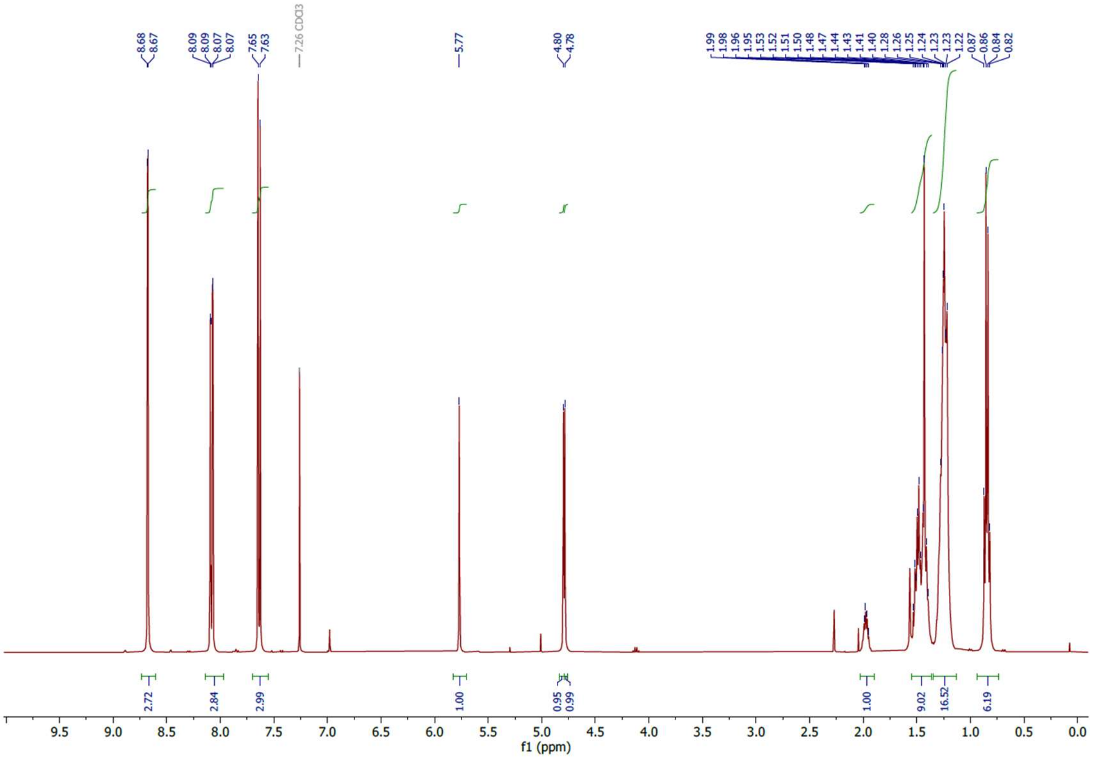

^{13}C NMR (101 MHz, $CDCl_3$)

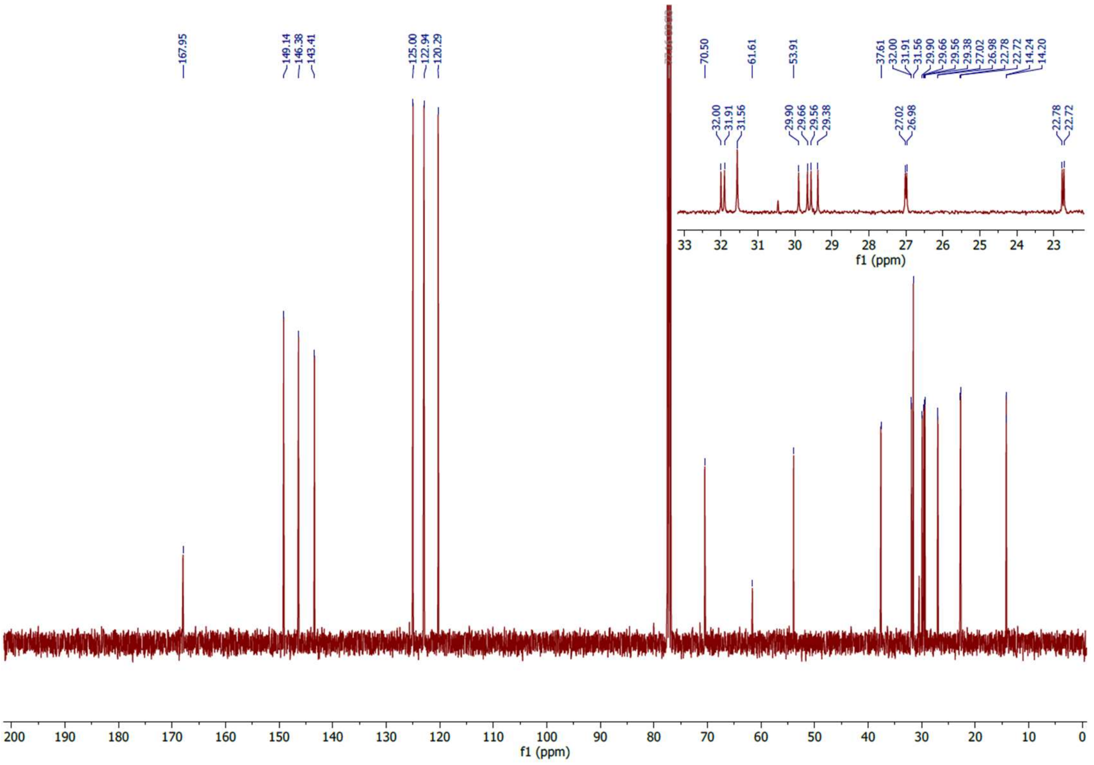

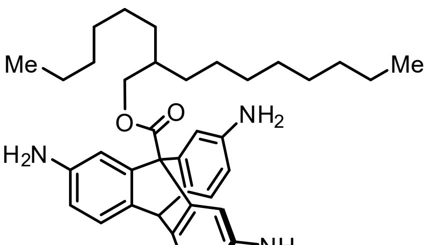

s8

^{1}H NMR (400 MHz, $CDCl_3$)

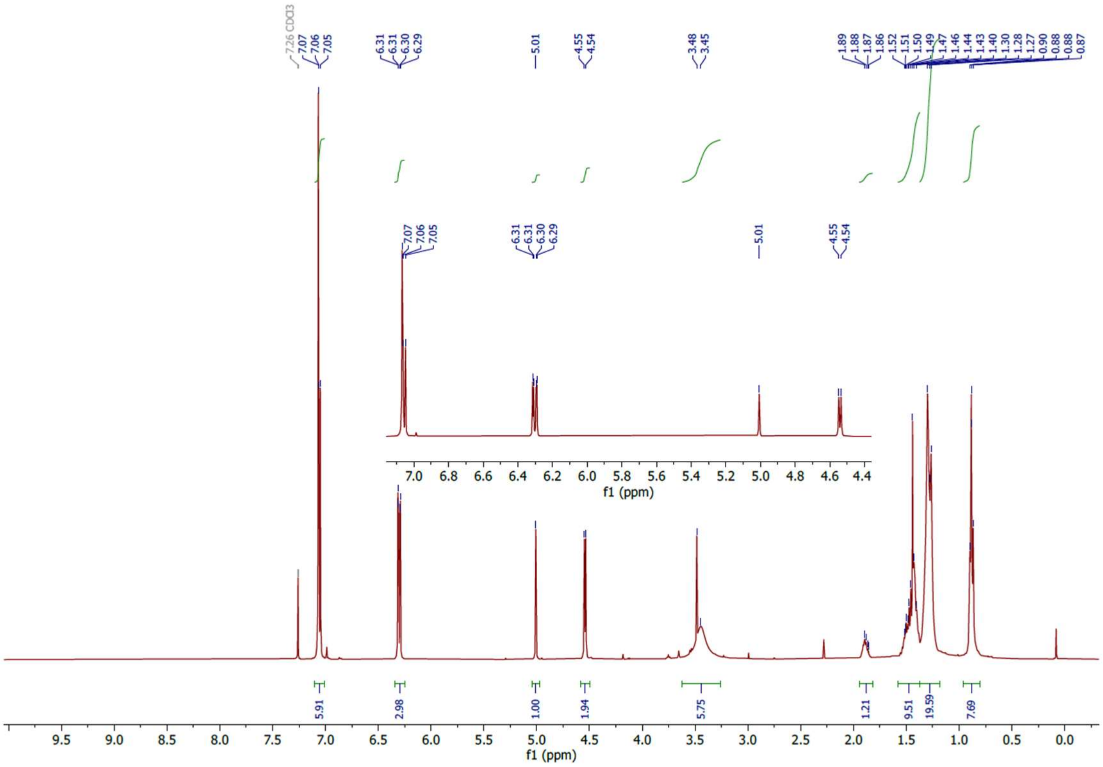

^{13}C NMR (101 MHz, $CDCl_3$)

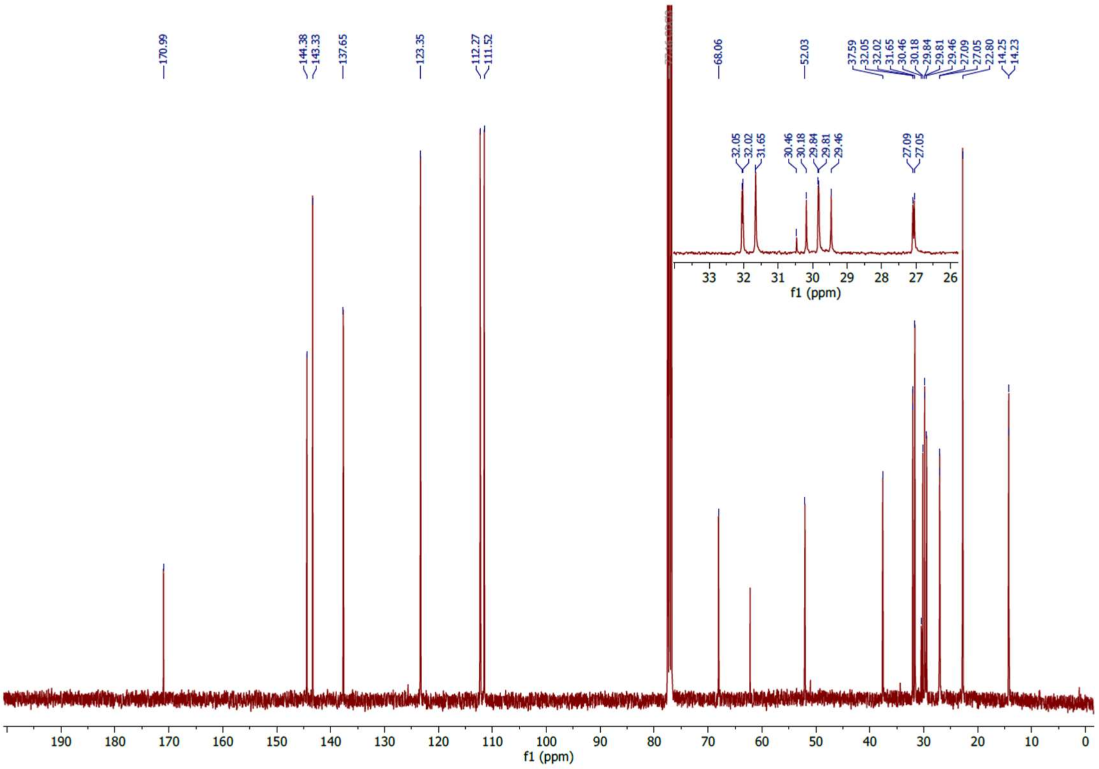

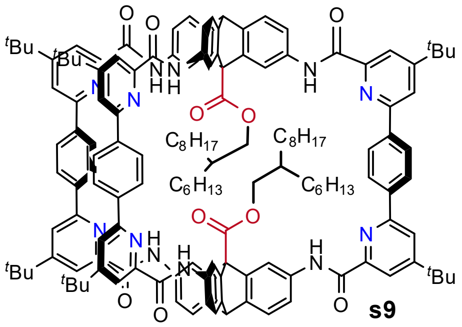

s9

^{1}H NMR (400 MHz, $CDCl_3$)

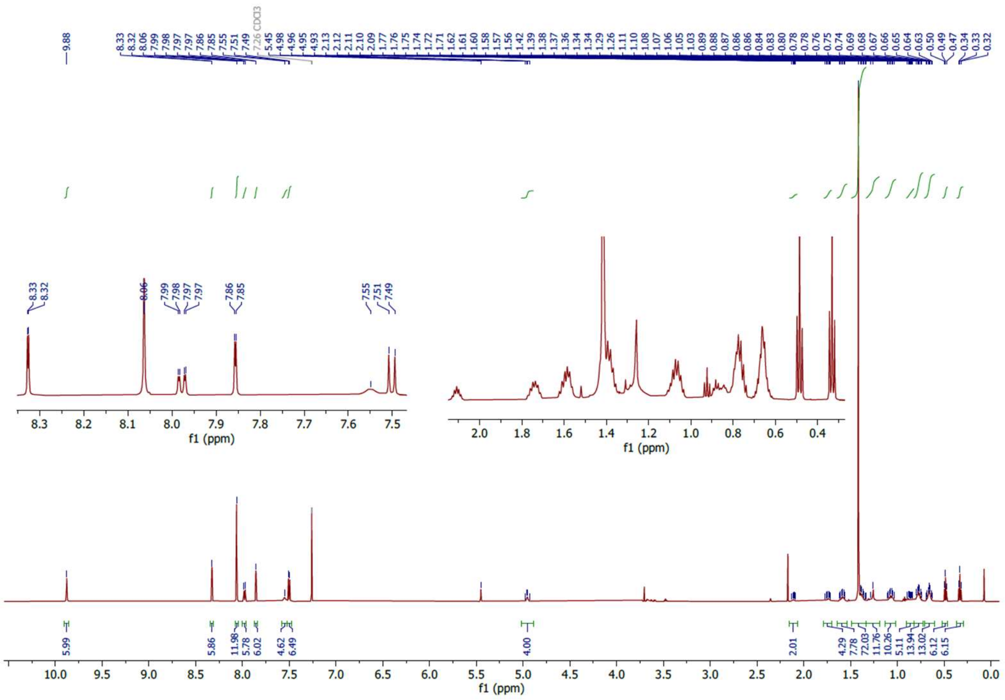

^{13}C NMR (101 MHz, $CDCl_3$)

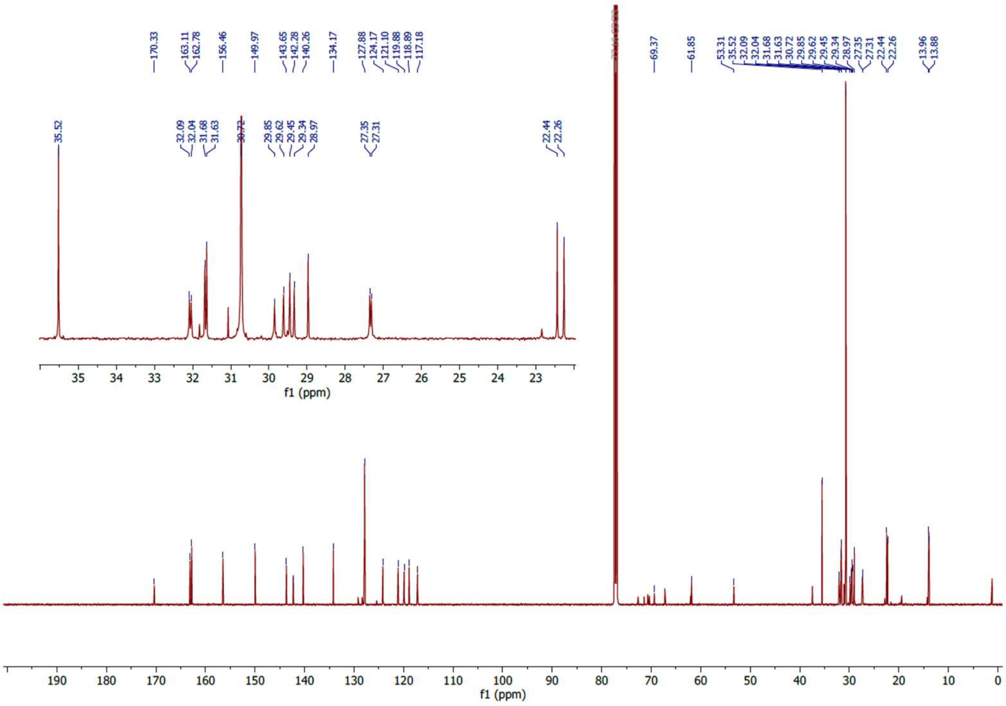

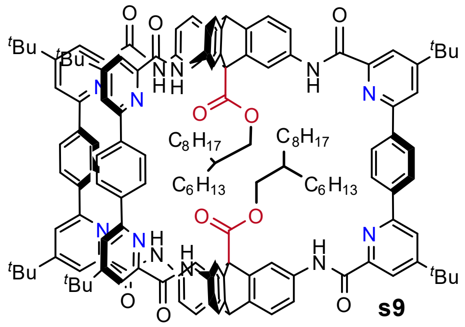

s9

MS *m/z* (MALDI-ToF-RP) (DCTB matrix)

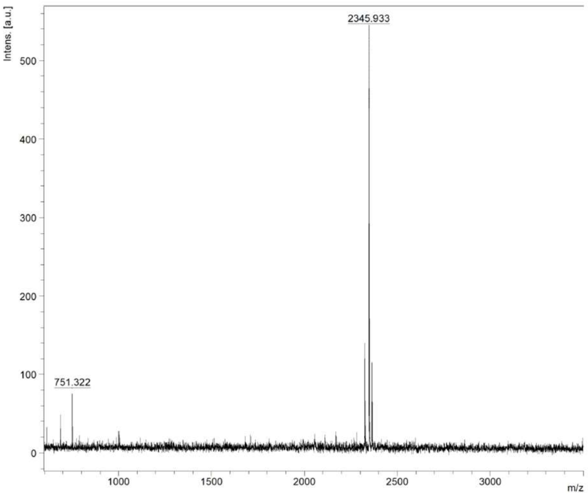

M+H, M+Na

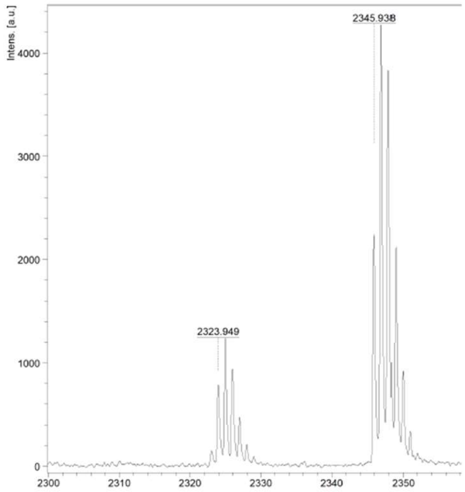

Calculated isotope pattern (top); measured (bottom)

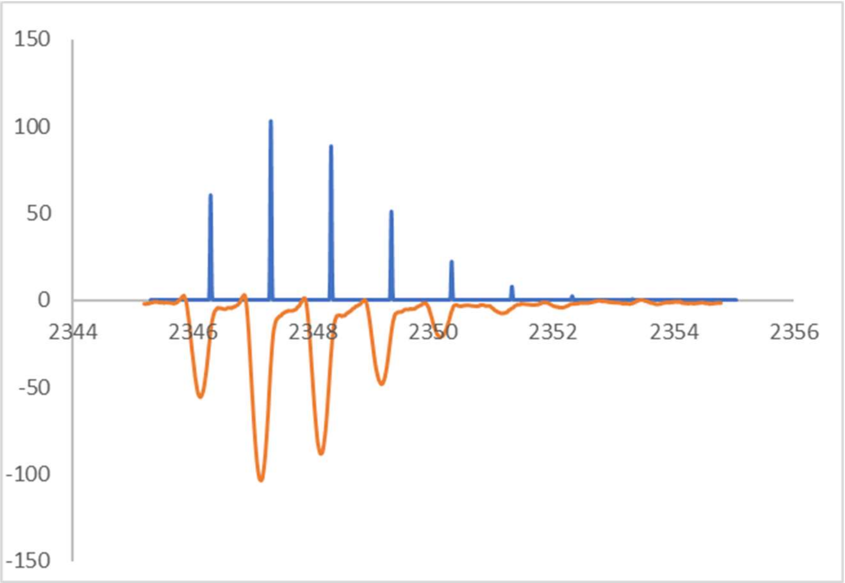

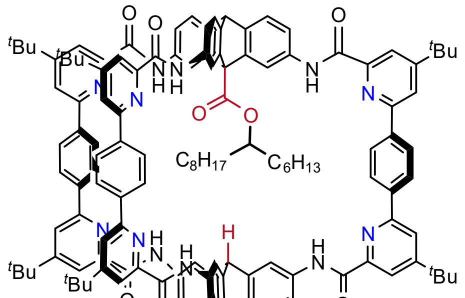

5e

^{1}H NMR (400 MHz, CDCl$_3$)

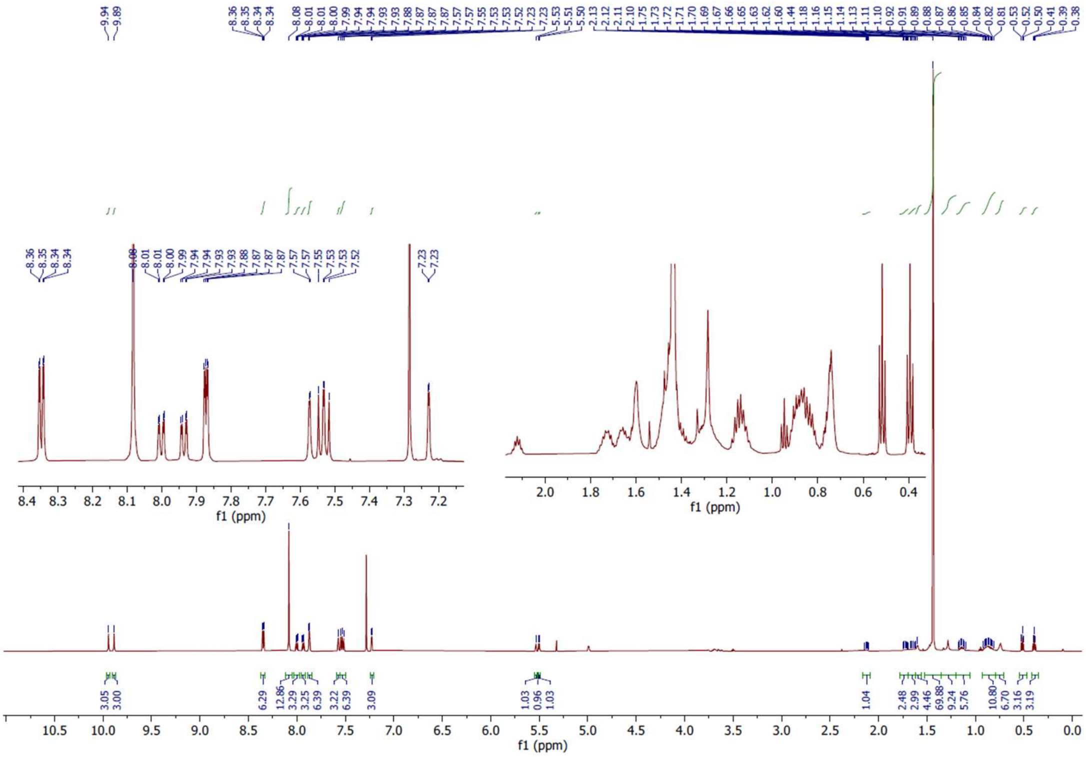

^{13}C NMR (101 MHz, CDCl$_3$)

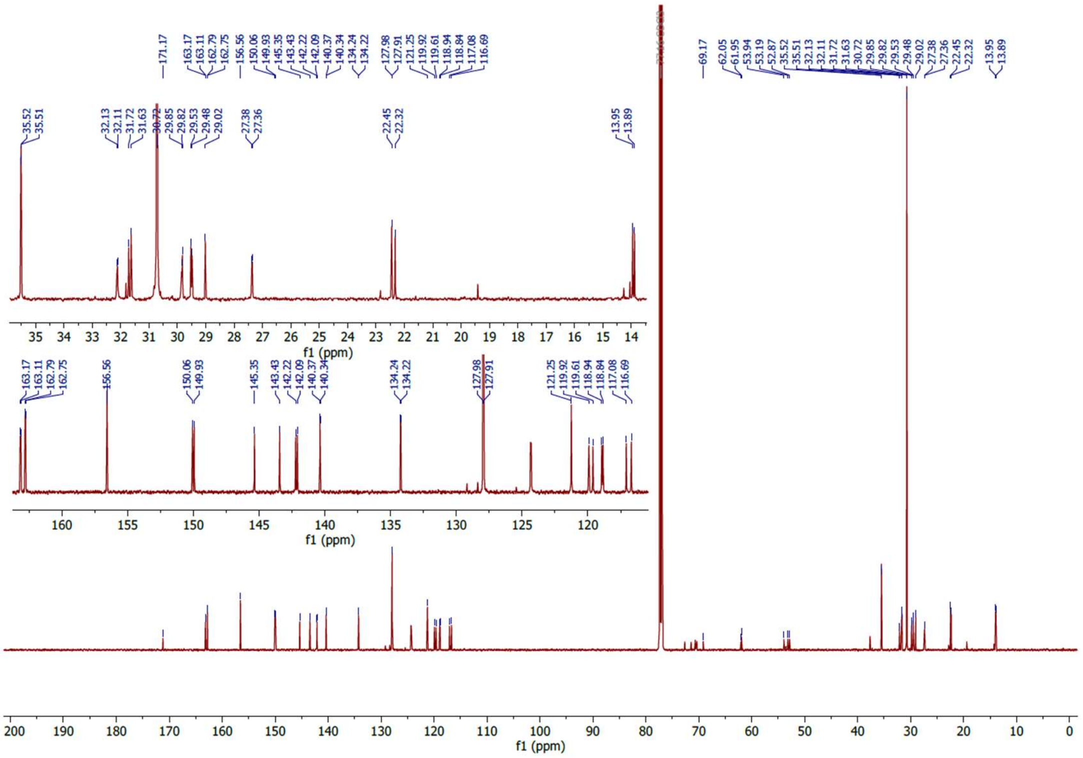

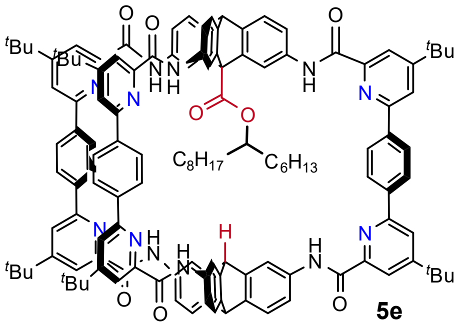

MS *m/z* (MALDI-ToF-RP) (DCTB matrix) M+H, M+Na

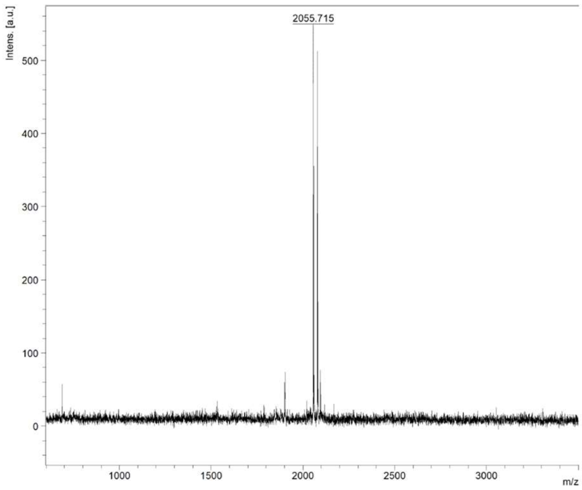

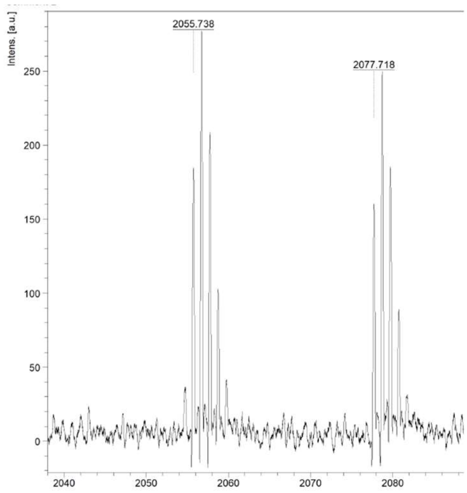

Calculated isotope pattern (top); measured (bottom)

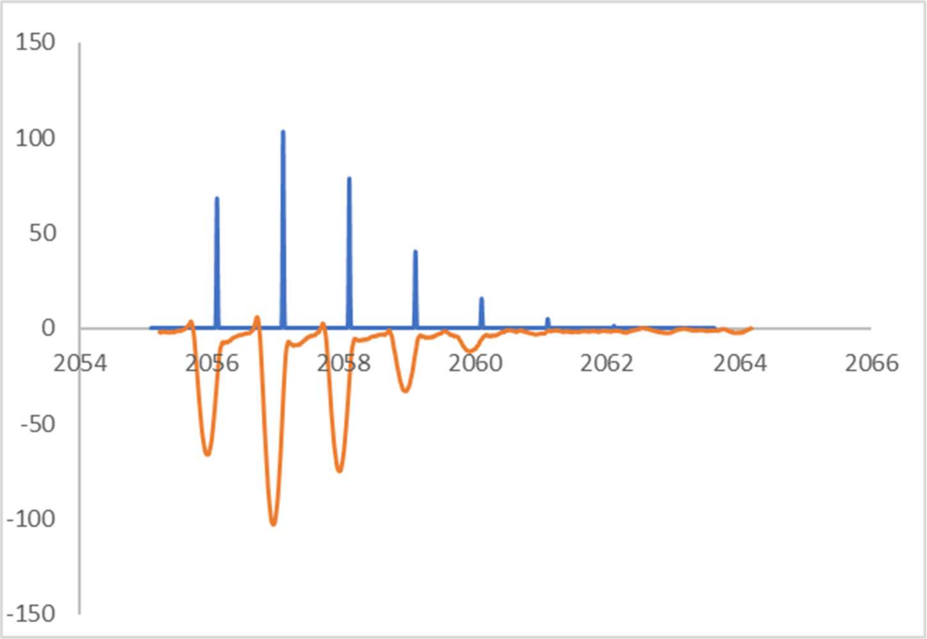

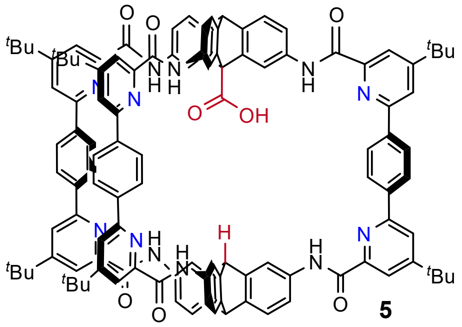

^{1}H NMR (400 MHz, $CDCl_3$)

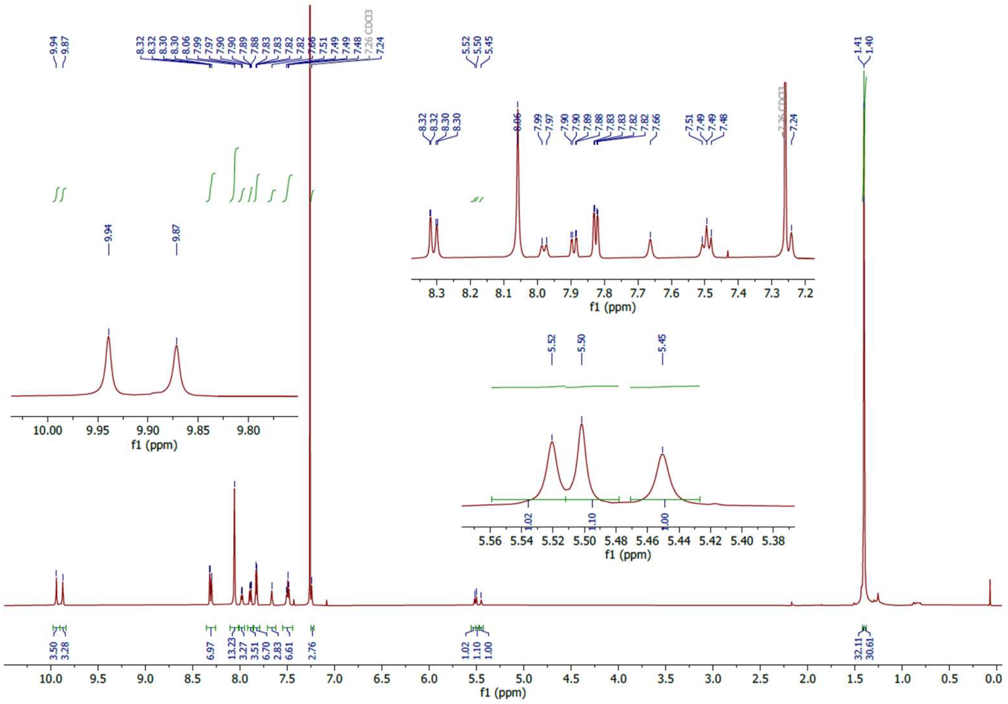

^{13}C NMR (101 MHz, $CDCl_3$)

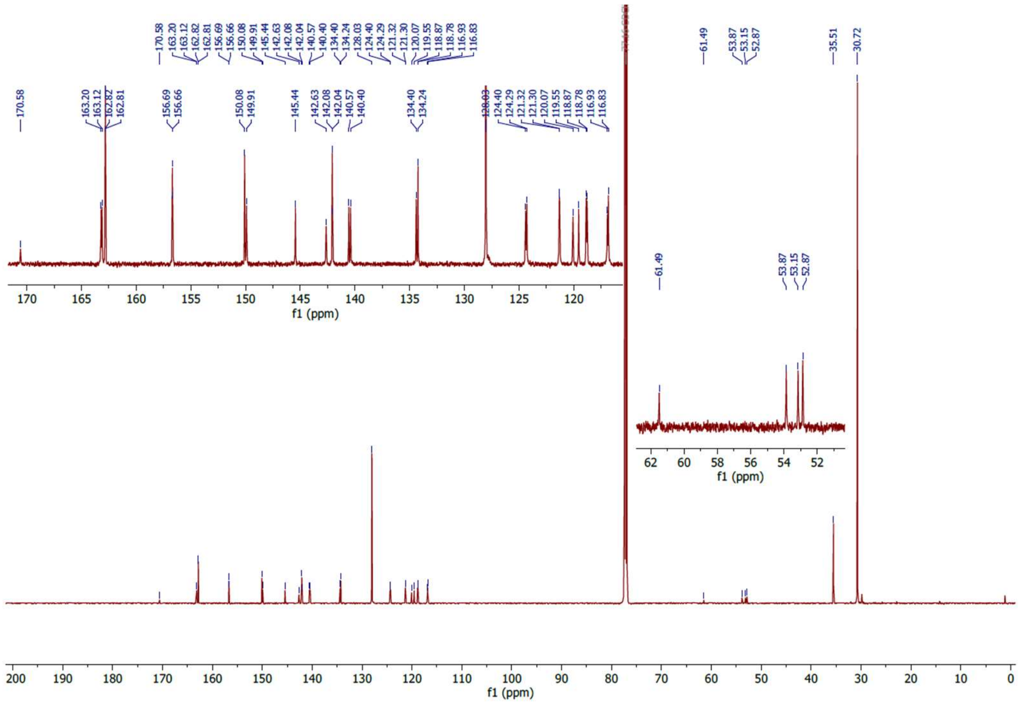

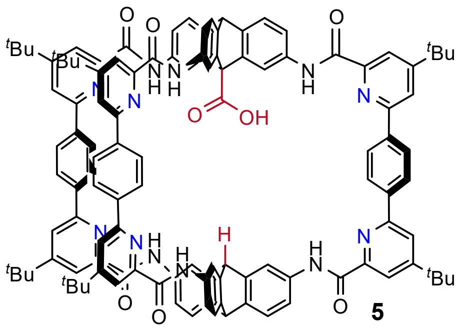

5

MS *m/z* (MALDI-ToF-RP) (DCTB matrix)

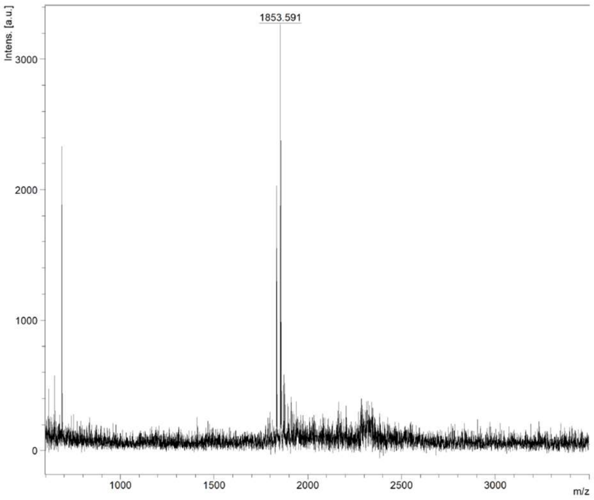

M, M+Na

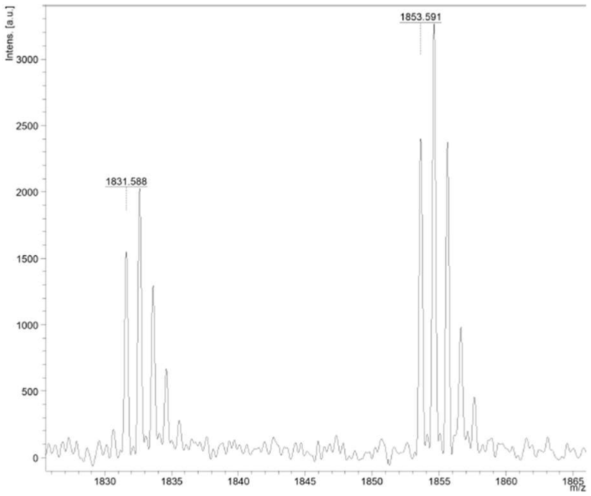

Calculated isotope pattern (top); measured (bottom)

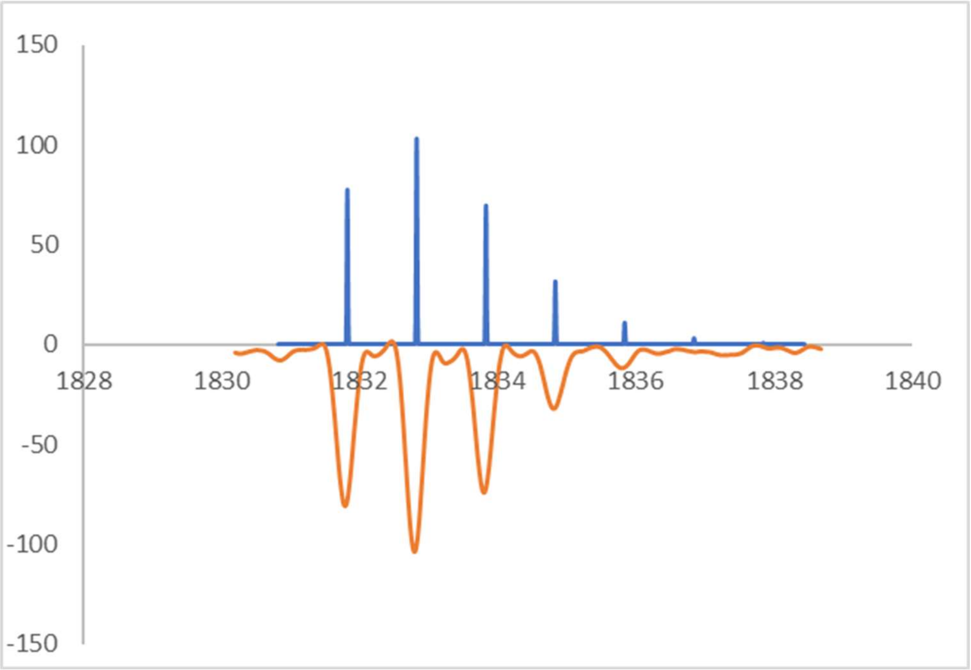

Reference annotations for:

Enzyme-like acyl transfer catalysis in a bifunctional organic cage

In order to facilitate the expeditious locating of supporting claims referenced in the main text, we include an annotated reference justification. The aim of the justification is to aid transparency and save time for readers. The trail includes:

- details of which tables etc quoted values are taken from;
- quotes from the original manuscripts to allow instant localisation of supporting claims using modern computer search functions;
- author comment on groups of references;
- longer footnotes;
- examples of counter-references disputing a claim made in the manuscript, where appropriate;

**INTRODUCTION**	
Enzyme active sites can be approximated as a cavity in which functionality is organized to accelerate chemical reactions by transition state stabilization.[1–4]	Breslow, Cram and others pioneered examples of “artificial enzymes” – catalytic functionalized macrocycles, in which the macrocycle cavity acts as a host, and local functionality performs the reaction. Kraut has argued that all of the various theories of enzyme function come down to transition state binding.
Chemists have explored synthetic cavities as enzyme mimics for decades,[5–11]	Primary literature examples include:<br>Cram[5] (trans-acylations)<br>Breslow[6] hydrolysis (acyl transfer)<br>Rebek[7] (phosphate hydrolysis using a cleft)<br>Sanders[8] (acyl transfer with a cyclic porphyrin trimer)<br>Bender[9] (acyl transfer with cyclodextrins)<br>Mock[10] (curcubituril for cycloaddition catalysis)<br>Cramer[11] (very early use of cyclodextrins to catalyse decarboxylation)
Notable catalytic cavity research has explored functionalized cyclodextrin macrocycles,[2,12,13] other oligomeric macrocycles,[14,15] dendrimers,[16] and rigid clefts,[17]	Breslow demonstrated the catalytic properties of functionalized cyclodextrins. These are macrocycles rather than cages (polymacrocycles), as are curcubiturils and hemicryptophanes.<br>More recently, the encapsulation properties of dendrimers have been applied to reaction acceleration. Rebek showed that rigidly preorganized pairs of carboxylic acids could promote acetal hydrolysis.
although turnover is not always achieved.[18]	Scrimin describes some of the most effective enzyme mimics, and highlights the fact some of them are deactivated on reaction, and so “The term catalyst in some cases may be inappropriately used.”
More recently, metal organic cages[19–26] […] have afforded impressive catalytic transformations in non-covalent assemblies.	Selected examples: Metal organic cages have demonstrated significant rate enhancements, through hydrophobic confinement (Fujita,[19] Raymond/Bergman[21,25] (these examples also features stabilization of charged intermediates)), strong specific binding and

	electrostatic effects (Martí-Centelles/Lusby)[22,24] and binding by multiple acid groups (Hooley)[23] or hydroxyl groups (Nitschke).[20] Schapiro and Klajn have used selective binding inside a cavity dependent on a "cofactor" sensitizer that only operates in the cavity.[26]
and organic capsules[27–35] have afforded impressive catalytic transformations in non-covalent assemblies.	We take organic capsules to be non-covalent organic assemblies; these are distinct from covalent organic cages. There are not many examples of effective capsule catalysts, but Tiefenbacher and coworkers have demonstrated impressive catalysis with a resorcinol capsule, which promotes acid catalysis with unique selectivities due to the confined nature of the reaction.
Extrapolating or embedding ligands to approximate cavities around active metals is also pursued.[36,37]	Diver has reported confined Grubbs-metathesis in a macrocycle, leading to some size-exclusion effects.[36]<br>Reek has an active research programme embedding metals/ligands inside macrocycles and cages to modulate activity.[37]
examples of covalent organic cage[38] systems	Recent examples make use of cages designed by Cooper and co-workers.
facilitating catalysis are limited to systems that encapsulate nanoparticles[39–42] or metals,[43,44] cages with arrays of non-specific hydrogen bond donors[45]/acceptors,[46] and hemicryptophane[15]-confined superbases.[47–49]	Cages can help tune or control nanoparticle behaviour, but it is the nanoparticle imparting the catalytic behavior; likewise with metal-containing organic cages.<br><br>Patra suggests in Fig 3 the role of H-bond donors in the cage promoted reaction of epoxides with CO_2.[45]<br><br>Ayub states:[46] "Moreover, these cages possess nitrogen atoms which can act as hydrogen bond acceptor in the close proximity of reacting atoms."<br><br>Confined superbases can lead to novel selectivity in cages. These hemicryptophanes are synthesised in a semi-stepwise manner.
Unambiguous, cavity-based enzyme-like organocatalysis featuring recognizable bifunctional catalysis modes, cofactors, and covalent and non-covalent activation … remains unreported. [49–51]	Otte's recent review of reactions in functionalized cages[51] describes only two cages[49,50] of a purely organic nature performing catalysis.
Early work in enzyme mimicry tended to rely on arduous multistep synthesis to install functional groups near binding cavities to enhance rates of reaction by increasing effective molarities.[12,52–54]	Breslow reviews cyclodextrin functionalisation. [12]<br><br>Diederich reported a pyruvate oxidase mimics with flavin and thiazolium groups close to a "binding site". [52]<br><br>Cram reported a trans-acylase partial mimic.[54]<br><br>Kirby/Hollfelder discuss the role of effective molarities in this sort of catalysis in Ch3-4.[53]
but are either restricted to mild catalysis	Mastalerz has reported many approaches to

conditions that do not cause them to disassemble, or must undergo a post-synthetic locking procedure[55] to render them stable, a process scarcely available for non-covalent assemblies.[56]	stabilize imine-linked organic cages.[55]<br><br>Nitschke describes the difficulties in post-assembly modification of non-covalently assembled cages.[56]
The cavities must also contain suitable endohedral functionalization[51,57] to direct substrates, or otherwise be restricted to unspecific hydrophobic confinement or proximity-based catalysis,[58] or incremental effects that result from enhanced fragment performance.[48,59]	The majority of "binding site" macrocyclic enzyme model work has utilised the hydrophobic effect to increase reaction rates on a bound substrate. Cages in water are typically very similar.<br><br>In contrast, Reek uses internal guanidine groups to orient substrates and a gold catalyst.[57]<br><br>Lusby discusses many of the origins of /approaches to catalysis.[58]<br><br>Some cages take advantage of an enhanced version of the reactivity of the monomers: The authors of a cage-based photocatalysis paper state:[59]<br>"With the help of cage based photocatalyst and within a short time of ca. 15.0 min, >99% benzylamine molecules have been converted into *N*-benzylidenebenzylamine (based on the NMR data; Table 2). In contrast, a longer reaction time of 20.0 and 25.0 min becomes necessary for the monomer photocatalysts, H_2CBPP and TPP, respectively, to convert >99% benzylamine molecules to target product. This, in cooperation with the TA and ESR spectroscopic results as mentioned above, confirms not only the prominent photocatalytic performance of organic cage under visible light irradiation but also the long triplet lifetime-related promotion mechanism."<br><br>Control experiments often demonstrate how fragments of cages perform only marginally less well than when the fragments are incorporated into a cage, see Table 1 (entry 3, 12, 13).[48]
In our efforts to design stable, soluble organic cages with internal functionality, we recently reported[60] the synthesis of robust amide-linked organic cages featuring a pair of endohedral antipodal carboxylic acids that resemble aspartyl proteases and glycoside hydrolases (like lysozyme).[61]	We believe this to be the first stable, self-assembled amide organic cage with endohedral functionality (i.e. where functionality means a chemical group used specifically for a function, and not just structurally or passively.)<br><br>An enormous number of enzymes feature this dicarboxylic acid motif in active sites.
This work, in which we oxidatively trap imine assemblies as amide cages *in situ*, extended cage post-functionalization methodologies developed by Mastalerz,[55,62,63] which are gaining popularity for accessing functional	Mastalerz has pioneered and adapted many stabilization techniques, and applied them to cages, notably imine cages. The resulting stability of the cages allow significant post-functionalization reactions.

organic cages.[64]	Mastalerz has applied Pinnick oxidation conditions to previously isolated imine cages to access amide cages; our contribution is to trap metastable assemblies in a single pot, which allows more soluble frameworks to be accessed. This allowed us to develop, expand and functionalize triptycene cage topologies first synthesized by Mastalerz.<br><br>Recently, Liu has utilized Mastalerz's Pinnick oxidation conditions to synthesize a macrocycle with affinity for glucose.[64]
We now report a bifunctional organic cage (**Figure 1d**) that realizes well-characterized enzyme-like acyl-transfer catalysis[65–71]	Acyl transfer catalysis continues to attract enormous research efforts due to its importance, both in organocatalysis and biocatalysis.<br>Shiina describes a useful small molecule modified benzotetramisole-type catalyst ((S)-β-Np-BTM) for kinetic resolution using acyl transfer.[65,66]<br>Triazole small molecule catalysts,[67] spiroligozymes,[68] and chiral DMAP molecules[69] promote acyl transfer by nucleophilic catalysis. Aminocyclodextrins have also been reported.[70] Schnepel and Flitsch aim to design enzymes capable of catalyzing amide formation. [71]
contingent on precisely oriented functional groups and an acyl carrier in a fashion reminiscent of the ping-pong mechanism observed in some proteases and transferases… [1,72,73]	Figure 9 [72] shows a bifunctional activation for an acyl transfer mechanism using a covalent ester intermediate in t-RNA synthetase.<br><br>HLyC covalently transfers a fatty acid from an acyl carrier protein to a histidine, unlike most acyl transferases, before transferring it on to a nucleophile (Figure 14).[73]<br><br>Serine proteases covalently accept the acyl group from a peptide bond, before hydrolysis with a water nucleophile.[1]
the enzyme-like nature of the process,[53]	Kirby and Hollfelder set out criteria for an "enzyme mimic" in the intro in this book. They also discuss acyl transfer mechanisms.
**RESULTS AND DISCUSSION**	
The glycoside hydrolase enzyme, lysozyme … requires a mixed carboxylate/carboxylic acid pair to achieve activity.[74]	The active site is depicted in Fig 1.
More generally, enzymes enlist proximal and confined[75] basic functionality to promote dynamic formation of carboxylate species that can drive reactivity too slow to occur in the protonated form,[53] or provide electrostatic transition state stabiliziation.[76]	List has argued that "confinement" (unique chemical behavior in an active site) unifies selective catalysis.[75]<br><br>"Deprotonation" may be more akin to proton-shifts/shuttles observed in "proton wires"[35]
prevents acid-base neutralization interactions that would predominate with flexible or unconfined functionality.[51]	Acid/base quenching in free molecules is easy to understand. Otte outlines another advantage of being able to rigidly hold functionality apart: "A synthetic challenge in the design of iron carboxyl complexes is to prevent the formation

	of carboxyl bridge dimeric species."
hexapyridine dimethyl ester cage **2**…[77]	In related work, we discuss the structure and synthesis of cage 2, but not cage 1.
accessed using our previously developed one-pot imine self-assembly/Pinnick oxidation strategy (58–70% **1**, 2 steps).[60]	See above [60]
Cognizant of the similarities between cage **1** and "multifunctional" enzyme active sites,[78] we sought to identify possible cage-based catalytic manifolds. Proteases like chymotrypsin[79,80] cleave peptide bonds via a ping-pong mechanism[81] in which the enzyme becomes acylated before transferring the acyl group to the nucleophile (water) in a second step (**Figure 1a**). In contrast, acyl CoA coenzymes[82] are acyl carriers and consumed reagents that provide reactive acyl groups to active sites, although rarely via a ping-pong mechanism.[73]	We initially considered comparing the anhydride to cofactors (perhaps better: coenzymes), such as acetyl-CoA.[78] However, these groups rarely covalently transfer their acyl groups to the enzyme before transfer to the substrate. The two-stage transfer mechanism, such as that seen in proteases,[79,80] is often referred to as a ping-pong bi bi mechanism.[81]<br>We are grateful to a reviewer for guiding this discussion.
Enzyme mimic candidates, such as hexaamide cage **1**, have a clear niche[53]	To date, synthetic systems can tolerate a far wider range of conditions, substrates and reaction types than enzymes. Scrimin advocates that: "Rather, it could be very important to develop catalysts for reactions not catalyzed by natural enzymes perhaps operating under conditions hostile to a protein."
Like cage **2,**[77] in the crystal structure of cage **1Ac$_1$**, all amide carbonyl units have externally projected oxygen atoms (in contrast to cage **3**)[60,83]	In recent work,[77] we discuss the structure and synthesis of cage **2** and cage **3**, but not cage **1**. We previously reported the dimethyl ester of cage **3**.[60]<br>We label the "all carbonyls out" conformers as **C13** (00-00-00). See SI.<br>In recent work, we also discuss the effect of the amide carbonyls on precise cavity control in the solid state.[83]
… as a result of pyridine/amide interactions (the pyridine lone pair interacts more favorably with the amide NH group than the amide carbonyl).[77,84]	Hamilton has previously described the conformation preferences of 2-pyridyl amides. We discuss these conformational preferences in recent work.[77]
Symmetry reduction of self-assembled organic cages remains rare and valuable.[77]	In forthcoming work, we discuss the scarcity of low symmetry organic cages and approaches around them.
which has only 4 externally projected oxygen atoms.[60]	Conformer **C9** was observed in the crystal structure, with amide orientations: 00-01-10, and **C5** (01-01-10) predicted computationally.
The rate-acceleration is therefore entirely provided by enthalpic stabilization of the transition state (i.e. transition state binding).[53]	Kirby and Hollfelder discuss transition state binding in Chapter 2.
(ii) a cage carboxylate, formed by proton transfer to a cage pyridine or anhydride, accelerates deprotonation of the alcohol as it attacks the acyl group (**Figure 7aii**);[85–87]	DMAP catalyzed acylations have been proposed to involve an additional carboxylate assisted contribution.[85,87]
Cavity-based activation of a metastable acyl carrier is proposed to occur in acyl transferases.[88]	Acetyl coenzyme A becomes an enhanced acyl donor in the relevant active sites.
but a proton shuttle mechanism from a species with more zwitterionic character is also	See mechanisms d and e in Figure 3. [89]

plausible, as hypothesized in ribosome acyl transfer mechanisms.[89]	
In contrast to our original motivation, the cage **1** internal pyridine group basicity … may be enhanced by amide group rotation,[90] **Figure S42.**	Protonation of a pyridine adjacent to a relevant amide is accompanied by carbonyl rotation in a pair of reported crystal structures (Fig 1 and Fig 2).
Instead, a structural role for pyridine likely dominates: without the pyridyl control over the amide orientation,[77,84] the ground state of cage **3** has at least 2-3 carbonyl units projected inwards,[60,77] leading to an increased acid-acid distance (**1**: r_{cc} = 6.6 Å; **3**: r_{cc} = 8.8 Å)	Our recent work discusses the cavity height and conformation changes for cages **1** and **3.** It is likely that a mixture of **C5** (3 inwards facing carbonyl groups) and **C9** (2 inwards facing carbonyl groups) exists for cage **3.**

Main Text References:

(1) Bugg, T. D. H. *Introduction to Enzyme and Coenzyme Chemistry: Third Edition*; John Wiley and Sons, 2012. https://doi.org/10.1002/9781118348970.

(2) Breslow, Ronald. *Artificial Enzymes*; Wiley-VCH, 2006. https://doi.org/10.1002/3527606645.

(3) Kraut, J. How Do Enzymes Work? *Science (1979)* **1988**, *242* (4878), 533–540. https://doi.org/10.1126/science.3051385.

(4) Raynal, M.; Ballester, P.; Vidal-Ferran, A.; Van Leeuwen, P. W. N. M. Supramolecular Catalysis. Part 2: Artificial Enzyme Mimics. *Chem Soc Rev* **2014**, *43* (5), 1734–1787. https://doi.org/10.1039/C3CS60037H.

(5) Chao, Y.; Cram, D. J. Catalysis and Chiral Recognition Through Designed Complexation of Transition States in Transacylations of Amino Ester Salts. *J Am Chem Soc* **1976**, *98* (4), 1015–1017. https://doi.org/10.1021/JA00420A026.

(6) Breslow, R.; Overman, L. E. An “Artificial Enzyme” Combining a Metal Catalytic Group and a Hydrophobic Binding Cavity. *J Am Chem Soc* **1970**, *92* (4), 1075–1077. https://doi.org/10.1021/JA00707A062.

(7) Tsao, B. L.; Pieters, R. J.; Rebek, J. Convergent Functional Groups. 16. Hydrolysis of Phosphate Triesters by a Novel Cleft. Influence of Binding on Overall Rate Acceleration. *J Am Chem Soc* **1995**, *117* (8), 2210–2213. https://doi.org/10.1021/JA00113A010.

(8) Mackay, L. G.; Wylie, R. S.; Sanders, J. K. M. Catalytic Acyl Transfer by a Cyclic Porphyrin Trimer: Efficient Turnover without Product Inhibition. *J Am Chem Soc* **1994**, *116* (7), 3141–3142. https://doi.org/10.1021/JA00086A061.

(9) Komiyama, M.; Bender, M. L. Cyclodextrin-Catalyzed Hydrolyses of Acetanilides. *J Am Chem Soc* **1977**, *99* (24), 8021–8024. https://doi.org/10.1021/JA00466A040.

(10) Mock, W. L.; Irra, T. A.; Wepsiec, J. P.; Manimaran, T. L. Cycloaddition Induced by Cucurbituril. A Case of Pauling Principle Catalysis. *Journal of Organic Chemistry* **1983**, *48* (20), 3619–3620. https://doi.org/10.1021/JO00168A070.

(11) Cramer, F.; Kampe, W. Inclusion Compounds. XVII. Catalysis of Decarboxylation by Cyclodextrins. A Model Reaction for the Mechanism of Enzymes. *J Am Chem Soc* **1965**, *87* (5), 1115–1120. https://doi.org/10.1021/JA01083A031.

(12) Breslow, R.; Dong, S. D. Biomimetic Reactions Catalyzed by Cyclodextrins and Their Derivatives. *Chem Rev* **1998**, *98* (5), 1997–2011. https://doi.org/10.1021/cr970011j.

(13) Breslow, R. Artificial Enzymes. *Science (1979)* **1982**, *218* (4572), 532–537. https://doi.org/10.1126/science.7123255.

(14) Assaf, K. I.; Nau, W. M. Cucurbiturils: From Synthesis to High-Affinity Binding and Catalysis. *Chem Soc Rev* **2014**, *44* (2), 394–418. https://doi.org/10.1039/C4CS00273C.

(15) Zhang, D.; Martinez, A.; Dutasta, J. P. Emergence of Hemicryptophanes: From Synthesis to Applications for Recognition, Molecular Machines, and Supramolecular Catalysis. *Chem Rev* **2017**, *117* (6), 4900–4942. https://doi.org/10.1021/acs.chemrev.6b00847.

(16) Kofoed, J.; Reymond, J. L. Dendrimers as Artificial Enzymes. *Curr Opin Chem Biol* **2005**, *9* (6), 656–664. https://doi.org/10.1016/J.CBPA.2005.10.013.

(17) Rebek, J. Clefts as Receptor and Enzyme Analogues. *Ciba Foundation symposium*. John Wiley & Sons, Ltd 1991, pp 98–114. https://doi.org/10.1002/9780470514085.ch7.

(18) Lyu, Y.; Scrimin, P. Mimicking Enzymes: The Quest for Powerful Catalysts from Simple Molecules to Nanozymes. *ACS Catal* **2021**, *11*, 11501–11509. https://doi.org/10.1021/acscatal.1c01219.

(19) Yoshizawa, M.; Tamura, M.; Fujita, M. Diels-Alder in Aqueous Molecular Hosts: Unusual Regioselectivity and Efficient Catalysis. *Science (1979)* **2006**, *312* (5771), 251–254. https://doi.org/10.1126/science.1124985.

(20) Bolliger, J. L.; Belenguer, A. M.; Nitschke, J. R. Enantiopure Water-Soluble [Fe4L6] Cages: Host-Guest Chemistry and Catalytic Activity. *Angewandte Chemie - International Edition* **2013**, *52* (31), 7958–7962. https://doi.org/10.1002/anie.201302136.

(21) Hastings, C. J.; Pluth, M. D.; Bergman, R. G.; Raymond, K. N. Enzymelike Catalysis of the Nazarov Cyclization by Supramolecular Encapsulation. *J Am Chem Soc* **2010**, *132* (20), 6938–6940. https://doi.org/10.1021/ja102633e.
(22) Martí-Centelles, V.; Lawrence, A. L.; Lusby, P. J. High Activity and Efficient Turnover by a Simple, Self-Assembled "Artificial Diels–Alderase." *J Am Chem Soc* **2018**, *140* (8), 2862–2868. https://doi.org/10.1021/jacs.7b12146.
(23) Holloway, L. R.; Bogie, P. M.; Lyon, Y.; Ngai, C.; Miller, T. F.; Julian, R. R.; Hooley, R. J. Tandem Reactivity of a Self-Assembled Cage Catalyst with Endohedral Acid Groups. *J Am Chem Soc* **2018**, *140* (26), 8078–8081. https://doi.org/10.1021/jacs.8b03984.
(24) Wang, J.; Young, T. A.; Duarte, F.; Lusby, P. J. Synergistic Noncovalent Catalysis Facilitates Base-Free Michael Addition. *J Am Chem Soc* **2020**, *142* (41), 17743–17750. https://doi.org/10.1021/jacs.0c08639.
(25) Pluth, M. D.; Bergman, R. G.; Raymond, K. H. Acid Catalysis in Basic Solution: A Supramolecular Host Promotes Orthoformate Hydrolysis. *Science (1979)* **2007**, *316* (5821), 85–88. https://doi.org/10.1126/science.1138748.
(26) Gemen, J.; Church, J. R.; Ruoko, T. P.; Durandin, N.; Białek, M. J.; Weißenfels, M.; Feller, M.; Kazes, M.; Odaybat, M.; Borin, V. A.; Kalepu, R.; Diskin-Posner, Y.; Oron, D.; Fuchter, M. J.; Priimagi, A.; Schapiro, I.; Klajn, R. Disequilibrating Azobenzenes by Visible-Light Sensitization under Confinement. *Science (1979)* **2023**, *381* (6664), 1357–1363. https://doi.org/10.1126/science.adh9059.
(27) Syntrivanis, L. D.; Némethová, I.; Schmid, D.; Levi, S.; Prescimone, A.; Bissegger, F.; Major, D. T.; Tiefenbacher, K. Four-Step Access to the Sesquiterpene Natural Product Presilphiperfolan-1β-Ol and Unnatural Derivatives via Supramolecular Catalysis. *J Am Chem Soc* **2020**, *142* (12), 5894–5900. https://doi.org/10.1021/jacs.0c01464.
(28) Heilmann, M.; Knezevic, M.; Piccini, G.; Tiefenbacher, K. Understanding the Binding Properties of Phosphorylated Glycoluril-Derived Molecular Tweezers and Selective Nanomolar Binding of Natural Polyamines in Aqueous Solution. *Org Biomol Chem* **2021**, *19* (16), 3628–3633. https://doi.org/10.1039/d1ob00379h.
(29) Zhang, Q.; Tiefenbacher, K. Terpene Cyclization Catalysed inside a Self-Assembled Cavity. *Nature Chemistry 2015 7:3* **2015**, *7* (3), 197–202. https://doi.org/10.1038/nchem.2181.
(30) Merget, S.; Catti, L.; Piccini, G.; Tiefenbacher, K. Requirements for Terpene Cyclizations inside the Supramolecular Resorcinarene Capsule: Bound Water and Its Protonation Determine the Catalytic Activity. *J Am Chem Soc* **2020**, *142* (9), 4400–4410. https://doi.org/10.1021/jacs.9b13239.
(31) Catti, L.; Zhang, Q.; Tiefenbacher, K. Advantages of Catalysis in Self-Assembled Molecular Capsules. *Chemistry - A European Journal* **2016**, *22* (27), 9060–9066. https://doi.org/10.1002/chem.201600726.
(32) Zhang, Q.; Catti, L.; Tiefenbacher, K. Catalysis inside the Hexameric Resorcinarene Capsule. *Acc Chem Res* **2018**, *51* (9), 2107–2114. https://doi.org/https://doi.org/10.1021/acs.accounts.8b00320.
(33) Kang, J.; Rebek, J. Acceleration of a Diels-Alder Reaction by a Self-Assembled Molecular Capsule. *Nature* **1997**, *385* (6611), 50–52. https://doi.org/10.1038/385050a0.
(34) Zhang, W.; Cheng, G.; Haller, G. L.; Liu, Y.; Lercher, J. A. Rate Enhancement of Acid-Catalyzed Alcohol Dehydration by Supramolecular Organic Capsules. *ACS Catal* **2020**, *10* (22), 13371–13376. https://doi.org/10.1021/acscatal.0c03625.
(35) Li, T. R.; Huck, F.; Piccini, G. M.; Tiefenbacher, K. Mimicry of the Proton Wire Mechanism of Enzymes inside a Supramolecular Capsule Enables β-Selective O-Glycosylations. *Nature Chemistry 2022 14:9* **2022**, *14* (9), 985–994. https://doi.org/10.1038/s41557-022-00981-6.
(36) Zhang, Y.; Diver, S. T. A Macrocyclic Ruthenium Carbene for Size-Selective Alkene Metathesis. *J Am Chem Soc* **2020**, *142* (7), 3371–3374. https://doi.org/10.1021/jacs.0c00081.
(37) García-Simón, C.; Gramage-Doria, R.; Raoufmoghaddam, S.; Parella, T.; Costas, M.; Ribas, X.; Reek, J. N. H. Enantioselective Hydroformylation by a Rh-Catalyst Entrapped in a Supramolecular Metallocage. *J Am Chem Soc* **2015**, *137* (7), 2680–2687. https://doi.org/10.1021/ja512637k.
(38) Tozawa, T.; Jones, J. T. A.; Swamy, S. I.; Jiang, S.; Adams, D. J.; Shakespeare, S.; Clowes, R.; Bradshaw, D.; Hasell, T.; Chong, S. Y.; Tang, C.; Thompson, S.; Parker, J.; Trewin, A.; Bacsa, J.; Slawin, A. M. Z.; Steiner, A.; Cooper, A. I. Porous Organic Cages. *Nat Mater* **2009**, *8* (12), 973–978. https://doi.org/10.1038/nmat2545.
(39) Zhang, Y.; Xiong, Y.; Ge, J.; Lin, R.; Chen, C.; Peng, Q.; Wang, D.; Li, Y. Porous Organic Cage Stabilised Palladium Nanoparticles: Efficient Heterogeneous Catalysts for Carbonylation Reaction of Aryl Halides. *Chemical Communications* **2018**, *54* (22), 2796–2799. https://doi.org/10.1039/C7CC09918E.
(40) Sun, J. K.; Zhan, W. W.; Akita, T.; Xu, Q. Toward Homogenization of Heterogeneous Metal Nanoparticle Catalysts with Enhanced Catalytic Performance: Soluble Porous Organic Cage as a Stabilizer and Homogenizer. *J Am Chem Soc* **2015**, *137* (22), 7063–7066. https://doi.org/10.1021/jacs.5b04029.
(41) Yang, X.; Sun, J. K.; Kitta, M.; Pang, H.; Xu, Q. Encapsulating Highly Catalytically Active Metal Nanoclusters inside Porous Organic Cages. *Nature Catalysis 2018 1:3* **2018**, *1* (3), 214–220. https://doi.org/10.1038/s41929-018-0030-8.
(42) Jiang, S.; Cox, H. J.; Papaioannou, E. I.; Tang, C.; Liu, H.; Murdoch, B. J.; Gibson, E. K.; Metcalfe, I. S.; Evans, J. S. O.; Beaumont, S. K. Shape-Persistent Porous Organic Cage Supported Palladium Nanoparticles as Heterogeneous Catalytic Materials. *Nanoscale* **2019**, *11* (31), 14929–14936. https://doi.org/10.1039/c9nr04553h.

(43) Bete, S. C.; May, L. K.; Woite, P.; Roemelt, M.; Otte, M. A Copper Cage-Complex as Mimic of the PMMO CuC Site. *Angewandte Chemie International Edition* **2022**, *61* (35), e202206120. https://doi.org/10.1002/ANIE.202206120.
(44) Perraud, O.; Sorokin, A. B.; Dutasta, J. P.; Martinez, A. Oxidation of Cycloalkanes by H2O2 Using a Copper–Hemicryptophane Complex as a Catalyst. *Chemical Communications* **2013**, *49* (13), 1288–1290. https://doi.org/10.1039/C2CC37829A.
(45) Hussain, W.; Giri, A.; Patra, A. Organic Nanocages: A Promising Testbed for Catalytic CO2 Conversion. *Sustain Energy Fuels* **2019**, *3* (10), 2567–2571. https://doi.org/10.1039/c9se00394k.
(46) Mukhtar, A.; Sarfaraz, S.; Ayub, K. Organic Transformations in the Confined Space of Porous Organic Cage CC2; Catalysis or Inhibition. *RSC Adv* **2022**, *12* (37), 24397–24411. https://doi.org/10.1039/D2RA03399B.
(47) Yang, J.; Chatelet, B.; Dufaud, V.; Hérault, D.; Michaud-Chevallier, S.; Robert, V.; Dutasta, J.-P.; Martinez, A. Endohedral Functionalized Cage as a Tool to Create Frustrated Lewis Pairs. *Angewandte Chemie* **2018**, *130* (43), 14408–14411. https://doi.org/10.1002/ANGE.201808291.
(48) Chen, H. Y.; Gou, M.; Wang, J. B. De Novo Endo-Functionalized Organic Cages as Cooperative Multi-Hydrogen-Bond-Donating Catalysts. *Chemical Communications* **2017**, *53* (25), 3524–3526. https://doi.org/10.1039/C7CC00938K.
(49) Chatelet, B.; Dufaud, V.; Dutasta, J. P.; Martinez, A. Catalytic Activity of an Encaged Verkade's Superbase in a Base-Catalyzed Diels-Alder Reaction. *Journal of Organic Chemistry* **2014**, *79* (18), 8684–8688. https://doi.org/https://doi.org/10.1021/jo501457d.
(50) Chen, H. Y.; Gou, M.; Wang, J. B. De Novo Endo-Functionalized Organic Cages as Cooperative Multi-Hydrogen-Bond-Donating Catalysts. *Chemical Communications* **2017**, *53* (25), 3524–3526. https://doi.org/10.1039/C7CC00938K.
(51) Otte, M. Reactions in Endohedral Functionalized Cages. *European J Org Chem* **2023**, *26* (18), e202300012. https://doi.org/10.1002/ejoc.202300012.
(52) Mattei, P.; Diederich, F. A Flavo-Thiazolio-Cyclophane as a Functional Model for Pyruvate Oxidase. *Angewandte Chemie International Edition in English* **1996**, *35* (12), 1341–1344. https://doi.org/10.1002/ANIE.199613411.
(53) Kirby, A. J.; Hollfelder, F. *From Enzyme Models to Model Enzymes;* The Royal Society of Chemistry, **2009**. https://doi.org/10.1039/9781847559784.
(54) Cram, D. J.; Lam, P. Y. S.; Ho, S. P. A Transacylase Partial Mimic. *J Am Chem Soc* **1986**, *108* (4), 839–841. https://doi.org/10.1021/JA00264A048.
(55) Mastalerz, M. Porous Shape-Persistent Organic Cage Compounds of Different Size, Geometry, and Function. *Acc Chem Res* **2018**, *51* (10), 2411–2422. https://doi.org/10.1021/acs.accounts.8b00298.
(56) Roberts, D. A.; Pilgrim, B. S.; Nitschke, J. R. Covalent Post-Assembly Modification in Metallosupramolecular Chemistry. *Chem Soc Rev* **2018**, *47* (2), 626–644. https://doi.org/10.1039/C6CS00907G.
(57) Wang, Q. Q.; Gonell, S.; Leenders, S. H. A. M.; Dürr, M.; Ivanovic-Burmazovic, I.; Reek, J. N. H. Self-Assembled Nanospheres with Multiple Endohedral Binding Sites Pre-Organize Catalysts and Substrates for Highly Efficient Reactions. *Nature Chemistry 2015 8:3* **2016**, *8* (3), 225–230. https://doi.org/10.1038/nchem.2425.
(58) Spicer, R. L.; Lusby, P. J. Chapter 2: Catalytic Strategies within the Confined Spaces of Coordination Cages. In *Monographs in Supramolecular Chemistry*; Royal Society of Chemistry, 2021; Vol. 2021-Janua, pp 29–69. https://doi.org/10.1039/9781788019705-00029.
(59) Liu, C.; Liu, K.; Wang, C.; Liu, H.; Wang, H.; Su, H.; Li, X.; Chen, B.; Jiang, J. Elucidating Heterogeneous Photocatalytic Superiority of Microporous Porphyrin Organic Cage. *Nat Commun* **2020**, *11* (1), 1–9. https://doi.org/10.1038/s41467-020-14831-x.
(60) Andrews, K. G.; Christensen, K. E. Access to Amide-linked Organic Cages by in Situ Trapping of Metastable Imine Assemblies: Solution Phase Bisamine Recognition. *Chemistry – A European Journal* **2023**, e202300063. https://doi.org/10.1002/chem.202300063.
(61) Vocadlo, D. J.; Davies, G. J.; Laine, R.; Withers, S. G. Catalysis by Hen Egg-White Lysozyme Proceeds via a Covalent Intermediate. *Nature* **2001**, *412* (6849), 835–838. https://doi.org/10.1038/35090602.
(62) Lauer, J. C.; Bhat, A. S.; Barwig, C.; Fritz, N.; Kirschbaum, T.; Rominger, F.; Mastalerz, M. [2+3] Amide Cages by Oxidation of [2+3] Imine Cages – Revisiting Molecular Hosts for Highly Efficient Nitrate Binding. *Chemistry - A European Journal* **2022**, *28* (51), e202201527. https://doi.org/10.1002/chem.202201527.
(63) Schneider, M. W.; Oppel, I. M.; Mastalerz, M. Exo-Functionalized Shape-Persistent [2+3] Cage Compounds: Influence of Molecular Rigidity on Formation and Permanent Porosity. *Chemistry - A European Journal* **2012**, *18* (14), 4156–4160. https://doi.org/10.1002/chem.201200032.
(64) Zhai, C.; Xu, C.; Cui, Y.; Wojtas, L.; Liu, W. A Dynamic Approach to Synthetic Lectin for Glucose with Boosted Binding Affinity through C–H Hydrogen Bonds. *Chemistry – A European Journal* **2023**, e202300524. https://doi.org/10.1002/CHEM.202300524.
(65) Shiina, I.; Nakata, K.; Ono, K.; Onda, Y. S.; Itagaki, M. Kinetic Resolution of Racemic α-Arylalkanoic Acids with Achiral Alcohols via the Asymmetric Esterification Using Carboxylic Anhydrides and Acyl-Transfer Catalysts. *J Am Chem Soc* **2010**, *132* (33), 11629–11641. https://doi.org/10.1021/ja103490h.

(66) Nakata, K.; Gotoh, K.; Ono, K.; Futami, K.; Shiina, I. Kinetic Resolution of Racemic 2-Hydroxy-γ-Butyrolactones by Asymmetric Esterification Using Diphenylacetic Acid with Pivalic Anhydride and a Chiral Acyl-Transfer Catalyst. *Org Lett* **2013**, *15* (6), 1170–1173. https://doi.org/10.1021/ol303453j.
(67) Yang, X.; Birman, V. B. Acyl Transfer Catalysis with 1,2,4-Triazole Anion. *Org Lett* **2009**, *11* (7), 1499–1502. https://doi.org/10.1021/ol900098q.
(68) Kheirabadi, M.; Çelebi-Ölçüm, N.; Parker, M. F. L.; Zhao, Q.; Kiss, G.; Houk, K. N.; Schafmeister, C. E. Spiroligozymes for Transesterifications: Design and Relationship of Structure to Activity. *J Am Chem Soc* **2012**, *134* (44), 18345–18353. https://doi.org/10.1021/ja3069648.
(69) Furuta, T.; Kawabata, T. Chiral DMAP-Type Catalysts for Acyl-Transfer Reactions. In *Asymmetric Organocatalysis 1*; List, B., Ed.; Thieme Verlag, 2013; p 497. https://doi.org/10.1055/sos-sd-204-00240.
(70) Ferguson, C. G.; Thatcher, G. R. J. Catalysis and Acceleration of Acyl Transfer by Aminocyclodextrins: A Biomimetic System of Use in Enzyme Modeling and Drug Design. *Org Lett* **1999**, *1* (6), 829–832. https://doi.org/https://doi.org/10.1021/ol9906211.
(71) Schnepel, C.; Pérez, L. R.; Yu, Y.; Angelastro, A.; Heath, R. S.; Lubberink, M.; Falcioni, F.; Mulholland, K.; Hayes, M. A.; Turner, N. J.; Flitsch, S. L. Thioester-Mediated Biocatalytic Amide Bond Synthesis with in Situ Thiol Recycling. *Nature Catalysis 2022 6:1* **2022**, *6* (1), 89–99. https://doi.org/10.1038/s41929-022-00889-x.
(72) Xin, Y.; Li, W.; First, E. A. Stabilization of the Transition State for the Transfer of Tyrosine to TRNATyr by Tyrosyl-TRNA Synthetase. *J Mol Biol* **2000**, *303* (2), 299–310. https://doi.org/10.1006/JMBI.2000.4126.
(73) Röttig, A.; Steinbüchel, A. Acyltransferases in Bacteria. *Microbiology and Molecular Biology Reviews* **2013**, *77* (2), 277–321. https://doi.org/10.1128/MMBR.00010-13.
(74) Sun, D. P.; Liao, D. I.; Remington, S. J. Electrostatic Fields in the Active Sites of Lysozymes. *Proc Natl Acad Sci U S A* **1989**, *86* (14), 5361–5365. https://doi.org/10.1073/pnas.86.14.5361.
(75) Mitschke, B.; Turberg, M.; List, B. Confinement as a Unifying Element in Selective Catalysis. *Chem* **2020**, *6* (10), 2515–2532. https://doi.org/10.1016/J.CHEMPR.2020.09.007.
(76) Warshel, A.; Sharma, P. K.; Kato, M.; Xiang, Y.; Liu, H.; Olsson, M. H. M. Electrostatic Basis for Enzyme Catalysis. *Chemical Reviews*. American Chemical Society August 2006, pp 3210–3235. https://doi.org/10.1021/cr0503106.
(77) Andrews, K. G.; Horton, P. N.; Coles, S. J. Programmable Synthesis of Organic Cages with Reduced Symmetry. *Chem Sci* **2024**, *15* (17), 6536–6543. https://doi.org/10.1039/D4SC00889H.
(78) Jeremy M Berg; John L Tymoczko; Gregory J Gatto; Lubert Stryer. *Biochemistry*, 8th ed.; Macmillan Education: New York, 2015.
(79) Appel, W. Chymotrypsin: Molecular and Catalytic Properties. *Clin Biochem* **1986**, *19* (6), 317–322. https://doi.org/10.1016/S0009-9120(86)80002-9.
(80) Oosterbaan, R. A.; Kunst, P.; Van Rotterdam, J.; Cohen, J. A. The Reaction of Chymotrypsin and Diisopropylphosphofluoridate I. Isolation and Analysis of Diisopropylphosphoryl-Peptides. *Biochim Biophys Acta* **1958**, *27* (C), 549–555. https://doi.org/10.1016/0006-3002(58)90385-8.
(81) Cleland, W. W. The Kinetics of Enzyme-Catalyzed Reactions with Two or More Substrates or Products. III. Prediction of Initial Velocity and Inhibition Patterns by Inspection. *BBA - Biochimica et Biophysica Acta* **1963**, *67* (C), 188–196. https://doi.org/10.1016/0006-3002(63)91816-x.
(82) De Bolster, M. W. G. Glossary of Terms Used in Bioinorganic Chemistry (IUPAC Recommendations 1997). *Pure and Applied Chemistry* **1997**, *69* (6), 1251-1303. https://doi.org/10.1351/pac199769061251.
(83) Shields, C. E.; Fellowes, T.; Slater, A. G.; Cooper, A.; Andrews, K. G.; Szczypiński, F. T. Exploration of the Polymorphic Solid-State Landscape of an Amide-Linked Organic Cage Using Computation and Automation. *Chemical Communications* **2024**, *60* (47), 6023-6026. https://doi.org/10.1039/D4CC01407C.
(84) Saraogi, I.; Incarvito, C. D.; Hamilton, A. D. Controlling Curvature in a Family of Oligoamide α-Helix Mimetics. *Angewandte Chemie - International Edition* **2008**, *47* (50), 9691–9694. https://doi.org/10.1002/anie.200803778.
(85) Sakakura, A.; Kawajiri, K.; Ohkubo, T.; Kosugi, Y.; Ishihara, K. Widely Useful DMAP-Catalyzed Esterification under Auxiliary Base- and Solvent-Free Conditions. *J Am Chem Soc* **2007**, *129* (47), 14775–14779. https://doi.org/https://doi.org/10.1021/ja075824w.
(86) Hassner, A.; Krepski, L. R.; Alexanian, V. Aminopyridines as Acylation Catalysts for Tertiary Alcohols. *Tetrahedron* **1978**, *34* (14), 2069–2076. https://doi.org/10.1016/0040-4020(78)89005-X.
(87) Guibe-Jampel, E.; Le Corre, G.; Wakselman, M. Is 1-Acetyl-4-Dimethylaminopyridinium Acetate an Intermediate in the Dmap-Catalyzed Acetylation of Tertiary Alcohols? *Tetrahedron Lett* **1979**, *20* (13), 1157–1160. https://doi.org/10.1016/S0040-4039(01)86089-5.
(88) Shirmast, P.; Ghafoori, S. M.; Irwin, R. M.; Abendroth, J.; Mayclin, S. J.; Lorimer, D. D.; Edwards, T. E.; Forwood, J. K. Structural Characterization of a GNAT Family Acetyltransferase from Elizabethkingia Anophelis Bound to Acetyl-CoA Reveals a New Dimeric Interface. *Scientific Reports 2021 11:1* **2021**, *11* (1), 1–9. https://doi.org/10.1038/s41598-020-79649-5.
(89) Leung, E. K. Y.; Suslov, N.; Tuttle, N.; Sengupta, R.; Piccirilli, J. A. The Mechanism of Peptidyl Transfer Catalysis by the Ribosome. *Annu Rev Biochem* **2011**, *80*, 527–555. https://doi.org/10.1146/annurev-biochem-082108-165150.
(90) Ravikumar, K.; Sridhar, B.; Bhujanga Rao, A. K. S.; Pulla Reddy, M. Sorafenib and Its Tosyl-Ate Salt: A Multikinase Inhibitor for Treating Cancer. *Acta Crystallogr C* **2011**, *67* (1), o29–o32. https://doi.org/10.1107/S0108270110047451.
